# Reconstruction and functional analysis of altered molecular pathways in human atherosclerotic arteries

**DOI:** 10.1186/1471-2164-10-13

**Published:** 2009-01-09

**Authors:** Stefano Cagnin, Michele Biscuola, Cristina Patuzzo, Elisabetta Trabetti, Alessandra Pasquali, Paolo Laveder, Giuseppe Faggian, Mauro Iafrancesco, Alessandro Mazzucco, Pier Franco Pignatti, Gerolamo Lanfranchi

**Affiliations:** 1 CRIBI Biotechnology Centre, University of Padova, Padova, Italy; 2 Department of Biology, University of Padova, Padova, Italy; 3 Department of Mother and Child, Biology and Genetics, Section of Biology and Genetics, University of Verona, Verona, Italy; 4 Division of Cardiac Surgery, University of Verona Medical School, Verona, Italy

## Abstract

**Background:**

Atherosclerosis affects aorta, coronary, carotid, and iliac arteries most frequently than any other body vessel. There may be common molecular pathways sustaining this process. Plaque presence and diffusion is revealed by circulating factors that can mediate systemic reaction leading to plaque rupture and thrombosis.

**Results:**

We used DNA microarrays and meta-analysis to study how the presence of calcified plaque modifies human coronary and carotid gene expression. We identified a series of potential human atherogenic genes that are integrated in functional networks involved in atherosclerosis. Caveolae and JAK/STAT pathways, and S100A9/S100A8 interacting proteins are certainly involved in the development of vascular disease. We found that the system of caveolae is directly connected with genes that respond to hormone receptors, and indirectly with the apoptosis pathway.

Cytokines, chemokines and growth factors released in the blood flux were investigated in parallel. High levels of RANTES, IL-1ra, MIP-1alpha, MIP-1beta, IL-2, IL-4, IL-5, IL-6, IL-7, IL-17, PDGF-BB, VEGF and IFN-gamma were found in plasma of atherosclerotic patients and might also be integrated in the molecular networks underlying atherosclerotic modifications of these vessels.

**Conclusion:**

The pattern of cytokine and S100A9/S100A8 up-regulation characterizes atherosclerosis as a proinflammatory disorder. Activation of the JAK/STAT pathway is confirmed by the up-regulation of IL-6, STAT1, ISGF3G and IL10RA genes in coronary and carotid plaques. The functional network constructed in our research is an evidence of the central role of STAT protein and the caveolae system to contribute to preserve the plaque. Moreover, Cav-1 is involved in SMC differentiation and dyslipidemia confirming the importance of lipid homeostasis in the atherosclerotic phenotype.

## Background

Atherosclerosis is a chronic inflammatory disease of the arterial wall, where both innate and adaptive immunologic and inflammatory mechanisms are involved. Several genomic approaches have been applied to understand this paradigmatic multifactorial disease: these include DNA polymorphisms and candidate gene searching, transcriptome and proteome analysis[1]. In particular, over the past few years, a number of studies have analyzed gene expression profiles in plaques from autopsy and surgery[2], symptomatic and asymptomatic patients[3], stable and unstable plaques[4] as well as in mouse models for atherosclerosis[5]. King et al.[6] analyzed differences in gene expression between plaques of different pathological grade and Satterthwaite et al.[7] used gene expression data to identify genes related to inflammation in atherosclerotic coronaries from ischemic heart and dilated cardiomyopathy. Both these studies as those previously cited, aimed to single out risk factors in one definite condition and tissue. Instead, we decided to adopt an approach where experimental gene expression data are integrated by metanalysis and blood protein measurements to analyze a wider range of pathological samples and patients and to obtain a larger picture of gene/protein interactions involved in atherosclerotic process. The interactions found with our work are common to plaques of different vessel location. Interestingly, no study has so far investigated in humans the relationship between plaque gene expression and released circulating factors, but analyzed in association with platelet aggregation in patients undergoing percutaneous intervention[8] that cause blood flux impairment and modulate the inflammatory response. Using high throughput approaches, we analyzed released cytokines and growth factors in the arterial blood of atherosclerotic patients presenting 75% coronary stenosis at least. These experimental data have been used simultaneously for two aims: a) confirmation of specific gene expression results and b) completion of specific altered pathways identified by gene expression analysis. In parallel, we applied DNA microarray and quantitative real-time PCR (qRT-PCR) to identify differentially expressed genes in coronaries with same stenosis and calcified plaque. In our study we used samples derived from whole atherosclerotic biopsies: atherosclerosis is, in fact, the result of pathological interactions of multiple cell types, mediated by body fluids, hence, cell-based studies will only reveal a subset of these interactions. We also applied meta-analysis to correlate the profiles of atherosclerotic coronaries to other profiles obtained in carotids with comparable stenosis. This work has led us to the identification of pleiotropic and epistatic gene interactions and of pathways that appear to contribute to preserve the plaque, but also to enhance the physiological processes sustaining/attenuating its systemic effects. This information has been useful to define molecular networks underlying the atherosclerotic condition. Our findings may have significance for the development of pharmacological approaches against the central nodes of the identified network, for the prevention or attenuation of atherosclerosis effects on human health.

## Results

**Figure 1:**
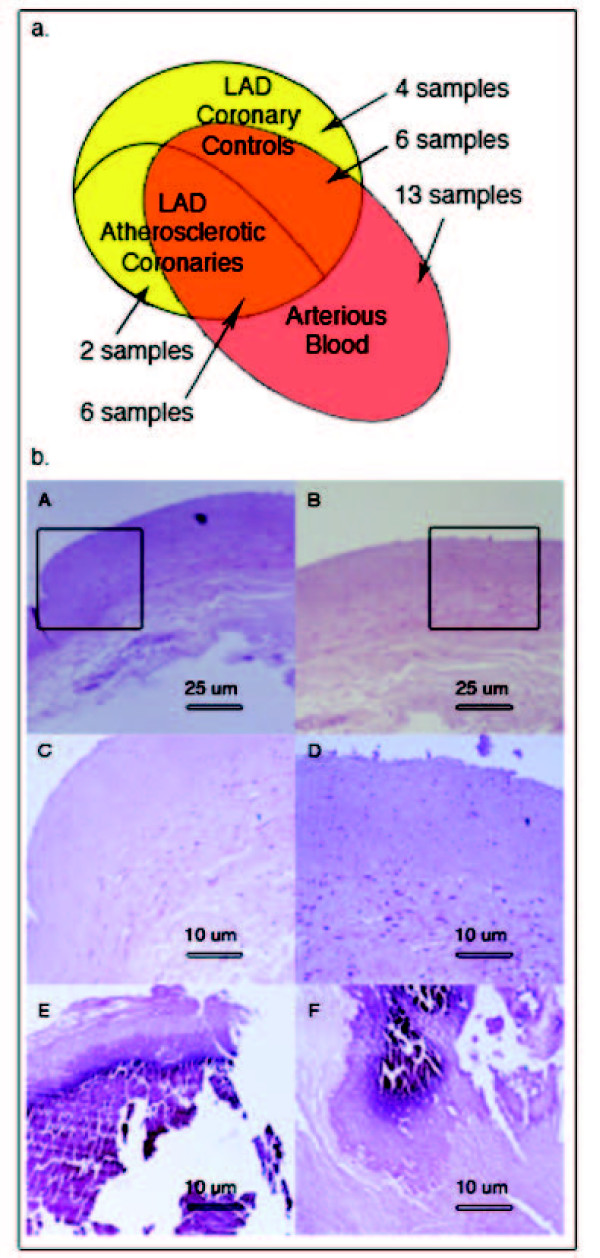
**a) Venn diagram representing the use of biological samples obtained from the cohort of patients recruited in our study**. Yellow indicates LAD coronary samples used for gene expression profiling and red indicates arterial blood samples subjected to blood cytokine analysis. Intersection between red and yellow areas defines patients from whom coronary and blood samples have been subjected to the respective analyses. **b) **Histological cross-sections of two control (A, B, and C, D enlargements) and two patient (E, F) coronary samples. A and B images show normal coronary walls while E and F, representative for all analyzed patients, evidence calcified plaque.

The traits of patients (PT) that have been analyzed in this study are reported in Table 1, and the use of samples obtained from them is summarized in Figure 1a. We produced gene expression profiles from 8 atherosclerotic LAD coronaries presenting at least 75% stenosis at angiography (PT 1–8), and compared with the profile of a pool made of 10 normal LAD coronaries (PT 9–18). Histochemical analysis on coronary samples shows calcified plaque in the patient samples, while no atherosclerotic tissue was found in controls (Figure 1b). According to the American Heart Association (AHA) classification[9] 71.4% of the patients presented plaques of type VII and 28.6% presented type VI plaques. Moreover, 76% of the coronary ischemic patients presented a hypertension status, according to the European Society of Hypertension/European Society of Cardiology[10] or were treated with antihypertensive drugs. Since atherosclerosis generally affects other vessels (aorta, carotid and iliac arteries), we used datasets available in public databases and compared 8 expression profiles of atherosclerotic carotids (PR 32–39) with the profiles of 6 normal coronary controls (PR 40–45). Also 75% of the patients with ischemic carotids presented a hypertension condition. We selected datasets derived from carotids with a stenosis that was comparable to LAD coronary samples. The general results obtained by this series of analysis are summarized in Table 2, whereas the detailed lists of differentially expressed genes in the atherosclerotic samples have been deposited and are available at GEO database (GSE11138). Comparing the datasets of the various samples, we obtained a group of common differentially expressed genes that we would like to define as human "atherogenes" (see Additional file 1, Table S2).

**Table 1:** Patient recruited (PT) and expression datasets retrieved (PR) for this study.

**Patient (PT)/Profile (PR)**	**Sex**	**Age (Years)**	**Tissue sample**	**Pathology/Lesion type**	**Co-morbidities**	**Pharmacological therapy**	**Plasma lipid/Stenosis/ICR**
PT1	M	50	LAD	Ischemic/VII	Hypothyroidism	D;AAP;AC;HYR	+/> 75%/5

PT2	F	68	LAD;AB	Ischemic/VII	None	D;AC	+/> 75%/4.6

PT3	M	66	LAD;AB	Ischemic/VII	Cholecystectomy	AAP;HYR	=/> 75%/5

PT4	M	64	LAD;AB	Ischemic/VII	None	D;HYR	+/> 75%/5.2

PT5	M	64	LAD;AB	Ischemic/VI	PAH	D;ST;AAP;HYR	+/> 75%/4.9

PT6	M	42	LAD;AB	Ischemic/VII	None	D;HYR	+/> 75%/5.3

PT7	M	53	LAD;AB	Ischemic/VII	Stone Kidney	D;HYR;AAP	+/> 75%/5.1

PT8	M	68	LAD	Ischemic/VI	None	D;HYR;AAP	+/> 75%/5.2

PT9	M	46	LAD;AB	DCM/ctrl	None	D;AC	+/-/4.6

PT10	M	48	LAD;AB	DCM/ctrl	Kidney pathology	D;AC	=/-/4.7

PT11	F	55	LAD;AB	DCM/ctrl	Thyroidectomy	D;AC;CDR	=/-/4.5

PT12	F	59	LAD;AB	DCM/ctrl	None	BB;AC;AAP	=/-/3.2

PT13	M	63	LAD;AB	DCM/ctrl	None	ACEH;D;AC	=/-/3.6

PT14	M	53	LAD;AB	DCM/ctrl	None	ACEH;D;AAP	+/-/3.8

PT15	M	62	LAD	DCM/ctrl	Gilbert's syndrome	AAP	=/-/3.5

PT16	M	61	LAD	DCM/ctrl	None	ACEH;ST	=/-/3.5

PT17	F	59	LAD	DCM/ctrl	None	ACEH;AC;AAP	=/-/4.3

PT18	F	41	LAD	DCM/ctrl	None	D	=/-/3.9

PT19	M	70	AB	Ischemic/VII	T2D	D;HYR;SI	+/> 75%/5

PT20	M	67	AB	Ischemic/VII	Mitral insufficiency	None	=/> 75%/4.3

PT21	F	82	AB	Ischemic/VII	HY	None	+/> 75%/4.6

PT22	M	63	AB	Ischemic/VI	None	None	=/> 75%/4.8

PT23	M	75	AB	Ischemic/VI	None	ACEH;HYR	+/> 75%/5.2

PT24	M	64	AB	Ischemic/VII	PD	HYR;D;LD	+/> 75%/5.1

PT25	M	75	AB	Ischemic/VII	T2D;HY	SI;HYR	+/> 75%/5.2

PT26	M	57	AB	Ischemic/VII	T2D;HY	SI;HYR	+/> 75%/5.5

PT27	M	66	AB	Ischemic/VII	HY	HYR	+/> 75%/5.2

PT28	M	69	AB	Ischemic/VII	HY	HYR	+/> 75%/5

PT29	M	51	AB	Ischemic/VI	None	None	=/> 75%/4.8

PT30	F	78	AB	Ischemic/VII	HY	HYR	+/> 75%/4.5

PT 31	M	75	AB	Ischemic/VI	None	None	=/> 75%/5.1

PR32*	M	67	Carotid	Ischemic	Acute bronchitis	NA	NA/75%/NA

PR33*	M	67	Carotid	Ischemic	Acute bronchitis	NA	NA/71%/NA

PR34*	F	63	Carotid	Ischemic	CAD;T2D;HY	NA	NA/75%/NA

PR35*	F	63	Carotid	Ischemic	CAD;T2D;HY	NA	NA/50%/NA

PR36*	F	59	Carotid	Ischemic	HY;HYCH	NA	NA/75%/NA

PR37*	F	59	Carotid	Ischemic	HY;HYCH	NA	NA/83%/NA

PR38*	F	71	Carotid	Ischemic	CAD;T2D;HY;HYCH;AD	NA	NA/70%/NA

PR39*	F	71	Carotid	Ischemic	CAD;T2D;HY;HYCH;AD	NA	NA/83%/NA

PR40†	NA	NA	Coronary	Normal	NA	NA	NA

PR41†	NA	NA	Coronary	Normal	NA	NA	NA

PR42†	NA	NA	Coronary	Normal	NA	NA	NA

PR43‡	M	NA	Coronary	Normal	NA	NA	NA

PR44‡	M	NA	Coronary	Normal	NA	NA	NA

PR45‡	M	NA	Coronary	Normal	NA	NA	NA

* PR of atherosclerotic carotids from E-MEXP-268, a study performed on carotid bilateral stenosis in which the two carotids (indicated by couple of consecutive numbers in the Table) of four independent patients have been analyzed. † PR of normal coronaries from GSE3526 (GSM80609, GSM80610, GSM80631). ‡ PR of normal coronaries from GSE7307 (GSM176115, GSM175820, GSM175821). ICR: index of cardiovascular risk; LAD: Left Anterior Descendent Coronary; AB: Arterial Blood; DCM/ctrl: Dilated Cardiomyopathy, used as pooled controls for microarray experiments; D: Diuretics; AAP: Anti-arrhythmics; AC: Anti-coagulants; HYR: Hypertension regulators; BB: Beta-blockers; ACEH: ACE inhibitors; ST: Statins; CDR: Cholesterol down-regulators; SI: Synthetic insulin; LD: Levodopa; PAH: Pulmonary hypertension; T2D: Type-2-diabetes; HY: Hypertension; HYCH: Hypercholesterolemia; PD: Parkinson's disease; AD: Artery disease; NA: not available.

**Table 2:** Summary of genes differentially expressed in atherosclerotic vessels.

**Tissue**	**% Up-regulated genes**	**% Down-regulated genes**	**Total number of deregulated genes**
LAD-specific	34.31	65.69	953

Carotid-specific	53.40	46.60	1736

Common	39.75	60.25	161

**Figure 2:**
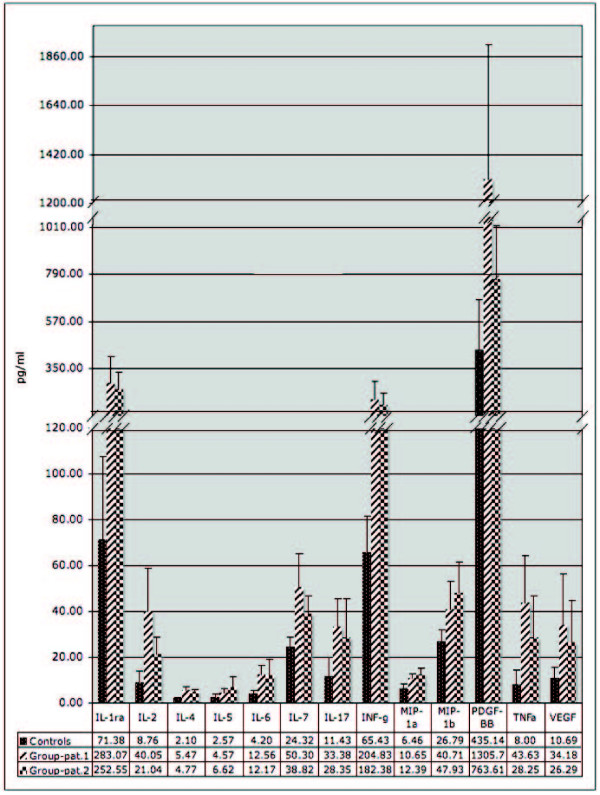
**Concentration of cytokines, chemokines and GFs differentially (p ≤ 0.05) expressed in arterial blood of patients**. Baseline indicates mean values concentration across patients or controls for each protein. RANTES concentration is not shown, since measured values in patient plasma were saturating the BioPlex instrument.

Finally, we measured cytokines and growth factors in the arterial blood of 6 patients, whose gene expression profiles had already been produced (PT 2–7), and of a group of 13 independent atherosclerotic patients with comparable stenosis of the coronaries (PT 19–31), to associate the atherosclerotic signals released in the blood to plaque presence. Data on blood circulating factors were related to those of 6 control individuals (PT 9–14). This study demonstrated significant alterations of 9 interleukins, 2 growth factors and 2 chemokines in all atherosclerotic samples (Figure 2).

To identify and characterize common molecular impairments in atherosclerotic samples, we studied the lists of atherogenes and blood-released factors and grouped them in functionally related classes and pathways that are described further. In many cases, the alteration of important elements of pathways has been validated by qRT-PCR.

### Extracellular matrix (ECM) and smooth muscle cells (SMC)

Many genes that are down-regulated in coronary and carotid plaques codify proteins of ECM (see Additional file 2, Figure S1). Collagen genes of type IV, VIII, XVIII, XIV and XXI are down-regulated in coronary and type XIV in carotid plaque. We also evidenced the down-regulation of a group of SMC-specific genes in both atherosclerotic vessels such as actin-alpha 2, PDLIM5 and Cysteine and glycine-rich protein 2 (CSRP2).

### Lipid homeostasis

**Figure 3:**
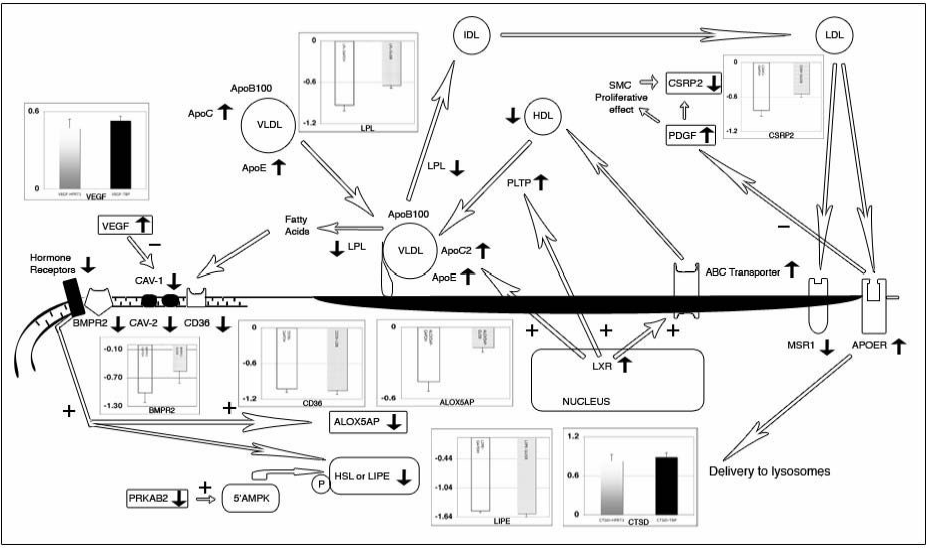
**Functional interaction map of differentially expressed genes and proteins in atherosclerotic samples that are involved in lipid homeostasis and caveolae system impairments**. Arrow direction near gene symbols is related to its expression relative to control. Histograms represent log_2 _expression value relative to control gene, obtained by qRT-PCR for the indicated factors. White color bar means that gene expression was referred to GAPDH reference gene, grey to GUSB, graduated grey to HPRT1 and black to TBP. On the left side of the map is drawn the caveolae pathway with CAV-1 and CAV-2 down-regulation connected to up-regulated VEGF. It is shown that associated hormone receptors and BMPR2 are down-regulated leading to ALOX5AP and LIPE down-regulation. In the central part of the map, the up-regulation of PLTP is connected to the loss of HDL concentration and to the accumulation of VLDL particles not hydrolyzed as a consequence of LPL down-regulation. The right part of the diagram shows that clearance of LDL by MSR1 is impaired, but not that mediated by APOER. This may influence PDGF transduction which is related to SMC de-differentiation. Finally, the map connects the up-regulations of APOE, APOC and ABC transporters to the increased transcription of LXR.

Interestingly, 5% of differentially expressed genes in LAD atherosclerotic coronaries are implicated in pathologies characterized by chronic hypertension. Since hypertension is often associated with dyslipidemias[11], we have clustered differentially expressed genes involved in lipid/cholesterol homeostasis and reconstructed the interaction map shown in Figure 3. We found up-regulation of ApoE gene in atherosclerotic coronaries and carotids, whereas ApoC2 and ApoC1 (components of the chylomicrons, VLDL and high-density lipoprotein (HDL)) are up-regulated in the carotids only. On entering circulation, triglycerides are hydrolyzed by lipoprotein lipase (LPL) and the resulting products are taken up by interaction of ApoE with LDL receptor or LRP. We detected down-regulation of LPL gene in atherosclerotic patients by microarray and confirmed it by qRT-PCR. Fatty acids produced by LPL activity are removed from circulation by CD36[12], but we identified down-regulation of this gene both by microarrays and qRT-PCR. MSR1, another scavenger receptor, was down-regulated in LAD coronaries. Low abundance of atheroprotective HDL, in response to up-regulated phospholipid transfer protein (PLTP) may be balanced by a major uptake of the atherogenic LDL. APOER gene is up-regulated and also ABC transporter genes are deregulated. Finally, we evidenced the up-regulation of the liver × receptor (LXR).

### Caveolae system

We evidenced the down-regulation of the Cav-1 and the related Cav-2 gene in coronary samples. Cav-2 gene down-regulation has also been confirmed in the atherosclerotic carotids. It is known that angiogenic factors such as VEGF induce down-regulation of caveolin-1 and, as matter of fact, VEGF was up-regulated in both coronary and carotid plaque. To confirm this result we measured VEGF mRNA by qRT-PCR evidencing 1.52 fold over-expression in patients versus controls. Moreover, the VEGF protein was significantly (p < 0.05) increased in the plasma of all atherosclerotic patients (Figure 2 and 3).

### Steroid hormones and bone morphogenetic protein type II receptor (BMPR2)

The importance of pathways related to steroids in atherosclerosis is substantiated by a variety of data in our study. The estrogen receptor ESR1, the progesterone receptor PGRMC1 and the glucocorticoid receptor (GR) NR3C1 genes are down-regulated in the atherosclerotic coronaries and NR3C1 also in carotids. In addition, we evidenced the down-regulation of SUMO1 both in coronary and carotid samples, and it is known that GR are activated by sumoylation[13]. In this context, other important data are the down-regulation of hormone sensitive lipase gene (HSL or LIPE) and its interacting protein adipocyte fatty acid-binding (AFABP) in both coronary and carotid plaques. This has been confirmed by qRT-PCR evidencing 2.85 fold negative regulation of HSL in atherosclerotic vessels. Although present, HSL may also be inactive because the positive regulator of the 5'AMPK protein PRKAB2 is under-expressed.

Since Donn R et al.[14] demonstrated the functional interaction of glucocorticoid receptor with BMPR2, we studied its expression by microarray and qRT-PCR evidencing the down-regulation of this gene in both coronary and carotid plaques (Figure 3). Actually, the whole pathway controlled by BMPR2 is altered. For instance, SMAD5 and ID4 genes, involved in BMPR2 signal transduction, are down-regulated. Interleukin-6 (IL-6) and its activation of STAT3 signaling contribute to the pulmonary arterial hypertension (PAH) related to BMPR2 down-regulation[15]. We found increased level of IL-6 mRNA in coronary samples and of IL-6 protein in plasma of atherosclerotic patients (Figure 2). IL-6 is involved in the activation of JAK/STAT signaling pathway, which seems to be critical for atherosclerotic plaque development[16]. This pathway is generally enhanced in coronary and carotid plaques as demonstrated by the up-regulation of STAT1, Interleukin 10 receptor and IFN-alpha-responsive transcription factor subunit (ISGF3G). Also IL-2, -4, -5, -7 and INF-gamma that are known activator of this pathway, are more abundant in patients than in controls (Figure 2). In addition, other genes belonging to this pathway are specifically up-regulated (TYK2, Sprouty 1 an inhibitor of the Ras/MAPK pathway and SOCS1) or down-regulated (PI3K, SOCS4 and leptin) in coronary samples.

### Networks of deregulated genes/proteins in atherosclerotic samples

**Figure 4:**
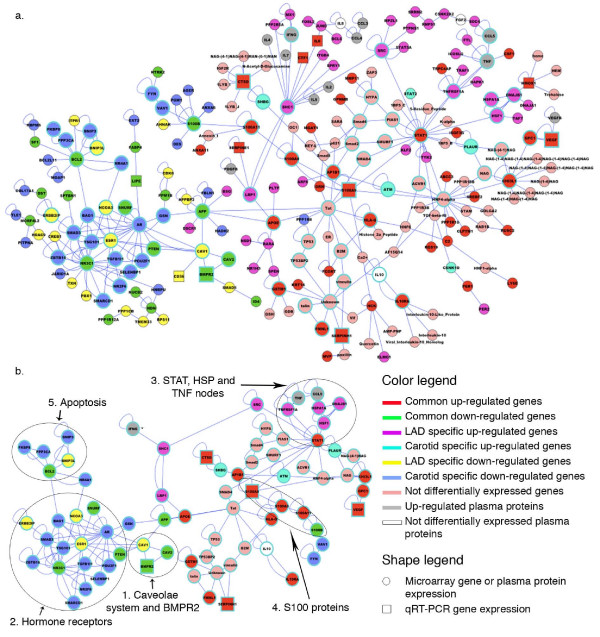
**a) Interaction network of genes/proteins differentially expressed in atherosclerotic arteries**. Interaction are described in the Additional file 1, Table S3; **b) **enlargement of the highly interconnected nodes of the interaction map showing the genes involved in the caveolae and steroid hormone receptor pathways. See text for discussion.

The functional analysis of atherogenes resulted in the construction of a network of molecular interactions that is presented in Figure 4a. The functional classification of genes in the network is presented in the additional Figure S2 (see Additional file 2, Figure S2). This highly interconnected network could be subdivided in two large regions: the first contains the down-regulated genes (left of Figure 4a) and the second the up-regulated genes (right of Figure 4a). Among the up-regulated genes, the functional categories of the cytokines (p-value 5.36 E^-15^) and chemokines (p-value 2.32 E^-4^) are over-represented. This is in accordance with results obtained by blood analysis that confirm the presence of JAK/STAT induction factors and regulators for the inflammatory process. The steroid hormone receptor activity (p-value 6.9 E^-8^) and related functions, are among the down-regulated genes. Analyzing at a finer level the two regions of the map, it became clear that there are five central nodes of interactions, enlarged in Figure 4b. The first is composed by genes of the caveolae system connected to BMPR2, and the second node is composed of hormone receptors. A third node is centered on STAT1 that is related to up-regulated members of HSP40 family, interacting with HSP70, HSP40, HO-1 and HSP47. The fourth node is composed by S100A8 and S100A9 genes whose proteins are involved in inflammatory processes. Finally, the fifth node points to the anti apoptotic gene BCL2.

## Discussion

As far as major factors influencing atherosclerotic traits are concerned, our study was based on a homogeneous cohort of patients. Patients and controls were matched for age, as ageing influences the vascular substrate and the development and progression of atherosclerosis. LAD patients were 59 years old ± 9.7, while controls were 54.7 years ± 7.5. Datasets of gene expression in carotid plaques were also obtained from patients with similar age. Moreover, all atherosclerotic patients had comparable stenosis. Due to this homogeneity of experimental sources, the differentially expressed genes that we have found should be considered as generally involved in the molecular events establishing and maintaining atherosclerosis.

The atherogenes network described in Figure 4, shows the importance of both the inflammatory response and caveolae system, the first controlled by central node STAT1 (inducing also the over-expression of the heat shock proteins HSP47[17] and HO-1), the second related to steroid hormone receptors.

STATs are cytosolic proteins that, upon activation, translocate in to the nucleus to activate target genes. Originally, this pathway was found to be activated by INFs, but a number of cytokines, growth factors, and hormonal factors were successively found as activators of JAK and/or STAT proteins (see Additional file 2, Figure S3). In particular, INF-g activates STAT1 one of the central nodes of our atherogene network (Figure 4) and this is in accordance with the high level of the interferon protein found in patients plasma (Figure 2). It is noteworthy that the anti-inflammatory cytokine IL-10, which is not altered in the patient blood, also activates JAK/STAT protein[18]. Its receptor results among the up-regulated atherogenes (Figure 4), confirming the importance of this molecule in the response to the pathological status to translate signals by IL-10 molecule. These results confirm previous data obtained in the atherosclerotic tissues but allow in addition the link between gene expression and plasma proteins. The importance of the STAT, INFs and IL-10 signals is strengthened by our results, since we were able to detected it in atherosclerotic plaques of different vessels in a series of 25 patients and 29 samples (Table 1).

Estrogen receptors (ESR) 1 and 2, expressed in normal and atherosclerotic arteries, mediate the protective action of estrogen to artery wall. ESR1 genotype is a predictor of complex lesions, of coronary thrombosis, and has a role in rising HDL cholesterol level[19]. Glucocorticoids and their receptor (NR3C1) are also involved in atheroprotective effect. Our data suggest that the protective effect is impaired by GR down-regulation in atherosclerotic plaques from coronaries and carotid (Figure 3 and 4b).

The activation of HSL is compromised by the down-regulation of both the hormone receptors and HSL activator PRKAB2 (Figure 3). Decrease of adipocyte HSL activity has been demonstrated in familial combined hyperlipidemia, characterized by excessive concentrations of serum-free fatty acids[20]. All patients showed an elevated cholesterol concentration in the blood with index of cardiovascular risk (total cholesterol concentration/HDL) ≥ 5 for males and 4.5 for females.

Glucocorticoid receptor interacts with BMPR2[14], a component of the TGFBR superfamily. BMPR2 is located within caveolae of endothelial cell membranes suggesting potential dynamic regulatory structural relationships[21] lost in plaque (Figure 3 and 4b). BMPR2 gene defect is involved in the development of pulmonary arterial hypertension (PAH). PAH is characterized by the reduction of the lumen of pulmonary arteries and serum IL-6 increase. Additionally, IL-6 level was dramatically increased in transgenic mice expressing a mutant BMPR2 which spontaneously developed PAH[22]. IL-6 is a cytokine with a wide variety of biological functions. We focalized our attention on its involvement in JAK/STAT pathway because it appeared significantly compromised in both coronary and carotid plaques (see Additional file 2, Figure S3). It is considered a stress responsive signaling cascade, involved in down-regulation of matrix genes[23] that could promote plaque rupture. In fact, a reduced production of collagen and its uncontrolled degradation may affect the stability of the vessel wall especially in atherosclerotic plaques by making them prone to aneurysm and rupture. Type IV, XV, XVIII and XIX collagens are structural elements associated with the vascular basement membranes. Each individual SMC is surrounded by a basement membrane. Thus, all membrane-associated collagens are probably involved in the regulation of intercommunication between vessel lumen and wall or between individual SMCs in the intimal and medial layers. This intercommunication appears to loosen in the coronaries and carotids, and this probably leads to de-differentiation of SMC highlighted by down-regulation of CSRP2. This gene is highly expressed in aorta and is drastically down-regulated in response to PDGF-BB (Figure 3) or cell injury promoting SMC proliferation and de-differentiation[24]. A dominant-negative CSRP2 mutant blocked proepicardial cells from differentiating into SMC[25]. The possible de-differentiation of SMC is also supported by the down-regulation of ACTA2 and PDLIM5 genes in both carotids and coronaries. Proliferative effect of PDGF on SMC could be inhibited by APOER which presents both a protective role in the vasculature and an atherogenic function[26] (Figure 3). APOER up-regulation could balance de-differentiative effects induced by PDGF on SMC, but could allow SMC transformation to foam cells and impairment of vessel structure. This correlation was already evidenced by the work of King Y.J. et al.[6] but, in addition to this, we have correlated the extracellular matrix with the expression of two important LIM proteins involved in the development of muscle cells: CSRP2 and PDLIM5. These genes could become important targets to modulate the effects of the atherosclerotic lesion at its initial stage.

HDL mediate cholesterol clearance from foam cells but, recently, raising HDL as a treatment for cardiovascular disease has been disputed because of the failure of the torcetrapib therapy[27]. Despite this negative data, HDL-based therapies such as reconstituted HDL, phospholipids and APOA1_Milano _have shown some promising results focusing the importance of lipids homeostasis in atherosclerosis. LXR has a central role in the lipid homeostasis regulating ABC transporter, PLTP and ApoE/C-I/C-IV-CII gene cluster expression (Figure 3). Coronary and carotid gene expression profiles evidenced the up-regulation of the ABCC3 gene, a new member of the ATP-binding-cassette family associated with atherosclerosis in mouse[28]. ABC transporter regulates efflux of cholesterol and phospholipids from peripheral tissues and macrophages mediating reverse cholesterol transport (RCT). RCT is considered to be the primary mechanism through which HDL protects against atherosclerosis, but its production is limited in LAD patients by LPL down-regulation and PLTP up-regulation. Moreover, the index of cardiovascular risk was ≥ 5 and 4.5 for males and females respectively indicating low HDL in patient blood. PLTP over-expression appears to be connected to a decrease of HDL and to an increase of ApoE that prevents its inactivation. PLTP has been already identified like one of the "candidate genes" for atherosclerosis[29,30] and this is confirmed by our meta-analysis. On the other hand LPL is an enzyme present in capillary surface, and lack of it causes familial hyperlipoproteinemia type I, characterized by massive hypertriglyceridemia. The impairment of the lipolysis process could support low HDL production by this pathway.

Fatty acids and lipoproteins are removed from circulation by scavenger receptors. CD36 and MSR1 genes are down-regulated in atherosclerotic vessels and this could contribute to the impairment of clearance process and cause the elevated values in the analyzed patients (Table 1). CD36 is a scavenger receptor for modified forms of LDL and its loss leads to a pro-atherogenic lipid profile in mice[31] while MSR1 scavenger increases clearance of modified lipoproteins, protecting the arterial wall against the pro-atherogenic action of LP[32]. The down-regulation of CD36 and MSR1 could result in a increasing of atherosclerotic lesion in vessel as it was demonstrated in studies on SR-BI^-/- ^ApoE^-/- ^mouse model with microdeletions in scavenger genes[33]. Our data are coherent with the dyslipidemia of atherosclerotic patients (Figure 3) that could be associated with the subsequent development of hypertension causing damage to the inner walls of arteries. These data could apparently appear in contrast with the supposed pro-atherogenic function of CD36. However this protein could play very different roles due to the variety of functions that have been associated to it. In fact, it is involved also in the regulation of the angiogenesis in association with thrombospondin (TSP)[34]. Angiogenesis is the body response to ischemia and vascular injury and is inhibited by the interaction of CD36 with TSP-1. CD36-TSP-1 interaction is also important in the maintenance of an anti-inflammatory milieu in the uptake of apoptotic cells as a homeostatic process, and during wound resolution. The down regulation of CD36 may be also important to maintain the ability of producing novel vessels important for the plaque maintaining supplying nutrition to grow[35].

CD36 and SR are related to Caveolin-1 (Cav-1)[36] which is a cholesterol-binding protein transporting cholesterol from the endoplasmic reticulum to the plasma membrane. Before this work, there was limited evidence of altered Cav-1 levels in human atherosclerotic lesions[37]. Loss of caveolin-1 in ApoE^-/- ^mouse resulted in a pro-atherogenic lipid profile, similar to that seen in CD36^-/- ^mice bread to an ApoE background[31]. Absence of Cav-1 and caveolae in endothelial cell (EC) lead to increased eNOS activity and NO release, resulting in reduced vascular tone[38]. Furthermore, Caveolin-1 is a negative regulator of EC proliferation but promotes cellular differentiation[38]. However, angiogenic factors such as VEGF have been shown to induce down-regulation of caveolin-1, which was suggested to be important for the mitogenic effects of growth factors in EC[38,39]. Caveolin genes down-regulation therefore could lead to decreased inhibition of EC proliferation and to a negative effect on SMC differentiation. Dyslipidemia evidenced in Cav-1^-/- ^and CD36^-/- ^mice is congruent once more with our hypothesis on involvement of Caveolin genes in the control of cholesterol homeostasis.

In summary, the analysis of the atherogenes network that we have outlined shows that the atherosclerotic phenotype of arterial vessels is maintained by altered pathways of hormone receptors, lipid homeostasis and caveolae system that are highly interconnected through specific nodal genes. This is a novel interconnection evidenced in the atherosclerotic plaque that has been confirmed in different patients (different genetic background) and different atherosclerotic sites. Before our work, there were no evidences for the relationship between caveolae, BMPR2 and hormone receptor genes that are in turn related, in our network, to genes involved in the apoptotic process (Figure 4). The involvement of the caveolae system in human lipid homeostasis and atherosclerosis has been confirmed. This is a central node linking the network of down regulated genes (left in Figure 4) to the up regulated one (right in Figure 4). This connection is sustained by APP (amyloid beta precursor protein) and APOE, previously discussed. Perhaps it should be considered the possibility that amyloid-β (Aβ) might be involved in vascular pathology. In fact, APP is involved in blood clotting[40] and Aβ drains from the brain along the walls of the microvasculature[41]. Down regulation of APP gene could be involved in thrombosis effect[40] related to the advanced status and unstable condition of the plaques studied in this work (Figure 1).

The portion of the network with over expressed genes is characterized by the inflammatory response regulated by JAK/STAT pathway and is integrated by the data on plasma proteins involved in this process. These data confirm the known involvement of JAK/STAT pathway in atherosclerosis, but our study has validated for the first time the common deregulation of these genes in different atherosclerotic sites and correlated to the hormone receptor, caveolae system and APP connected to the instability of the plaque.

## Conclusion

Atherosclerosis affects aorta, coronary, carotid, and iliac arteries most frequently than any other body vessel. Our study aimed to the description of common molecular pathways sustaining this process, integrating data obtained by gene expression profiling of human atherosclerotic vessels and by parallel measuring of blood circulating factors.

We were able to define a network of functionally connected genes that are commonly altered in atherosclerotic vessels (human "atherogenes"). These pathways and genes could be useful information for the development of new drugs against imbalance of cholesterol homeostasis and hypertension. Interesting potential targets could be PLTP and BMPR2 genes.

## Methods

### Patient characterization and tissue sampling

For this study 31 patients were recruited among those subjected to heart transplantation for ischemic cardiomyopathy (ICM) or dilated cardiomyopathy (DCM) at the Cardiovascular Surgical Unit of the University of Verona Medical School. On the basis of clinical diagnosis and angiography, patients were divided in atherosclerotic (with 75% stenosis of one coronary artery at least), and non-atherosclerotic controls (Table 1).

Immediately after cardiotomy, left anterior descendent (LAD) coronaries were dissected from diseased hearts, cleaned from fatty and cardiac muscle tissues, and stored in RNAlater Solution (Ambion). 5-μm-thick serial sections of paraffin-included coronary fragments were used for histology. All atherosclerotic lesions were classified as type VI or VII according to the criteria proposed by the American Heart Association[9]. Typically, type VI lesions were characterized by one or more surface defect, hematoma and thrombosis, while calcification prevailed in type VII plaques.

Arterial blood from atherosclerotic and non-atherosclerotic individuals was collected, before anti-coagulant administration, in BD Vacutainer CPT cell preparation tubes, and processed according to manufacturer's directions. We recovered blood in the vicinity of the atherosclerotic plaque in order to evaluate factors released from the plaque and cells in the surrounding area. Blood was recovered from the pulmonary artery of patients after anesthesia and before administration of anti-coagulants.

The ethical committee approved the study and written informed consent was obtained from all the participants after exhaustive information on the study.

### Coronary RNA extraction and gene expression experiments

Visible plaques, adjacent to the segment utilized for histological analysis, were dissected from the entire coronary fragment and used for total RNA extraction with TRIzol (Invitrogen). RNA was prepared also from 10 non-atherosclerotic coronaries, pooled and utilized as a common control sample. Control RNA pool and plaque RNAs (1 μg) were linearly amplified with MessageAmp™ aRNA amplification kit (Ambion), labeled with Cy3 and Cy5 dye (Amersham) and competitively hybridized to oligonucleotide microarrays platforms (GEO ID: GPL6647). For each sample we performed two microarray experiments at least. Microarrays were read with the ScanArray LITE confocal laser scanner (PerkinElmer). Each slide was subjected to three consecutive scans at low, medium and high settings of laser and photomultiplier[42]. We therefore produced six 16-bit images per microarray, grouped in low, intermediate and high intensity scans. Images were quantified with ScanArray Express software (PerkinElmer). Data were normalized using the total and lowess methods, implemented in the MIDAW tool[43] and submitted to GEO database (GSE11138). Data filtering was made as outlined in the Additional file 3. For each group of images obtained with different laser power, the genes with expression values in all experiments were analyzed by SAM program[44] implemented in the TMEV software[45]. SAM identifies statistically significant changes in gene expression by applying a modified t-test and controlling false discovery through a permutation technique. We performed 100 rounds of such permutations. Finally, the lists of differentially expressed genes presenting false discovery rate of 0 were integrated[42].

### Meta-analysis

To compare our expression data with published expression profiles, we applied a meta-analysis approach to expression datasets of 8 carotid samples taken from Array Express database (E-MEXP-268) and 6 coronary controls retrieved from Gene Expression Omnibus (GSE3526, GSE7307), using row data only[46]. Normal coronary profiles were used as common reference for carotid plaque expression data, since no important differences emerged from the comparison between normal carotid and normal coronary expression profiles (data not shown). Moreover, since the characteristics of peripheral arteries, like thickness of intima-media layers of carotid wall, are used as surrogate markers for coronary atherosclerosis, we decided to compare directly gene expression of these two vessels. However, since it is known that atherosclerosis show some rate of artery-dependent patterns[47], it should be clarified that the comparison of diseased carotids to normal coronaries could result in some grade of under- or overestimation of differences in gene expression. Briefly, data have been normalized using the invariant probe set normalization method[48], implemented in d-chip software, and matched for the common probe set between the different platforms used in the experiments. Differentially expressed genes were calculated from normalized values by applying the fold change and t-test methods.

The lists of genes differentially expressed in atherosclerotic coronaries and carotids were compared using the identification of Entrez Gene database and matching entries were classified as "atherogenes" (see the Results). Coronary specific, carotid specific and common differentially expressed genes have been functionally classified using Gene Ontology criteria as implemented in DAVID[49] and used to build networks of functionally correlated genes/protein by Cytoscape[50]. Cytoscape is an open source bioinformatic platform for visualizing molecular interaction networks and biological pathways. To retrieve interactions between differentially expressed genes and to construct the larger general interaction network (Figure 4a), we used the Biomolecular Interaction Network Database (BIND) , that is a collection of records documenting protein interaction, molecular complexes and pathways. We also made use of the Biological General Repository for Interaction Datasets (BioGRID) database [51] developed at the University of Toronto (Canada) to house and distribute collections of proteins and genetic interactions from major model organisms. This larger network was used to identify high interconnected nodes with the MCODE[52] Cytoscape plug-in. MCODE is an algorithm that allows the finding of clusters in interaction networks to evidence protein complexes or related pathways. Parameters used for MCODE were: Score 1.80, Nodes 76 and 170 Edges. The MCODE defines score as the product of the complex sub-graph density and the number of vertices in the complex sub-graph. With this process MCODE assigns higher values to large node and dense complexes. The inferred sub-network displayed in Figure 4b was that classified with the highest score.

Functional classification of genes connected in the larger network (Figure 4a) was done according to Gene Ontology (GO) using the Cytoscape plug-in BINGO[53]. This is a bioinformatic tool able to determine which GO categories are statistically overrepresented in a set of genes or a sub-graph of a biological network. We used a hypergeometric test and the Benjamini and Hochberg False Discovery rate (FDR) correction with 0.05 level of significance. The results are plotted in the additional Figure S2 (see Additional file 2, Figure S2).

### Quantitative real-time PCR

Equal aliquots of aRNA from each sample were mixed and 400 ng of this pool were used for first strand cDNA synthesis using Superscript II (Invitrogen). Four independent reactions were carried out, pooled and used for qRT-PCR with SYBR green. Each qRT-PCR was performed in triplicate using the 7500 Real Time PCR System (Applied Biosystems), and analyzed according the Pfaffl method[54]. Glyceraldehyde-3-phosphate dehydrogenase (GAPDH), glucuronidase beta (GUSB), TATA box binding protein (TBP) and hypoxanthine phosphoribosyltransferase 1 (HPRT1) were used as reference transcripts. Sequences of the primers used for qRT-PCR of various mRNA are reported in the additional Table S1 (see Additional file 1, Table S1).

### Blood plasma analysis

Plasma was used to analyze cytokine and growth factors concentration using the Bioplex instrumentation (BioRad). We used the human cytokine 27-plex panel according to the manufacturer's specificity.

## Competing interests

Authors declare no competing financial interests and relationships with pharmaceutical companies, biomedical device manufacturers, or other corporations whose products or services are related to the subject matter of the article.

## Authors' contributions

SC, MB. Carried out the Bioplex immunoassays, performed microarray and qRT-PCR experiments and statistical analysis and write the manuscript. CP, ET, AP, MI, PL. Participated in the design of the study and involved in drafting the manuscript. MI. Performed histological analysis. GF, AM, PFP, GL. Conceived of the study, and participated in its design and coordination. PFP, GL. Revised the manuscript critically for important intellectual content.

The Authors had full access to the data and take responsibility for its integrity. All Authors have read and agreed to the manuscript as written.

## Supplementary Material

Additional file 1 **Additional Tables. **Tables included in this file include primer sequences used for the qRT-PCR, the list of common differentially expressed genes in coronary and carotid atherosclerotic samples (human "atherogenes") and the list of interacting elements for the visualization of the network in Figure 4a. Click here for file

Additional file 2 **Additional Figures.** Figures included in this file include the functional classification of genes of the extra-cellular matrix, the functional classification of the atherogenes connected in the network of Figure 4a and the representation of the JAK/STAT pathway. Click here for file

Additional file 3 **Expanded Methods. The data provided represent the expanded methods of the paper.** Click here for file
