# Supplementary material for: Reconstruction and functional analysis of altered molecular pathways in human atherosclerotic arteries
Source: BMC Genomics. 2009 Jan 9;10:13. doi: 10.1186/1471-2164-10-13 (PMC2654039; doi:10.1186/1471-2164-10-13)
Supplement: Additional file 1 — Additional Tables. Tables included in this file include primer sequences used for the qRT-PCR, the list of common differentially expressed genes in coronary and carotid atherosclerotic samples (human "atherogenes") and the list of interacting elements for the visualization of the network in Figure 4a. [file 1471-2164-10-13-S1.doc]

**Additional Tables**

**Additional Table S1**

**Table S1**. Sequences of the primers used for transcript-specific quantitative real-time PCR assays.

| **Primers Sequence 5’ – 3’** | **Gene** |
| --- | --- |
| GAAGATGTCGGAGCCCATAG | Left LIPE (HSL) |
| GGTCAGGTTCTTGAGGGAATC | Right LIPE (HSL) |
| TGCTTGACTTCTGTGGCTTTT | Left BMPR2 |
| GCATGACAGCTACGCATCTC | Right BMPR2 |
| CAGCTCGGATTCAACTACCTTAG | Left SERPINE1 |
| TTACATCCATCTTTGTGCCCTA | Right SERPINE1 |
| GCACAGAAACAGAGGAGAGTCC | Left CTSD |
| GAGTGTGTGGGTGTGTGTGG | Right CTSD |
| CCTGTCCCTGCACACCTC | Left CTF1 |
| GTGTTGCTTGTTTTGTCTCCAC | Right CTF1 |
| CTGACCCAACCACAAATGC | Left IL6 |
| GTCATGTCCTGCAGCCACT | Right IL6 |
| GCTGGAACGCAACATAGAGA | Left S100A9 (CALGRANULIN) |
| CTTTGAATTCCCCCTGGTTC | Right S100A9 (CALGRANULIN) |
| TGACAAGATGCGAGACGAGT | Left SERPINH1 (HSP47) |
| TGTCTCAGGAGCCTTTGGAT | Right SERPINH1 (HSP47) |
| CCCTTCAGCATCCTCAGTTC | Left HMOX1 (HO-1) |
| AGACAGCTGCCACATTAGGG | Right HMOX1 (HO-1) |
| TGTGTGTGTGTGAGTGGTTGA | Left VEGF |
| TCTCTGTGCCTCGGGAAG | Right VEGF |
| CGGAAGTGACTTTGAAAACTACATAA | Left ALOX5AP |
| GGAATGAGAAGTAGAGGGGAGAT | Right ALOX5AP |
| CCCAGTAGCTGCCCTATTCA | Left CD36 |
| ATTTGGTGCACATGAAATGC | Right CD36 |
| CTGACTGAAAAAGAAGGTGAAATCT | Left CSRP2 |
| TTACTGGGCATGAACAAGAGC | Right CSRP2 |
| TGTATGTGTCTTTATCAGTGATGGTC | Left LPL |
| TGTTTTGTTAAAGCCCATTTCA | Right LPL |
| ACATCATCCCTGCCTCTACTG | Left GAPDH |
| ACCACCTGGTGCTCAGTGTA | Right GAPDH |
| AACAGTCACCGACGAGAGTGCT | Left GUSB |
| GGTTTCATTGGCAATCTTCCAG | Right GUSB |
| GAACCACGGCACTGATTTTC | Left TBP |
| CCCCACCATGTTCTGAATCT | Right TBP |
| GACCAGTCAACAGGGGACAT | Left HPRT1 |
| AACACTTCGTGGGGTCCTTTTC | Right HPRT1 |

**Additional Table S2**

**Table S2.** List of common differentially expressed genes in coronary and carotid atherosclerotic samples (human “atherogenes”). LL_ID: LocusLink ID. For each gene is reported the Gene Ontology description for the Cellular Component (CC), Biological Process (BP) and Molecular Function (MF). UP_SEQ_FEATURES: Functional Categories based on UniProt database. Informations are retrieved from DAVID database (http://david.abcc.ncifcrf.gov/home.jsp).

| **Up-regulated** | | | | | |
| --- | --- | --- | --- | --- | --- |
| **LL_ID** | **Gene Name** | **GOTERM_CC_ALL** | **GOTERM_BP_ALL** | **GOTERM_MF_ALL** | **UP_SEQ_FEATURE** |
| 6282 | S100 CALCIUM BINDING PROTEIN A11 (CALGIZZARIN) | cell, cytoplasm, intracellular, intracellular membrane-bound organelle, intracellular organelle, membrane-bound organelle, nucleus, organelle, | DNA metabolism, DNA replication, DNA-dependent DNA replication, biopolymer metabolism, cell communication, cell proliferation, cellular metabolism, cellular physiological process, cellular process, macromolecule metabolism, metabolism, negative regulation of DNA metabolism, negative regulation of DNA replication, negative regulation of biological process, negative regulation of cell proliferation, negative regulation of cellular metabolism, negative regulation of cellular physiological process, negative regulation of cellular process, negative regulation of metabolism, negative regulation of nucleobase, nucleoside, nucleotide and nucleic acid metabolism, negative regulation of physiological process, nucleobase, nucleoside, nucleotide and nucleic acid metabolism, physiological process, primary metabolism, regulation of DNA metabolism, regulation of DNA replication, regulation of biological process, regulation of cell proliferation, regulation of cellular metabolism, regulation of cellular physiological process | binding, calcium ion binding, cation binding, ion binding, metal ion binding, | calcium-binding region:1; low affinity, calcium-binding region:2; high affinity, domain:EF-hand 1, domain:EF-hand 2, |
| 11119 | BUTYROPHILIN, SUBFAMILY 3, MEMBER A1 | cell, integral to membrane, intrinsic to membrane, membrane, | lipid metabolism, metabolism, physiological process, primary metabolism, | Null | Null |
| 5187 | PERIOD HOMOLOG 1 (DROSOPHILA) | cell, intracellular, intracellular membrane-bound organelle, intracellular organelle, membrane-bound organelle, nucleus, organelle, | cell communication, cellular metabolism, cellular physiological process, cellular process, circadian rhythm, entrainment of circadian clock, metabolism, negative regulation of biological process, negative regulation of cellular metabolism, negative regulation of cellular physiological process, negative regulation of cellular process, negative regulation of metabolism, negative regulation of nucleobase, nucleoside, nucleotide and nucleic acid metabolism, negative regulation of physiological process, negative regulation of transcription, nucleobase, nucleoside, nucleotide and nucleic acid metabolism, physiological process, primary metabolism, regulation of biological process, regulation of cellular metabolism, regulation of cellular physiological process, regulation of cellular process, regulation of metabolism, regulation of nucleobase, nucleoside, nucleotide and nucleic acid metabolism, regulation of physiological process, regulation of transcription, regulation of transcription, DNA-dependent, rhythmic proce | binding, nucleic acid binding, protein binding, signal transducer activity, | domain:PAS 1, domain:PAS 2, |
| 3135 | HLA-G HISTOCOMPATIBILITY ANTIGEN, CLASS I, G | MHC class I protein complex, MHC protein complex, cell, cellular component unknown, immunological synapse, integral to membrane, intrinsic to membrane, membrane, plasma membrane, protein complex, | antigen presentation, antigen presentation, endogenous antigen, antigen processing, antigen processing, endogenous antigen via MHC class I, cellular defense response, cellular physiological process, cellular process, defense response, detection of biotic stimulus, detection of external stimulus, detection of pest, pathogen or parasite, detection of stimulus, immune response, organismal physiological process, physiological process, response to biotic stimulus, response to external stimulus, response to other organism, response to pest, pathogen or parasite, response to stimulus, response to stress, response to wounding, | MHC class I receptor activity, receptor activity, signal transducer activity, transmembrane receptor activity, | disulfide bond, domain:Ig-like C1-type, glycosylation site:N-linked (GlcNAc...), signal peptide, transmembrane region, |
| 6810 | SYNTAXIN 4A (PLACENTAL) | cell, integral to membrane, intrinsic to membrane, membrane, | cell organization and biogenesis, cellular localization, cellular physiological process, cellular process, establishment of cellular localization, establishment of localization, establishment of protein localization, intracellular protein transport, intracellular transport, localization, neurotransmitter transport, physiological process, protein localization, protein transport, transport, | binding, protein binding, protein transporter activity, transporter activity, | domain:t-SNARE coiled-coil homology, transmembrane region, |
| 6772 | SIGNAL TRANSDUCER AND ACTIVATOR OF TRANSCRIPTION 1, 91KDA | cell, cytoplasm, intracellular, intracellular membrane-bound organelle, intracellular organelle, membrane-bound organelle, nucleus, organelle, | I-kappaB kinase/NF-kappaB cascade, JAK-STAT cascade, STAT protein nuclear translocation, apoptosis, apoptotic program, biopolymer metabolism, biopolymer modification, caspase activation, cell communication, cell cycle, cell death, cell organization and biogenesis, cellular localization, cellular macromolecule metabolism, cellular metabolism, cellular physiological process, cellular process, cellular protein metabolism, death, establishment of cellular localization, establishment of localization, establishment of protein localization, intracellular protein transport, intracellular signaling cascade, intracellular transport, localization, macromolecule metabolism, metabolism, nuclear import, nuclear transport, nucleobase, nucleoside, nucleotide and nucleic acid metabolism, nucleocytoplasmic transport, peptidyl-amino acid modification, peptidyl-tyrosine modification, peptidyl-tyrosine phosphorylation, phosphate metabolism, phosphorus metabolism, phosphorylation, physiological process, positive regulation of boil | DNA binding, binding, calcium ion binding, cation binding, ion binding, metal ion binding, nucleic acid binding, protein binding, signal transducer activity, transcription factor activity, transcription regulator activity, | domain:SH2, mutagenesis site, splice variant, |
| 6574 | SOLUTE CARRIER FAMILY 20 (PHOSPHATE TRANSPORTER), MEMBER 1 | cell, integral to membrane, integral to plasma membrane, intrinsic to membrane, intrinsic to plasma membrane, membrane, plasma membrane, | I-kappaB kinase/NF-kappaB cascade, anion transport, cell communication, cellular metabolism, cellular physiological process, cellular process, establishment of localization, inorganic anion transport, intracellular signaling cascade, ion transport, localization, metabolism, phosphate metabolism, phosphate transport, phosphorus metabolism, physiological process, positive regulation of I-kappaB kinase/NF-kappaB cascade, positive regulation of biological process, positive regulation of cellular process, positive regulation of signal transduction, protein kinase cascade, regulation of I-kappaB kinase/NF-kappaB cascade, regulation of biological process, regulation of cellular process, regulation of signal transduction, signal transduction, transport, | anion transporter activity, anion:cation symporter activity, carrier activity, cation transporter activity, electrochemical potential-driven transporter activity, inorganic anion transporter activity, ion transporter activity, phosphate transporter activity, porter activity, receptor activity, signal transducer activity, sodium:phosphate symporter activity, symporter activity, transporter activity, | modified residue, mutagenesis site, transmembrane region, |
| 6362 | CHEMOKINE (C-C MOTIF) LIGAND 18 (PULMONARY AND ACTIVATION-REGULATED) | extracellular region, extracellular space, | antimicrobial humoral response, antimicrobial humoral response (sensu Vertebrata), behavior, cell communication, cell-cell signaling, cellular process, chemotaxis, defense response, humoral defense mechanism (sensu Vertebrata), humoral immune response, immune response, inflammatory response, locomotory behavior, neurophysiological process, organismal physiological process, physiological process, response to abiotic stimulus, response to biotic stimulus, response to chemical stimulus, response to external stimulus, response to other organism, response to pest, pathogen or parasite, response to stimulus, response to stress, response to wounding, sensory perception, signal transduction, taxis, | G-protein-coupled receptor binding, binding, chemokine activity, chemokine receptor binding, cytokine activity, protein binding, receptor binding, signal transducer activity, | disulfide bond, signal peptide, |
| 871 | SERPIN PEPTIDASE INHIBITOR, CLADE H (HEAT SHOCK PROTEIN 47), MEMBER 1, (COLLAGEN BINDING PROTEIN 1) | ER-Golgi intermediate compartment, cell, cytoplasm, endoplasmic reticulum, intracellular, intracellular membrane-bound organelle, intracellular organelle, membrane-bound organelle, organelle, | cellular macromolecule metabolism, cellular metabolism, cellular physiological process, cellular process, cellular protein metabolism, macromolecule metabolism, metabolism, physiological process, primary metabolism, protein folding, protein metabolism, response to abiotic stimulus, response to chemical stimulus, response to protein stimulus, response to stimulus, response to stress, response to unfolded protein, | binding, collagen binding, endopeptidase inhibitor activity, enzyme inhibitor activity, enzyme regulator activity, protease inhibitor activity, protein binding, serine-type endopeptidase inhibitor activity, unfolded protein binding, | glycosylation site:N-linked (GlcNAc...), signal peptide, site:Reactive bond homolog, |
| 2817 | GLYPICAN 1 | cell, extracellular matrix, extracellular matrix (sensu Metazoa), extracellular region, extracellular space, integral to membrane, integral to plasma membrane, intrinsic to membrane, intrinsic to plasma membrane, membrane, plasma membrane, | Null | chondroitin sulfate proteoglycan, obsolete molecular function, | glycosylation site:N-linked (GlcNAc...), glycosylation site:O-linked (Xyl...) (glycosaminoglycan), lipid moiety-binding region:GPI-anchor amidated serine, propeptide:Removed in mature form, signal peptide, |
| 955 | ECTONUCLEOSIDE TRIPHOSPHATE DIPHOSPHOHYDROLASE 6 (PUTATIVE FUNCTION) | Golgi apparatus, Golgi stack, cell, cytoplasm, integral to membrane, intracellular, intracellular membrane-bound organelle, intracellular organelle, intrinsic to membrane, membrane, membrane-bound organelle, organelle, | Null | binding, calcium ion binding, catalytic activity, cation binding, hydrolase activity, hydrolase activity, acting on acid anhydrides, hydrolase activity, acting on acid anhydrides, in phosphorus-containing anhydrides, ion binding, magnesium ion binding, metal ion binding, nucleoside-diphosphatase activity, pyrophosphatase activity, | Null |
| 7127 | TUMOR NECROSIS FACTOR, ALPHA-INDUCED PROTEIN 2 | extracellular region, extracellular space, | angiogenesis, blood vessel development, blood vessel morphogenesis, cell differentiation, cellular process, development, morphogenesis, organ development, organ morphogenesis, vasculature development, | Null | Null |
| 5987 | RET FINGER PROTEIN | cell, cell fraction, integral to membrane, integral to plasma membrane, intracellular, intracellular membrane-bound organelle, intracellular organelle, intrinsic to membrane, intrinsic to plasma membrane, membrane, membrane fraction, membrane-bound organelle, nucleus, organelle, plasma membrane, protein complex, ubiquitin ligase complex, | biopolymer metabolism, biopolymer modification, cell proliferation, cellular macromolecule metabolism, cellular metabolism, cellular physiological process, cellular process, cellular protein metabolism, gametogenesis, macromolecule metabolism, male gamete generation, metabolism, nucleobase, nucleoside, nucleotide and nucleic acid metabolism, physiological process, primary metabolism, protein metabolism, protein modification, protein ubiquitination, regulation of biological process, regulation of cellular metabolism, regulation of cellular physiological process, regulation of cellular process, regulation of metabolism, regulation of nucleobase, nucleoside, nucleotide and nucleic acid metabolism, regulation of physiological process, regulation of transcription, regulation of transcription, DNA-dependent, reproduction, sexual reproduction, spermatogenesis, transcription, transcription, DNA-dependent, ubiquitin cycle, | DNA binding, binding, catalytic activity, cation binding, ion binding, kinase activity, ligase activity, ligase activity, forming carbon-nitrogen bonds, metal ion binding, nucleic acid binding, phosphotransferase activity, alcohol group as acceptor, protein binding, protein kinase activity, protein-tyrosine kinase activity, receptor activity, signal transducer activity, transferase activity, transferase activity, transferring phosphorus-containing groups, transition metal ion binding, transmembrane receptor activity, ubiquitin-protein ligase activity, zinc ion binding, | domain:B30.2-like, site:Breakpoint for translocation to form the RFP/RET oncogene, splice variant, zinc finger region:B box-type, zinc finger region:RING-type, |
| 1435 | COLONY STIMULATING FACTOR 1 (MACROPHAGE) | cell, extracellular region, extracellular space, integral to membrane, intrinsic to membrane, membrane, | cell differentiation, cell proliferation, cellular physiological process, cellular process, defense response, development, hemopoiesis, hemopoietic or lymphoid organ development, immune response, macrophage differentiation, monocyte differentiation, myeloid cell differentiation, organ development, organismal physiological process, physiological process, positive regulation of biological process, positive regulation of cell proliferation, positive regulation of cellular physiological process, positive regulation of cellular process, positive regulation of physiological process, regulation of biological process, regulation of cell proliferation, regulation of cellular physiological process, regulation of cellular process, regulation of physiological process, response to biotic stimulus, response to stimulus, | binding, cytokine activity, growth factor activity, macrophage colony stimulating factor receptor binding, protein binding, receptor binding, signal transducer activity, | disulfide bond, glycosylation site:N-linked (GlcNAc...), mutagenesis site, signal peptide, splice variant, transmembrane region, |
| 3162 | HEME OXYGENASE (DECYCLING) 1 | cell, cell fraction, cytoplasm, endoplasmic reticulum, intracellular, intracellular membrane-bound organelle, intracellular organelle, membrane fraction, membrane-bound organelle, microsome, organelle, vesicular fraction, | I-kappaB kinase/NF-kappaB cascade, catabolism, cell communication, cellular catabolism, cellular metabolism, cellular physiological process, cellular process, cofactor catabolism, cofactor metabolism, heme metabolism, heterocycle metabolism, intracellular signaling cascade, metabolism, physiological process, pigment metabolism, porphyrin catabolism, porphyrin metabolism, positive regulation of I-kappaB kinase/NF-kappaB cascade, positive regulation of biological process, positive regulation of cellular process, positive regulation of signal transduction, protein kinase cascade, regulation of I-kappaB kinase/NF-kappaB cascade, regulation of biological process, regulation of cellular process, regulation of signal transduction, secondary metabolism, signal transduction, | binding, catalytic activity, cation binding, heme oxygenase (decyclizing) activity, ion binding, iron ion binding, metal ion binding, oxidoreductase activity, signal transducer activity, transition metal ion binding, | helix, metal ion-binding site:Iron (heme axial ligand), |
| 10287 | REGULATOR OF G-PROTEIN SIGNALLING 19 | Golgi apparatus, cell, cell fraction, cytoplasm, extrinsic to membrane, extrinsic to plasma membrane, heterotrimeric G-protein complex, intracellular, intracellular membrane-bound organelle, intracellular organelle, membrane, membrane fraction, membrane-bound organelle, organelle, plasma membrane, protein complex, | G-protein coupled receptor protein signaling pathway, autophagy, cell communication, cell surface receptor linked signal transduction, cellular physiological process, cellular process, intracellular signaling cascade, negative regulation of biological process, negative regulation of cellular process, negative regulation of signal transduction, physiological process, regulation of biological process, regulation of cellular process, regulation of signal transduction, signal transduction, small GTPase mediated signal transduction, | GTPase activator activity, GTPase regulator activity, binding, enzyme activator activity, enzyme regulator activity, protein binding, signal transducer activity, | domain:RGS, mutagenesis site, |
| 9961 | MAJOR VAULT PROTEIN | cell, cytoplasm, intracellular, intracellular membrane-bound organelle, intracellular organelle, membrane-bound organelle, nucleus, organelle, protein complex, ribonucleoprotein complex, | response to abiotic stimulus, response to chemical stimulus, response to drug, response to stimulus, | Null | modified residue, repeat:Vault 1, repeat:Vault 2, repeat:Vault 3, repeat:Vault 4, repeat:Vault 5, repeat:Vault 6, repeat:Vault 7, |
| 1509 | CATHEPSIN D (LYSOSOMAL ASPARTYL PEPTIDASE) | cell, cytoplasm, extracellular region, intracellular, intracellular membrane-bound organelle, intracellular organelle, lysosome, lytic vacuole, membrane-bound organelle, organelle, vacuole, | biopolymer catabolism, biopolymer metabolism, catabolism, cellular macromolecule metabolism, cellular metabolism, cellular physiological process, cellular process, cellular protein metabolism, macromolecule catabolism, macromolecule metabolism, metabolism, physiological process, primary metabolism, protein catabolism, protein metabolism, proteolysis, | aspartic-type endopeptidase activity, catalytic activity, cathepsin D activity, endopeptidase activity, hydrolase activity, pepsin A activity, peptidase activity, | disulfide bond, glycosylation site:N-linked (GlcNAc...), propeptide:Activation peptide, signal peptide, |
| 10457 | GLYCOPROTEIN (TRANSMEMBRANE) NMB | cell, integral to membrane, intrinsic to membrane, membrane, | cell proliferation, cellular physiological process, cellular process, negative regulation of biological process, negative regulation of cell proliferation, negative regulation of cellular physiological process, negative regulation of cellular process, negative regulation of physiological process, physiological process, regulation of biological process, regulation of cell proliferation, regulation of cellular physiological process, regulation of cellular process, regulation of physiological process, | Null | domain:PKD, glycosylation site:N-linked (GlcNAc...), signal peptide, splice variant, transmembrane region, |
| 7262 | PLECKSTRIN HOMOLOGY-LIKE DOMAIN, FAMILY A, MEMBER 2 | Null | apoptosis, cell death, cellular physiological process, cellular process, death, physiological process, programmed cell death, | Null | domain:PH, |
| 23275 | PROTEIN O-FUCOSYLTRANSFERASE 2 | Null | alcohol metabolism, carbohydrate metabolism, cellular carbohydrate metabolism, cellular metabolism, cellular physiological process, cellular process, fucose metabolism, hexose metabolism, macromolecule metabolism, metabolism, monosaccharide metabolism, physiological process, primary metabolism, | catalytic activity, fucosyltransferase activity, peptide-O-fucosyltransferase activity, transferase activity, transferase activity, transferring glycosyl groups, transferase activity, transferring hexosyl groups, | glycosylation site:N-linked (GlcNAc...), signal peptide, splice variant, |
| 2720 | GALACTOSIDASE, BETA 1 | beta-galactosidase complex, cell, cytoplasm, intracellular, intracellular membrane-bound organelle, intracellular organelle, lysosome, lytic vacuole, membrane-bound organelle, organelle, protein complex, unlocalized protein complex, vacuole, | carbohydrate metabolism, macromolecule metabolism, metabolism, physiological process, primary metabolism, | beta-galactosidase activity, catalytic activity, galactosidase activity, hydrolase activity, hydrolase activity, acting on glycosyl bonds, | active site:Nucleophile, active site:Proton donor, glycosylation site:N-linked (GlcNAc...), signal peptide, |
| 55611 | OTU DOMAIN, UBIQUITIN ALDEHYDE BINDING 1 | Null | biopolymer metabolism, biopolymer modification, cellular macromolecule metabolism, cellular metabolism, cellular physiological process, cellular process, cellular protein metabolism, defense response, immune response, macromolecule metabolism, metabolism, organismal physiological process, physiological process, primary metabolism, protein metabolism, protein modification, response to biotic stimulus, response to stimulus, ubiquitin cycle, | catalytic activity, cysteine-type peptidase activity, hydrolase activity, peptidase activity, | domain:OTU, mutagenesis site, splice variant, |
| 9757 | MYELOID/LYMPHOID OR MIXED-LINEAGE LEUKEMIA 4 | cell, intracellular, intracellular membrane-bound organelle, intracellular organelle, membrane-bound organelle, nucleus, organelle, | DNA metabolism, DNA packaging, biopolymer metabolism, cell organization and biogenesis, cellular metabolism, cellular physiological process, cellular process, chromatin modification, chromatin remodeling, chromatin-mediated maintenance of transcription, chromosome organization and biogenesis, chromosome organization and biogenesis (sensu Eukaryota), development, establishment and/or maintenance of chromatin architecture, macromolecule metabolism, metabolism, nucleobase, nucleoside, nucleotide and nucleic acid metabolism, organelle organization and biogenesis, physiological process, positive regulation of biological process, positive regulation of cellular metabolism, positive regulation of cellular physiological process, positive regulation of cellular process, positive regulation of gene expression, epigenetic, positive regulation of metabolism, positive regulation of nucleobase, nucleoside, nucleotide and nucleic acid metabolism, positive regulation of physiological process, positive regulation of transcrip | DNA binding, binding, cation binding, ion binding, metal ion binding, nucleic acid binding, protein binding, transcription factor activity, transcription regulator activity, transition metal ion binding, zinc ion binding, | DNA-binding region:A.T hook 1, DNA-binding region:A.T hook 2, DNA-binding region:A.T hook 3, domain:Post-SET, domain:SET, splice variant, zinc finger region:CXXC-type, zinc finger region:PHD-type 1, zinc finger region:PHD-type 2, zinc finger region:PHD-type 3, |
| 26133 | TRANSIENT RECEPTOR POTENTIAL CATION CHANNEL, SUBFAMILY C, MEMBER 4 ASSOCIATED PROTEIN | cell, cytoplasm, cytosol, endoplasmic reticulum, endoplasmic reticulum lumen, intracellular, intracellular membrane-bound organelle, intracellular organelle, membrane-bound organelle, membrane-enclosed lumen, organelle, organelle lumen, | antigen presentation, antigen presentation, endogenous antigen, antigen presentation, endogenous peptide antigen, antigen presentation, peptide antigen, antigen processing, antigen processing, endogenous antigen via MHC class I, cell organization and biogenesis, cellular localization, cellular physiological process, cellular process, cytosol to ER transport, defense response, establishment of cellular localization, establishment of localization, establishment of protein localization, immune response, intracellular protein transport, intracellular transport, localization, macromolecule metabolism, metabolism, organismal physiological process, peptide transport, physiological process, primary metabolism, protein complex assembly, protein localization, protein metabolism, protein transport, response to biotic stimulus, response to stimulus, transport, | ATP binding, ATPase activity, ATPase activity, coupled, ATPase activity, coupled to movement of substances, ATPase activity, coupled to transmembrane movement of substances, MHC class I protein binding, MHC protein binding, TAP binding, TAP1 binding, TAP2 binding, adenyl nucleotide binding, antigen binding, binding, catalytic activity, hydrolase activity, hydrolase activity, acting on acid anhydrides, hydrolase activity, acting on acid anhydrides, catalyzing transmembrane movement of substances, hydrolase activity, acting on acid anhydrides, in phosphorus-containing anhydrides, identical protein binding, nucleoside-triphosphatase activity, nucleotide binding, peptide antigen binding, peptide antigen-transporting ATPase activity, peptide binding, peptide transporter activity, peptide-transporting ATPase activity, phosphate binding, protein binding, protein dimerization activity, protein heterodimerization activity, protein homodimerization activity, purine nucleotide binding, pyrophosphatase activity, receptor | Null |
| 311 | ANNEXIN A11 | cell, cytoplasm, endomembrane system, envelope, intracellular, intracellular membrane-bound organelle, intracellular organelle, membrane, membrane-bound organelle, membrane-enclosed lumen, nuclear envelope, nuclear lumen, nucleoplasm, nucleus, organelle, organelle envelope, organelle lumen, | defense response, immune response, organismal physiological process, physiological process, response to biotic stimulus, response to stimulus, | binding, calcium ion binding, calcium-dependent phospholipid binding, cation binding, ion binding, lipid binding, metal ion binding, phospholipid binding, protein binding, | repeat:Annexin 1, repeat:Annexin 2, repeat:Annexin 3, repeat:Annexin 4, |
| 4061 | LYMPHOCYTE ANTIGEN 6 COMPLEX, LOCUS E | cell, integral to membrane, integral to plasma membrane, intrinsic to membrane, intrinsic to plasma membrane, membrane, plasma membrane, | cell communication, cell surface receptor linked signal transduction, cellular process, defense response, response to biotic stimulus, response to stimulus, signal transduction, | Null | disulfide bond, domain:UPAR/Ly6, glycosylation site:N-linked (GlcNAc...), lipid moiety-binding region:GPI-anchor amidated serine, propeptide:Removed in mature form, signal peptide, |
| 6793 | SERINE/THREONINE KINASE 10 | Null | biopolymer metabolism, biopolymer modification, cellular macromolecule metabolism, cellular metabolism, cellular physiological process, cellular process, cellular protein metabolism, macromolecule metabolism, metabolism, phosphate metabolism, phosphorus metabolism, phosphorylation, physiological process, primary metabolism, protein amino acid phosphorylation, protein metabolism, protein modification, | ATP binding, adenyl nucleotide binding, binding, cAMP-dependent protein kinase activity, catalytic activity, cyclic nucleotide-dependent protein kinase activity, kinase activity, nucleotide binding, phosphotransferase activity, alcohol group as acceptor, protein kinase CK2 activity, protein kinase activity, protein serine/threonine kinase activity, purine nucleotide binding, transferase activity, transferase activity, transferring phosphorus-containing groups, | active site:Proton acceptor, binding site:ATP, domain:Protein kinase, nucleotide phosphate-binding region:ATP, |
| 713 | COMPLEMENT COMPONENT 1, Q SUBCOMPONENT, B CHAIN | cell, cytoplasm, extracellular region, extracellular space, intracellular, protein complex, | anion transport, cellular physiological process, cellular process, complement activation, complement activation, classical pathway, defense response, establishment of localization, humoral defense mechanism (sensu Vertebrata), humoral immune response, immune response, innate immune response, inorganic anion transport, ion transport, localization, organismal physiological process, phosphate transport, physiological process, response to biotic stimulus, response to other organism, response to pest, pathogen or parasite, response to stimulus, response to stress, transport, | Null | disulfide bond, domain:C1q, domain:Collagen-like, glycosylation site:O-linked (Gal...), signal peptide, |
| 8714 | ATP-BINDING CASSETTE, SUB-FAMILY C (CFTR/MRP), MEMBER 3 | cell, cell fraction, integral to membrane, integral to plasma membrane, intrinsic to membrane, intrinsic to plasma membrane, membrane, membrane fraction, plasma membrane, | cellular physiological process, cellular process, establishment of localization, localization, physiological process, transport, | ATP binding, ATPase activity, ATPase activity, coupled, ATPase activity, coupled to movement of substances, ATPase activity, coupled to transmembrane movement of substances, adenyl nucleotide binding, anion transporter activity, binding, catalytic activity, hydrolase activity, hydrolase activity, acting on acid anhydrides, hydrolase activity, acting on acid anhydrides, catalyzing transmembrane movement of substances, hydrolase activity, acting on acid anhydrides, in phosphorus-containing anhydrides, ion transporter activity, nucleoside-triphosphatase activity, nucleotide binding, organic anion transporter activity, purine nucleotide binding, pyrophosphatase activity, transporter activity, | domain:ABC transmembrane type-1 1, domain:ABC transmembrane type-1 2, domain:ABC transporter 1, domain:ABC transporter 2, glycosylation site:N-linked (GlcNAc...), nucleotide phosphate-binding region:ATP 1, nucleotide phosphate-binding region:ATP 2, splice variant, transmembrane region, |
| 2944 | GLUTATHIONE S-TRANSFERASE M1 | cell, cytoplasm, intracellular, | metabolism, physiological process, | catalytic activity, glutathione transferase activity, transferase activity, transferase activity, transferring alkyl or aryl (other than methyl) groups, | helix, mutagenesis site, |
| 3587 | INTERLEUKIN 10 RECEPTOR, ALPHA | cell, integral to membrane, intrinsic to membrane, membrane, plasma membrane, | Null | binding, cytokine binding, growth factor binding, interleukin binding, interleukin receptor activity, interleukin-10 binding, interleukin-10 receptor activity, protein binding, receptor activity, signal transducer activity, transmembrane receptor activity, | disulfide bond, glycosylation site:N-linked (GlcNAc...), signal peptide, transmembrane region, |
| 752 | FORMIN-LIKE 1 | cellular component unknown, | actin cytoskeleton organization and biogenesis, actin filament-based process, biological process unknown, cell organization and biogenesis, cellular physiological process, cellular process, cytoskeleton organization and biogenesis, organelle organization and biogenesis, physiological process, | GTPase binding, Rho GTPase binding, actin binding, binding, cytoskeletal protein binding, enzyme binding, molecular function unknown, protein binding, small GTPase binding, | domain:FH2, domain:FH3, splice variant, |
| 162 | ADAPTOR-RELATED PROTEIN COMPLEX 1, BETA 1 SUBUNIT | Golgi apparatus, Golgi membrane, Golgi stack, Golgi-associated vesicle, Golgi-associated vesicle membrane, cell, clathrin coat, clathrin coat of trans-Golgi network vesicle, clathrin coated vesicle membrane, clathrin vesicle coat, clathrin-coated vesicle, coated membrane, coated pit, coated vesicle, coated vesicle membrane, cytoplasm, cytoplasmic membrane-bound vesicle, cytoplasmic vesicle, cytoplasmic vesicle membrane, endomembrane system, intracellular, intracellular membrane-bound organelle, intracellular organelle, membrane, membrane coat, membrane-bound organelle, membrane-bound vesicle, organelle, organelle membrane, plasma membrane, trans-Golgi network transport vesicle, trans-Golgi network transport vesicle membrane, transport vesicle, transport vesicle membrane, vesicle, vesicle coat, vesicle membrane, | cell organization and biogenesis, cellular localization, cellular physiological process, cellular process, endocytosis, establishment of cellular localization, establishment of localization, establishment of protein localization, intracellular protein transport, intracellular transport, localization, macromolecule metabolism, metabolism, physiological process, primary metabolism, protein complex assembly, protein localization, protein metabolism, protein transport, transport, vesicle-mediated transport, | binding, protein binding, transporter activity, | splice variant, |
| 717 | COMPLEMENT COMPONENT 2 | extracellular region, extracellular space, protein complex, | cellular macromolecule metabolism, cellular metabolism, cellular physiological process, cellular process, cellular protein metabolism, complement activation, complement activation, classical pathway, defense response, humoral defense mechanism (sensu Vertebrata), humoral immune response, immune response, innate immune response, macromolecule metabolism, metabolism, organismal physiological process, physiological process, primary metabolism, protein metabolism, proteolysis, response to biotic stimulus, response to other organism, response to pest, pathogen or parasite, response to stimulus, response to stress, | catalytic activity, classical-complement-pathway C3/C5 convertase activity, endopeptidase activity, hydrolase activity, peptidase activity, serine-type endopeptidase activity, serine-type peptidase activity, | active site:Charge relay system, disulfide bond, domain:Peptidase S1, domain:Sushi 1, domain:Sushi 2, domain:Sushi 3, domain:VWFA, glycosylation site:N-linked (GlcNAc...), signal peptide, |
| 6280 | S100 CALCIUM BINDING PROTEIN A9 (CALGRANULIN B) | extracellular region, extracellular space, | cell communication, cell-cell signaling, cellular process, defense response, immune response, inflammatory response, organismal physiological process, physiological process, response to biotic stimulus, response to external stimulus, response to other organism, response to pest, pathogen or parasite, response to stimulus, response to stress, response to wounding, | binding, calcium ion binding, cation binding, ion binding, metal ion binding, signal transducer activity, | calcium-binding region:1; low affinity, calcium-binding region:2; high affinity, domain:EF-hand 1, domain:EF-hand 2, |
| 5499 | PROTEIN PHOSPHATASE 1, CATALYTIC SUBUNIT, ALPHA ISOFORM | Null | biopolymer metabolism, carbohydrate metabolism, cell cycle, cell division, cellular carbohydrate metabolism, cellular macromolecule metabolism, cellular metabolism, cellular physiological process, cellular polysaccharide metabolism, cellular process, generation of precursor metabolites and energy, glucan metabolism, glycogen metabolism, macromolecule metabolism, metabolism, physiological process, polysaccharide metabolism, primary metabolism, | CTD phosphatase activity, binding, calcium-dependent protein serine/threonine phosphatase activity, catalytic activity, cation binding, hydrolase activity, hydrolase activity, acting on ester bonds, ion binding, iron ion binding, magnesium-dependent protein serine/threonine phosphatase activity, manganese ion binding, metal ion binding, myosin phosphatase activity, phosphoprotein phosphatase activity, phosphoric ester hydrolase activity, phosphoric monoester hydrolase activity, protein phosphatase type 1 activity, protein phosphatase type 2A activity, protein phosphatase type 2B activity, protein phosphatase type 2C activity, protein serine/threonine phosphatase activity, transition metal ion binding, | active site:Proton donor, metal ion-binding site:Iron, metal ion-binding site:Manganese, |
| 1116 | CHITINASE 3-LIKE 1 (CARTILAGE GLYCOPROTEIN-39) | extracellular matrix, extracellular matrix (sensu Metazoa), extracellular region, extracellular space, | N-acetylglucosamine catabolism, N-acetylglucosamine metabolism, amine metabolism, amino sugar catabolism, amino sugar metabolism, biopolymer catabolism, biopolymer metabolism, carbohydrate catabolism, carbohydrate metabolism, catabolism, cellular carbohydrate catabolism, cellular carbohydrate metabolism, cellular catabolism, cellular macromolecule catabolism, cellular macromolecule metabolism, cellular metabolism, cellular physiological process, cellular polysaccharide catabolism, cellular polysaccharide metabolism, cellular process, chitin catabolism, chitin metabolism, glucosamine catabolism, glucosamine metabolism, macromolecule catabolism, macromolecule metabolism, metabolism, nitrogen compound metabolism, physiological process, polysaccharide catabolism, polysaccharide metabolism, primary metabolism, | binding, carbohydrate binding, catalytic activity, chitinase activity, extracellular matrix structural constituent, hydrolase activity, hydrolase activity, acting on glycosyl bonds, structural molecule activity, sugar binding, | disulfide bond, glycosylation site:N-linked (GlcNAc...), signal peptide, |
| 2217 | FC FRAGMENT OF IGG, RECEPTOR, TRANSPORTER, ALPHA | MHC class I protein complex, MHC protein complex, cell, immunological synapse, integral to membrane, intrinsic to membrane, membrane, plasma membrane, protein complex, | antigen presentation, antigen presentation, endogenous antigen, antigen processing, antigen processing, endogenous antigen via MHC class I, defense response, immune response, interaction between organisms, organismal physiological process, physiological interaction between organisms, physiological process, pregnancy, reproduction, reproductive organismal physiological process, reproductive physiological process, response to biotic stimulus, response to stimulus, | IgG binding, MHC class I receptor activity, binding, immunoglobulin binding, protein binding, receptor activity, signal transducer activity, transmembrane receptor activity, | disulfide bond, glycosylation site:N-linked (GlcNAc...), signal peptide, transmembrane region, |
| 6892 | TAP BINDING PROTEIN (TAPASIN) | Golgi apparatus, Golgi membrane, MHC class I peptide loading complex, cell, cell fraction, cytoplasm, endomembrane system, endoplasmic reticulum, endoplasmic reticulum membrane, integral to membrane, intracellular, intracellular membrane-bound organelle, intracellular organelle, intrinsic to membrane, membrane, membrane fraction, membrane-bound organelle, microsome, nuclear envelope-endoplasmic reticulum network, organelle, organelle membrane, protein complex, vesicular fraction, | Golgi vesicle transport, antigen processing, antigen processing, endogenous antigen via MHC class I, cell organization and biogenesis, cellular localization, cellular metabolism, cellular physiological process, cellular process, defense response, establishment of cellular localization, establishment of localization, immune response, intracellular transport, localization, macromolecule metabolism, metabolism, organismal physiological process, peptide antigen stabilization, peptide metabolism, peptide stabilization, physiological process, primary metabolism, protein complex assembly, protein metabolism, response to biotic stimulus, response to stimulus, retrograde vesicle-mediated transport, Golgi to ER, secretion, secretory pathway, transport, vesicle-mediated transport, | ATPase activity, ATPase activity, coupled, ATPase activity, coupled to movement of substances, ATPase activity, coupled to transmembrane movement of substances, MHC class I protein binding, MHC protein binding, TAP binding, TAP1 binding, TAP2 binding, antigen binding, binding, catalytic activity, hydrolase activity, hydrolase activity, acting on acid anhydrides, hydrolase activity, acting on acid anhydrides, catalyzing transmembrane movement of substances, hydrolase activity, acting on acid anhydrides, in phosphorus-containing anhydrides, nucleoside-triphosphatase activity, peptide antigen binding, peptide antigen-transporting ATPase activity, peptide binding, peptide transporter activity, peptide-transporting ATPase activity, protein binding, pyrophosphatase activity, receptor binding, signal transducer activity, transporter activity, unfolded protein binding, | disulfide bond, domain:Ig-like C1-type, glycosylation site:N-linked (GlcNAc...), signal peptide, site:May be involved in interaction with TAP, splice variant, transmembrane region, |
| 22838 | RING FINGER PROTEIN 44 | cell, intracellular, intracellular membrane-bound organelle, intracellular organelle, membrane-bound organelle, nucleus, organelle, protein complex, ubiquitin ligase complex, | biopolymer metabolism, biopolymer modification, cellular macromolecule metabolism, cellular metabolism, cellular physiological process, cellular process, cellular protein metabolism, macromolecule metabolism, metabolism, nucleobase, nucleoside, nucleotide and nucleic acid metabolism, physiological process, primary metabolism, protein metabolism, protein modification, protein ubiquitination, regulation of biological process, regulation of cellular metabolism, regulation of cellular physiological process, regulation of cellular process, regulation of metabolism, regulation of nucleobase, nucleoside, nucleotide and nucleic acid metabolism, regulation of physiological process, regulation of transcription, regulation of transcription, DNA-dependent, transcription, transcription, DNA-dependent, ubiquitin cycle, | DNA binding, binding, catalytic activity, cation binding, ion binding, ligase activity, ligase activity, forming carbon-nitrogen bonds, metal ion binding, nucleic acid binding, protein binding, transcription factor activity, transcription regulator activity, transition metal ion binding, ubiquitin-protein ligase activity, zinc ion binding, | Null |
| 9853 | RUN AND SH3 DOMAIN CONTAINING 2 | Null | Null | Null | domain:RUN, domain:SH3, |
| 1263 | POLO-LIKE KINASE 3 (DROSOPHILA) | Null | biopolymer metabolism, biopolymer modification, cell cycle, cellular macromolecule metabolism, cellular metabolism, cellular physiological process, cellular process, cellular protein metabolism, macromolecule metabolism, metabolism, phosphate metabolism, phosphorus metabolism, phosphorylation, physiological process, primary metabolism, protein amino acid phosphorylation, protein metabolism, protein modification, regulation of biological process, regulation of cell cycle, regulation of cellular physiological process, regulation of cellular process, regulation of physiological process, regulation of progression through cell cycle, | ATP binding, adenyl nucleotide binding, binding, catalytic activity, kinase activity, nucleotide binding, phosphotransferase activity, alcohol group as acceptor, protein binding, protein kinase activity, protein serine/threonine kinase activity, purine nucleotide binding, transferase activity, transferase activity, transferring phosphorus-containing groups, | active site:Proton acceptor, binding site:ATP, domain:POLO box 1, domain:POLO box 2, domain:Protein kinase, nucleotide phosphate-binding region:ATP, |
| 4669 | N-ACETYLGLUCOSAMINIDASE, ALPHA- (SANFILIPPO DISEASE IIIB) | cell, cytoplasm, intracellular, intracellular membrane-bound organelle, intracellular organelle, lysosome, lytic vacuole, membrane-bound organelle, organelle, vacuole, | amine metabolism, aminoglycan metabolism, carbohydrate metabolism, cellular carbohydrate metabolism, cellular metabolism, cellular physiological process, cellular process, development, glycosaminoglycan metabolism, macromolecule metabolism, metabolism, nervous system development, nitrogen compound metabolism, physiological process, primary metabolism, system development, | alpha-N-acetylglucosaminidase activity, catalytic activity, hexosaminidase activity, hydrolase activity, hydrolase activity, acting on glycosyl bonds, | glycosylation site:N-linked (GlcNAc...), signal peptide, |
| 4320 | MATRIX METALLOPEPTIDASE 11 (STROMELYSIN 3) | extracellular matrix, extracellular matrix (sensu Metazoa), extracellular region, | biopolymer catabolism, biopolymer metabolism, catabolism, cellular catabolism, cellular macromolecule catabolism, cellular macromolecule metabolism, cellular metabolism, cellular physiological process, cellular process, cellular protein catabolism, cellular protein metabolism, collagen catabolism, development, macromolecule catabolism, macromolecule metabolism, metabolism, morphogenesis, physiological process, primary metabolism, protein catabolism, protein metabolism, proteolysis, proteolysis during cellular protein catabolism, | binding, calcium ion binding, catalytic activity, cation binding, endopeptidase activity, hydrolase activity, ion binding, metal ion binding, metalloendopeptidase activity, peptidase activity, transition metal ion binding, zinc ion binding, | disulfide bond, domain:Hemopexin-like, metal ion-binding site:Calcium, metal ion-binding site:Calcium (via carbonyl oxygen), metal ion-binding site:Zinc 1, metal ion-binding site:Zinc 2 (catalytic), propeptide:Activation peptide, signal peptide, site:Cysteine switch, |
| 4245 | MANNOSYL (ALPHA-1,3-)-GLYCOPROTEIN BETA-1,2-N-ACETYLGLUCOSAMINYLTRANSFERASE | Golgi apparatus, Golgi membrane, Golgi stack, cell, cytoplasm, endomembrane system, integral to membrane, intracellular, intracellular membrane-bound organelle, intracellular organelle, intrinsic to membrane, membrane, membrane-bound organelle, organelle, organelle membrane, | amine metabolism, aminoglycan biosynthesis, aminoglycan metabolism, biopolymer glycosylation, biopolymer metabolism, biopolymer modification, biosynthesis, carbohydrate biosynthesis, carbohydrate metabolism, cellular biosynthesis, cellular carbohydrate metabolism, cellular macromolecule metabolism, cellular metabolism, cellular physiological process, cellular process, cellular protein metabolism, glycoprotein biosynthesis, glycoprotein metabolism, macromolecule biosynthesis, macromolecule metabolism, metabolism, nitrogen compound metabolism, physiological process, primary metabolism, protein amino acid N-linked glycosylation, protein amino acid glycosylation, protein biosynthesis, protein metabolism, protein modification, | UDP-glycosyltransferase activity, acetylglucosaminyltransferase activity, alpha-1,3-mannosylglycoprotein 2-beta-N-acetylglucosaminyltransferase activity, catalytic activity, transferase activity, transferase activity, transferring glycosyl groups, transferase activity, transferring hexosyl groups, | sequence conflict, transmembrane region, |
| 2896 | GRANULIN | extracellular region, extracellular space, | cell communication, cell proliferation, cell-cell signaling, cellular physiological process, cellular process, physiological process, positive regulation of biological process, positive regulation of cell proliferation, positive regulation of cellular physiological process, positive regulation of cellular process, positive regulation of physiological process, regulation of biological process, regulation of cell proliferation, regulation of cellular physiological process, regulation of cellular process, regulation of physiological process, signal transduction, | binding, cytokine activity, growth factor activity, protein binding, receptor binding, signal transducer activity, | disulfide bond, glycosylation site:N-linked (GlcNAc...), peptide:Granulin-1, peptide:Granulin-2, peptide:Granulin-3, peptide:Granulin-4, peptide:Granulin-5, peptide:Granulin-6, peptide:Granulin-7, peptide:Paragranulin, signal peptide, splice variant, |
| 3055 | HEMOPOIETIC CELL KINASE | Null | biopolymer metabolism, biopolymer modification, cell communication, cellular macromolecule metabolism, cellular metabolism, cellular physiological process, cellular process, cellular protein metabolism, development, intracellular signaling cascade, macromolecule metabolism, mesoderm development, metabolism, phosphate metabolism, phosphorus metabolism, phosphorylation, physiological process, primary metabolism, protein amino acid phosphorylation, protein metabolism, protein modification, signal transduction, tissue development, | ATP binding, adenyl nucleotide binding, binding, catalytic activity, kinase activity, nucleotide binding, phosphotransferase activity, alcohol group as acceptor, protein binding, protein kinase activity, protein-tyrosine kinase activity, purine nucleotide binding, transferase activity, transferase activity, transferring phosphorus-containing groups, | active site:Proton acceptor, binding site:ATP, domain:Protein kinase, domain:SH2, domain:SH3, lipid moiety-binding region:N-myristoyl glycine, lipid moiety-binding region:N-myristoyl glycine (in isoform P59-HCK), lipid moiety-binding region:S-palmitoyl cysteine (in isoform P59-HCK), nucleotide phosphate-binding region:ATP, |
| 7305 | TYRO PROTEIN TYROSINE KINASE BINDING PROTEIN | cell, integral to membrane, integral to plasma membrane, intrinsic to membrane, intrinsic to plasma membrane, membrane, plasma membrane, | cell communication, cellular defense response, cellular process, defense response, immune response, intracellular signaling cascade, organismal physiological process, physiological process, response to biotic stimulus, response to external stimulus, response to other organism, response to pest, pathogen or parasite, response to stimulus, response to stress, response to wounding, signal transduction, | signal transducer activity, | sequence variant, signal peptide, splice variant, transmembrane region, |
| 101 | ADAM METALLOPEPTIDASE DOMAIN 8 | cell, integral to membrane, integral to plasma membrane, intrinsic to membrane, intrinsic to plasma membrane, membrane, plasma membrane, | cell communication, cell surface receptor linked signal transduction, cellular macromolecule metabolism, cellular metabolism, cellular physiological process, cellular process, cellular protein metabolism, integrin-mediated signaling pathway, macromolecule metabolism, metabolism, physiological process, primary metabolism, protein metabolism, proteolysis, signal transduction, | binding, catalytic activity, cation binding, endopeptidase activity, hydrolase activity, ion binding, metal ion binding, metalloendopeptidase activity, peptidase activity, transition metal ion binding, zinc ion binding, | disulfide bond, domain:Disintegrin, domain:Peptidase M12B, glycosylation site:N-linked (GlcNAc...), metal ion-binding site:Zinc (catalytic), signal peptide, transmembrane region, |
| 22846 | VASOHIBIN 1 | Null | cell cycle, cell cycle arrest, cellular physiological process, cellular process, negative regulation of biological process, negative regulation of cellular physiological process, negative regulation of cellular process, negative regulation of physiological process, negative regulation of progression through cell cycle, physiological process, regulation of biological process, regulation of cell cycle, regulation of cellular physiological process, regulation of cellular process, regulation of physiological process, regulation of progression through cell cycle, | Null | splice variant, |
| 1209 | CLEFT LIP AND PALATE ASSOCIATED TRANSMEMBRANE PROTEIN 1 | cell, integral to membrane, integral to plasma membrane, intrinsic to membrane, intrinsic to plasma membrane, membrane, plasma membrane, | development, | Null | Null |
| 5514 | PROTEIN PHOSPHATASE 1, REGULATORY SUBUNIT 10 | cell, intracellular, intracellular membrane-bound organelle, intracellular organelle, membrane-bound organelle, nucleus, organelle, | cellular metabolism, cellular physiological process, cellular process, defense response, metabolism, nucleobase, nucleoside, nucleotide and nucleic acid metabolism, physiological process, primary metabolism, response to biotic stimulus, response to stimulus, transcription, | DNA binding, RNA binding, binding, cation binding, enzyme inhibitor activity, enzyme regulator activity, ion binding, metal ion binding, nucleic acid binding, phosphatase inhibitor activity, phosphatase regulator activity, protein phosphatase inhibitor activity, protein phosphatase regulator activity, transition metal ion binding, zinc ion binding, | domain:TFS2-N, zinc finger region:C3H1-type, |
| 11273 | ATAXIN 2-LIKE | cell, cellular component unknown, membrane, | biological process unknown, | molecular function unknown, | modified residue, splice variant, |
| 2580 | CYCLIN G ASSOCIATED KINASE | Golgi apparatus, Golgi stack, cell, cytoplasm, intracellular, intracellular membrane-bound organelle, intracellular organelle, membrane-bound organelle, nucleus, organelle, | biopolymer metabolism, biopolymer modification, cell cycle, cellular macromolecule metabolism, cellular metabolism, cellular physiological process, cellular process, cellular protein metabolism, macromolecule metabolism, metabolism, phosphate metabolism, phosphorus metabolism, phosphorylation, physiological process, primary metabolism, protein amino acid phosphorylation, protein folding, protein metabolism, protein modification, regulation of biological process, regulation of cell cycle, regulation of cellular physiological process, regulation of cellular process, regulation of physiological process, regulation of progression through cell cycle, | ATP binding, adenyl nucleotide binding, binding, catalytic activity, heat shock protein binding, kinase activity, nucleotide binding, phosphotransferase activity, alcohol group as acceptor, protein binding, protein kinase activity, protein serine/threonine kinase activity, purine nucleotide binding, transferase activity, transferase activity, transferring phosphorus-containing groups, unfolded protein binding, | Null |
| 6279 | S100 CALCIUM BINDING PROTEIN A8 (CALGRANULIN A) | extracellular region, extracellular space, | defense response, immune response, inflammatory response, organismal physiological process, physiological process, response to biotic stimulus, response to external stimulus, response to other organism, response to pest, pathogen or parasite, response to stimulus, response to stress, response to wounding, | binding, calcium ion binding, cation binding, cytokine activity, ion binding, metal ion binding, protein binding, receptor binding, signal transducer activity, | calcium-binding region:1; low affinity, calcium-binding region:2; high affinity, domain:EF-hand 1, domain:EF-hand 2, |
| 348 | APOLIPOPROTEIN E | cell, chylomicron, cytoplasm, extracellular region, extracellular space, intracellular, | apoptosis, axon extension, axon guidance, axonogenesis, behavior, cell communication, cell death, cell development, cell differentiation, cell migration, cell motility, cell organization and biogenesis, cell-cell signaling, cellular localization, cellular macromolecule metabolism, cellular metabolism, cellular morphogenesis, cellular morphogenesis during differentiation, cellular physiological process, cellular process, cellular protein metabolism, cholesterol homeostasis, circulation, cytoskeleton organization and biogenesis, death, development, establishment of cellular localization, establishment of localization, homeostasis, induction of apoptosis, induction of programmed cell death, intracellular transport, learning and/or memory, lipid binding, lipid transport, lipoprotein metabolism, localization, localization of cell, locomotion, macromolecule metabolism, metabolism, morphogenesis, nerve-nerve synaptic transmission, nervous system development, neurite morphogenesis, neurogenesis, neuron development, n | antioxidant activity, apolipoprotein E receptor binding, beta-amyloid binding, binding, carbohydrate binding, cytoskeletal protein binding, glycosaminoglycan binding, heparin binding, high-density lipoprotein, lipid binding, lipid transporter activity, low-density lipoprotein receptor binding, obsolete molecular function, pattern binding, phospholipid binding, polysaccharide binding, protein binding, receptor binding, signal transducer activity, tau protein binding, transporter activity, very-low-density lipoprotein, | glycosylation site:O-linked (GalNAc...), repeat:1, repeat:2, repeat:3, repeat:4, repeat:5, repeat:6, repeat:7, repeat:8, signal peptide, |
| 9473 | CHROMOSOME 1 OPEN READING FRAME 38 | Null | Null | Null | sequence conflict, splice variant, |
| 3861 | KERATIN 14 (EPIDERMOLYSIS BULLOSA SIMPLEX, DOWLING-MEARA, KOEBNER) | cell, cellular component unknown, cytoskeleton, intermediate filament, intermediate filament cytoskeleton, intracellular, intracellular non-membrane-bound organelle, intracellular organelle, non-membrane-bound organelle, organelle, | biological process unknown, development, ectoderm development, epidermis development, tissue development, | structural constituent of cytoskeleton, structural constituent of epidermis, structural molecule activity, | sequence conflict, site:Stutter, |
| 6721 | STEROL REGULATORY ELEMENT BINDING TRANSCRIPTION FACTOR 2 | Golgi apparatus, Golgi stack, cell, cytoplasm, endoplasmic reticulum, integral to membrane, intracellular, intracellular membrane-bound organelle, intracellular organelle, intrinsic to membrane, membrane, membrane-bound organelle, nucleus, organelle, | alcohol metabolism, cellular lipid metabolism, cellular metabolism, cellular physiological process, cellular process, cholesterol metabolism, lipid metabolism, metabolism, nucleobase, nucleoside, nucleotide and nucleic acid metabolism, physiological process, primary metabolism, regulation of biological process, regulation of cellular metabolism, regulation of cellular physiological process, regulation of cellular process, regulation of metabolism, regulation of nucleobase, nucleoside, nucleotide and nucleic acid metabolism, regulation of physiological process, regulation of transcription, regulation of transcription from RNA polymerase II promoter, regulation of transcription, DNA-dependent, steroid metabolism, sterol metabolism, transcription, transcription from RNA polymerase II promoter, transcription, DNA-dependent, | DNA binding, RNA polymerase II transcription factor activity, binding, nucleic acid binding, protein binding, transcription factor activity, transcription regulator activity, | DNA-binding region:Basic motif, domain:Helix-loop-helix motif, domain:Leucine-zipper, mutagenesis site, site:Cleavage (by S1P), site:Cleavage (by S2P), site:Cleavage (by caspase-3 and caspase-7), transmembrane region, |
| 10379 | INTERFERON-STIMULATED TRANSCRIPTION FACTOR 3, GAMMA 48KDA | cell, cytoplasm, intracellular, intracellular membrane-bound organelle, intracellular organelle, membrane-bound organelle, nucleus, organelle, | cell communication, cell surface receptor linked signal transduction, cellular metabolism, cellular physiological process, cellular process, defense response, immune response, metabolism, nucleobase, nucleoside, nucleotide and nucleic acid metabolism, organismal physiological process, physiological process, primary metabolism, regulation of biological process, regulation of cellular metabolism, regulation of cellular physiological process, regulation of cellular process, regulation of metabolism, regulation of nucleobase, nucleoside, nucleotide and nucleic acid metabolism, regulation of physiological process, regulation of transcription, regulation of transcription, DNA-dependent, response to biotic stimulus, response to other organism, response to pest, pathogen or parasite, response to stimulus, response to stress, response to virus, signal transduction, transcription, transcription from RNA polymerase II promoter, transcription, DNA-dependent, | DNA binding, binding, cation binding, ion binding, metal ion binding, nucleic acid binding, protein binding, transcription factor activity, transcription regulator activity, transition metal ion binding, zinc ion binding, | DNA-binding region:Tryptophan pentad repeat, domain:UBA, splice variant, zinc finger region:IBR-type 1, zinc finger region:IBR-type 2, zinc finger region:RING-type; degenerate, zinc finger region:RanBP2-type 1, zinc finger region:RanBP2-type 2, zinc finger region:RanBP2-type 3, |
| 55072 | INTERFERON-STIMULATED TRANSCRIPTION FACTOR 3, GAMMA 48KDA | cell, cytoplasm, intracellular, intracellular membrane-bound organelle, intracellular organelle, membrane-bound organelle, nucleus, organelle, | cell communication, cell surface receptor linked signal transduction, cellular metabolism, cellular physiological process, cellular process, defense response, immune response, metabolism, nucleobase, nucleoside, nucleotide and nucleic acid metabolism, organismal physiological process, physiological process, primary metabolism, regulation of biological process, regulation of cellular metabolism, regulation of cellular physiological process, regulation of cellular process, regulation of metabolism, regulation of nucleobase, nucleoside, nucleotide and nucleic acid metabolism, regulation of physiological process, regulation of transcription, regulation of transcription, DNA-dependent, response to biotic stimulus, response to other organism, response to pest, pathogen or parasite, response to stimulus, response to stress, response to virus, signal transduction, transcription, transcription from RNA polymerase II promoter, transcription, DNA-dependent, | DNA binding, binding, cation binding, ion binding, metal ion binding, nucleic acid binding, protein binding, transcription factor activity, transcription regulator activity, transition metal ion binding, zinc ion binding, | DNA-binding region:Tryptophan pentad repeat, domain:UBA, splice variant, zinc finger region:IBR-type 1, zinc finger region:IBR-type 2, zinc finger region:RING-type; degenerate, zinc finger region:RanBP2-type 1, zinc finger region:RanBP2-type 2, zinc finger region:RanBP2-type 3, |
| 23646 | PHOSPHOLIPASE D FAMILY, MEMBER 3 | cell, membrane, | metabolism, physiological process, | carboxylic ester hydrolase activity, catalytic activity, hydrolase activity, hydrolase activity, acting on ester bonds, lipase activity, phospholipase D activity, phospholipase activity, phosphoric diester hydrolase activity, phosphoric ester hydrolase activity, phosphoric monoester hydrolase activity, | Null |
| 10184 | LIPOMA HMGIC FUSION PARTNER-LIKE 2 | Null | Null | Null | Null |
| **Down-regulated** | | | | | |
| 1645 | ALDO-KETO REDUCTASE FAMILY 1, MEMBER C1 (DIHYDRODIOL DEHYDROGENASE 1; 20-ALPHA (3-ALPHA)-HYDROXYSTEROID DEHYDROGENASE) | cell, cytoplasm, intracellular, | cellular metabolism, cellular physiological process, cellular process, metabolism, physiological process, response to abiotic stimulus, response to chemical stimulus, response to stimulus, response to xenobiotic stimulus, xenobiotic metabolism, | 20-alpha-hydroxysteroid dehydrogenase activity, aldo-keto reductase activity, bile acid transporter activity, binding, carboxylic acid transporter activity, catalytic activity, electron transporter activity, monocarboxylic acid transporter activity, organic acid transporter activity, oxidoreductase activity, oxidoreductase activity, acting on CH-OH group of donors, oxidoreductase activity, acting on the CH-CH group of donors, oxidoreductase activity, acting on the CH-CH group of donors, NAD or NADP as acceptor, oxidoreductase activity, acting on the CH-OH group of donors, NAD or NADP as acceptor, trans-1,2-dihydrobenzene-1,2-diol dehydrogenase activity, transporter activity, | active site:Proton donor, binding site:Progesterone, binding site:Substrate, mutagenesis site, nucleotide phosphate-binding region:NADP, site:Important for substrate specificity, site:Lowers pKa of active site Tyr, site:May be involved in the mediating step between the transformation of progesterone and the release of the cofactor, |
| 54677 | CARNITINE O-OCTANOYLTRANSFERASE | cell, cytoplasm, intracellular, intracellular membrane-bound organelle, intracellular organelle, membrane-bound organelle, microbody, organelle, peroxisome, | carboxylic acid metabolism, cellular lipid metabolism, cellular metabolism, cellular physiological process, cellular process, establishment of localization, fatty acid metabolism, generation of precursor metabolites and energy, lipid metabolism, localization, metabolism, organic acid metabolism, physiological process, primary metabolism, transport, | O-acyltransferase activity, O-octanoyltransferase activity, acyltransferase activity, carnitine O-acyltransferase activity, carnitine O-octanoyltransferase activity, catalytic activity, octanoyltransferase activity, transferase activity, transferase activity, transferring acyl groups, transferase activity, transferring groups other than amino-acyl groups, | active site:Proton acceptor, binding site:Carnitine, binding site:Coenzyme A, |
| 5728 | PHOSPHATASE AND TENSIN HOMOLOG (MUTATED IN MULTIPLE ADVANCED CANCERS 1) | cell, cytoplasm, intracellular, | alcohol metabolism, apoptosis, biopolymer metabolism, biopolymer modification, carbohydrate metabolism, cell adhesion, cell communication, cell cycle, cell death, cell migration, cell motility, cell proliferation, cell-matrix adhesion, cell-substrate adhesion, cellular carbohydrate metabolism, cellular lipid metabolism, cellular macromolecule metabolism, cellular metabolism, cellular physiological process, cellular process, cellular protein metabolism, central nervous system development, death, dephosphorylation, development, establishment of localization, focal adhesion formation, heart development, hexose metabolism, induction of apoptosis, induction of programmed cell death, inositol phosphate dephosphorylation, intracellular signaling cascade, lipid metabolism, lipid modification, localization, localization of cell, locomotion, macromolecule metabolism, metabolism, monosaccharide metabolism, morphogenesis, myo-inositol metabolism, negative regulation of biological process, negative regulation of cell migr | PDZ domain binding, binding, catalytic activity, hydrolase activity, hydrolase activity, acting on ester bonds, inositol or phosphatidylinositol phosphatase activity, inositol-1,3,4,5-tetrakisphosphate 3-phosphatase activity, lipid phosphatase activity, phosphatidylinositol-3,4,5-trisphosphate 3-phosphatase activity, phosphatidylinositol-3,4-bisphosphate 3-phosphatase activity, phosphatidylinositol-3-phosphatase activity, phosphoprotein phosphatase activity, phosphoric ester hydrolase activity, phosphoric monoester hydrolase activity, protein binding, protein domain specific binding, protein serine/threonine phosphatase activity, protein tyrosine phosphatase activity, | active site:Phosphocysteine intermediate, domain:C2 tensin-type, domain:Tyrosine-protein phosphatase, |
| 7803 | PROTEIN TYROSINE PHOSPHATASE TYPE IVA, MEMBER 1 | cell, cytoplasm, endoplasmic reticulum, intracellular, intracellular membrane-bound organelle, intracellular organelle, membrane, membrane-bound organelle, organelle, | biopolymer metabolism, biopolymer modification, cell cycle, cellular macromolecule metabolism, cellular metabolism, cellular physiological process, cellular process, cellular protein metabolism, dephosphorylation, development, macromolecule metabolism, metabolism, phosphate metabolism, phosphorus metabolism, physiological process, primary metabolism, protein amino acid dephosphorylation, protein metabolism, protein modification, | catalytic activity, hydrolase activity, hydrolase activity, acting on ester bonds, phosphoprotein phosphatase activity, phosphoric ester hydrolase activity, phosphoric monoester hydrolase activity, protein tyrosine phosphatase activity, | active site:Phosphocysteine intermediate, active site:Proton donor, binding site:Substrate; phosphate group, disulfide bond, domain:Tyrosine-protein phosphatase, lipid moiety-binding region:S-farnesyl cysteine, mutagenesis site, |
| 7341 | SMT3 SUPPRESSOR OF MIF TWO 3 HOMOLOG 1 (YEAST) | cell, intracellular, intracellular membrane-bound organelle, intracellular organelle, membrane-bound organelle, nucleus, organelle, | biopolymer metabolism, biopolymer modification, cellular macromolecule metabolism, cellular metabolism, cellular physiological process, cellular process, cellular protein metabolism, macromolecule metabolism, metabolism, negative regulation of biological process, negative regulation of cellular metabolism, negative regulation of cellular physiological process, negative regulation of cellular process, negative regulation of metabolism, negative regulation of nucleobase, nucleoside, nucleotide and nucleic acid metabolism, negative regulation of physiological process, negative regulation of transcription, nucleobase, nucleoside, nucleotide and nucleic acid metabolism, physiological process, primary metabolism, protein metabolism, protein modification, protein sumoylation, regulation of biological process, regulation of cellular metabolism, regulation of cellular physiological process, regulation of cellular process, regulation of metabolism, regulation of nucleobase, nucleoside, nucleotide and nucleic acid metab | binding, protein binding, | cross-link:Glycyl lysine isopeptide (Gly-Lys) (interchain with K-? in acceptor proteins), domain:Ubiquitin-like, |
| 55752 | SEPTIN 11 | actin cytoskeleton, cell, cytoskeleton, intracellular, intracellular non-membrane-bound organelle, intracellular organelle, non-membrane-bound organelle, organelle, stress fiber, | cell cycle, cell division, cellular physiological process, cellular process, macromolecule metabolism, metabolism, physiological process, primary metabolism, protein complex assembly, protein heterooligomerization, protein metabolism, protein oligomerization, | GTP binding, binding, guanyl nucleotide binding, nucleotide binding, protein binding, purine nucleotide binding, | modified residue, nucleotide phosphate-binding region:GTP, |
| 5586 | PROTEIN KINASE N2 | cell, intracellular, | biopolymer metabolism, biopolymer modification, cell communication, cellular macromolecule metabolism, cellular metabolism, cellular physiological process, cellular process, cellular protein metabolism, macromolecule metabolism, metabolism, phosphate metabolism, phosphorus metabolism, phosphorylation, physiological process, primary metabolism, protein amino acid phosphorylation, protein metabolism, protein modification, signal transduction, | ATP binding, adenyl nucleotide binding, binding, catalytic activity, kinase activity, nucleotide binding, phosphotransferase activity, alcohol group as acceptor, protein kinase activity, protein serine/threonine kinase activity, purine nucleotide binding, transferase activity, transferase activity, transferring phosphorus-containing groups, | active site:Proton acceptor, binding site:ATP, domain:C2, domain:Protein kinase, nucleotide phosphate-binding region:ATP, repeat:REM 1, repeat:REM 2, repeat:REM 3, |
| 10099 | TETRASPANIN 3 | cell, integral to membrane, intrinsic to membrane, membrane, | cell motility, cell proliferation, cellular physiological process, cellular process, establishment of localization, localization, localization of cell, locomotion, physiological process, | Null | glycosylation site:N-linked (GlcNAc...), transmembrane region, |
| 7373 | COLLAGEN, TYPE XIV, ALPHA 1 (UNDULIN) | FACIT collagen, anchoring collagen, cell, collagen, collagen type XIV, cytoplasm, extracellular matrix, extracellular matrix (sensu Metazoa), extracellular region, intracellular, | anion transport, cell adhesion, cellular physiological process, cellular process, collagen fibril organization, establishment of localization, extracellular matrix organization and biogenesis, extracellular structure organization and biogenesis, inorganic anion transport, ion transport, localization, phosphate transport, physiological process, transport, | binding, extracellular matrix structural constituent, protein binding, structural molecule activity, | domain:Fibronectin type-III 1, domain:Fibronectin type-III 2, domain:Fibronectin type-III 3, domain:Fibronectin type-III 4, domain:Fibronectin type-III 5, domain:Fibronectin type-III 6, domain:Fibronectin type-III 7, domain:Fibronectin type-III 8, domain:TSP N-terminal, domain:VWFA 1, domain:VWFA 2, glycosylation site:N-linked (GlcNAc...), signal peptide, splice variant, |
| 57146 | PROMETHIN | Null | Null | Null | Null |
| 10299 | MEMBRANE-ASSOCIATED RING FINGER (C3HC4) 6 | cell, intracellular, protein complex, ubiquitin ligase complex, | biopolymer metabolism, biopolymer modification, cellular macromolecule metabolism, cellular metabolism, cellular physiological process, cellular process, cellular protein metabolism, macromolecule metabolism, metabolism, physiological process, primary metabolism, protein metabolism, protein modification, protein ubiquitination, ubiquitin cycle, | binding, catalytic activity, cation binding, ion binding, ligase activity, ligase activity, forming carbon-nitrogen bonds, metal ion binding, protein binding, transition metal ion binding, ubiquitin-protein ligase activity, zinc ion binding, | Null |
| 8667 | EUKARYOTIC TRANSLATION INITIATION FACTOR 3, SUBUNIT 3 GAMMA, 40KDA | cell, cytoplasm, eukaryotic 43S preinitiation complex, eukaryotic translation initiation factor 3 complex, intracellular, protein complex, | biosynthesis, cellular biosynthesis, cellular macromolecule metabolism, cellular metabolism, cellular physiological process, cellular process, cellular protein metabolism, macromolecule biosynthesis, macromolecule metabolism, metabolism, physiological process, primary metabolism, protein biosynthesis, protein metabolism, regulation of biological process, regulation of biosynthesis, regulation of cellular biosynthesis, regulation of cellular metabolism, regulation of cellular physiological process, regulation of cellular process, regulation of metabolism, regulation of physiological process, regulation of protein biosynthesis, regulation of protein metabolism, regulation of translation, regulation of translational initiation, translation, translational initiation, | binding, nucleic acid binding, translation factor activity, nucleic acid binding, translation initiation factor activity, translation regulator activity, | Null |
| 8073 | PROTEIN TYROSINE PHOSPHATASE TYPE IVA, MEMBER 2 | cell, membrane, | biopolymer metabolism, biopolymer modification, cellular macromolecule metabolism, cellular metabolism, cellular physiological process, cellular process, cellular protein metabolism, dephosphorylation, macromolecule metabolism, metabolism, phosphate metabolism, phosphorus metabolism, physiological process, primary metabolism, protein amino acid dephosphorylation, protein metabolism, protein modification, | catalytic activity, hydrolase activity, hydrolase activity, acting on ester bonds, phosphoprotein phosphatase activity, phosphoric ester hydrolase activity, phosphoric monoester hydrolase activity, prenylated protein tyrosine phosphatase activity, protein tyrosine phosphatase activity, | active site:Phosphocysteine intermediate, active site:Proton donor, binding site:Substrate; phosphate group, disulfide bond, domain:Tyrosine-protein phosphatase, lipid moiety-binding region:S-farnesyl cysteine, mutagenesis site, splice variant, |
| 4921 | DISCOIDIN DOMAIN RECEPTOR FAMILY, MEMBER 2 | cell, integral to membrane, integral to plasma membrane, intrinsic to membrane, intrinsic to plasma membrane, membrane, plasma membrane, | biopolymer metabolism, biopolymer modification, cell adhesion, cell communication, cell surface receptor linked signal transduction, cellular macromolecule metabolism, cellular metabolism, cellular physiological process, cellular process, cellular protein metabolism, enzyme linked receptor protein signaling pathway, macromolecule metabolism, metabolism, phosphate metabolism, phosphorus metabolism, phosphorylation, physiological process, primary metabolism, protein amino acid phosphorylation, protein metabolism, protein modification, signal transduction, transmembrane receptor protein tyrosine kinase signaling pathway, | ATP binding, adenyl nucleotide binding, binding, catalytic activity, kinase activity, nucleotide binding, phosphotransferase activity, alcohol group as acceptor, protein kinase activity, protein-tyrosine kinase activity, purine nucleotide binding, receptor activity, signal transducer activity, transferase activity, transferase activity, transferring phosphorus-containing groups, transmembrane receptor activity, | active site:Proton acceptor, binding site:ATP, disulfide bond, domain:F5/8 type C, domain:Protein kinase, glycosylation site:N-linked (GlcNAc...), nucleotide phosphate-binding region:ATP, signal peptide, transmembrane region, |
| 2079 | ENHANCER OF RUDIMENTARY HOMOLOG (DROSOPHILA) | Null | cell cycle, cellular metabolism, cellular physiological process, cellular process, metabolism, nucleobase, nucleoside, nucleotide and nucleic acid metabolism, nucleoside metabolism, physiological process, primary metabolism, pyrimidine nucleoside metabolism, regulation of biological process, regulation of cell cycle, regulation of cellular physiological process, regulation of cellular process, regulation of physiological process, regulation of progression through cell cycle, | molecular function unknown, | Null |
| 91851 | CHORDIN-LIKE 1 | Null | development, | Null | domain:VWFC 1, domain:VWFC 2, domain:VWFC 3, glycosylation site:N-linked (GlcNAc...), signal peptide, |
| 3842 | TRANSPORTIN 1 | cell, cytoplasm, endomembrane system, envelope, integral to membrane, intracellular, intracellular membrane-bound organelle, intracellular organelle, intrinsic to membrane, membrane, membrane-bound organelle, nuclear envelope, nuclear pore, nucleus, organelle, organelle envelope, pore complex, protein complex, | cell organization and biogenesis, cellular localization, cellular physiological process, cellular process, establishment of cellular localization, establishment of localization, establishment of protein localization, intracellular protein transport, intracellular transport, localization, nuclear import, nuclear transport, nucleocytoplasmic transport, physiological process, protein import, protein import into nucleus, protein import into nucleus, translocation, protein localization, protein targeting, protein transport, transport, | binding, nuclear localization sequence binding, peptide binding, protein transporter activity, signal sequence binding, transporter activity, | domain:Importin N-terminal, repeat:HEAT 1, repeat:HEAT 2, repeat:HEAT 3, repeat:HEAT 4, repeat:HEAT 5, repeat:HEAT 6, repeat:HEAT 7, repeat:HEAT 8, |
| 8476 | CDC42 BINDING PROTEIN KINASE ALPHA (DMPK-LIKE) | cell, cell junction, intercellular junction, leading edge, membrane, plasma membrane, | actin cytoskeleton organization and biogenesis, actin cytoskeleton reorganization, actin filament-based process, biopolymer metabolism, biopolymer modification, cell communication, cell organization and biogenesis, cellular macromolecule metabolism, cellular metabolism, cellular physiological process, cellular process, cellular protein metabolism, cytoskeleton organization and biogenesis, intracellular signaling cascade, macromolecule metabolism, metabolism, organelle organization and biogenesis, phosphate metabolism, phosphorus metabolism, phosphorylation, physiological process, primary metabolism, protein amino acid phosphorylation, protein metabolism, protein modification, regulation of biological process, regulation of cellular process, regulation of signal transduction, regulation of small GTPase mediated signal transduction, signal transduction, small GTPase mediated signal transduction, | ATP binding, GTPase regulator activity, adenyl nucleotide binding, binding, catalytic activity, cation binding, diacylglycerol binding, enzyme regulator activity, identical protein binding, ion binding, kinase activity, lipid binding, magnesium ion binding, metal ion binding, nucleotide binding, phosphotransferase activity, alcohol group as acceptor, protein binding, protein kinase activity, protein serine/threonine kinase activity, purine nucleotide binding, small GTPase regulator activity, transferase activity, transferase activity, transferring phosphorus-containing groups, transition metal ion binding, zinc ion binding, | active site:Proton acceptor, binding site:ATP, domain:CNH, domain:CRIB, domain:PH, domain:Protein kinase, mutagenesis site, nucleotide phosphate-binding region:ATP, splice variant, zinc finger region:Phorbol-ester/DAG-type, |
| 7707 | ZINC FINGER PROTEIN 148 (PHZ-52) | DNA-directed RNA polymerase II, core complex, DNA-directed RNA polymerase II, holoenzyme, RNA polymerase complex, cell, intracellular, intracellular membrane-bound organelle, intracellular organelle, membrane-bound organelle, membrane-enclosed lumen, nuclear lumen, nucleoplasm, nucleus, organelle, organelle lumen, protein complex, | cellular defense response, cellular metabolism, cellular physiological process, cellular process, defense response, immune response, metabolism, negative regulation of biological process, negative regulation of cellular metabolism, negative regulation of cellular physiological process, negative regulation of cellular process, negative regulation of metabolism, negative regulation of nucleobase, nucleoside, nucleotide and nucleic acid metabolism, negative regulation of physiological process, negative regulation of transcription, negative regulation of transcription from RNA polymerase II promoter, negative regulation of transcription, DNA-dependent, nucleobase, nucleoside, nucleotide and nucleic acid metabolism, organismal physiological process, physiological process, primary metabolism, regulation of biological process, regulation of cellular metabolism, regulation of cellular physiological process, regulation of cellular process, regulation of metabolism, regulation of nucleobase, nucleoside, nucleotide and | DNA binding, RNA polymerase II transcription factor activity, binding, cation binding, ion binding, metal ion binding, nucleic acid binding, specific RNA polymerase II transcription factor activity, transcription regulator activity, transcriptional activator activity, transition metal ion binding, zinc ion binding, | modified residue, zinc finger region:C2H2-type 1, zinc finger region:C2H2-type 2, zinc finger region:C2H2-type 3, zinc finger region:C2H2-type 4, |
| 10559 | SOLUTE CARRIER FAMILY 35 (CMP-SIALIC ACID TRANSPORTER), MEMBER A1 | Golgi apparatus, Golgi membrane, Golgi stack, cell, cytoplasm, endomembrane system, integral to membrane, integral to plasma membrane, intracellular, intracellular membrane-bound organelle, intracellular organelle, intrinsic to membrane, intrinsic to plasma membrane, membrane, membrane-bound organelle, organelle, organelle membrane, plasma membrane, | CMP-sialic acid transport, biopolymer metabolism, biopolymer modification, carbohydrate metabolism, carbohydrate transport, cellular macromolecule metabolism, cellular metabolism, cellular physiological process, cellular process, cellular protein metabolism, establishment of localization, localization, macromolecule metabolism, metabolism, nucleotide-sugar transport, physiological process, primary metabolism, protein metabolism, protein modification, pyrimidine nucleotide-sugar transport, transport, | CMP-sialic acid transporter activity, carbohydrate transporter activity, carrier activity, electrochemical potential-driven transporter activity, nucleotide-sugar transporter activity, porter activity, pyrimidine nucleotide sugar transporter activity, sugar porter activity, sugar transporter activity, transporter activity, | transmembrane region, |
| 9652 | KIAA0372 | cell, cytoplasm, intracellular, intracellular non-membrane-bound organelle, intracellular organelle, non-membrane-bound organelle, organelle, protein complex, ribonucleoprotein complex, ribosome, | biosynthesis, cellular biosynthesis, cellular macromolecule metabolism, cellular metabolism, cellular physiological process, cellular process, cellular protein metabolism, macromolecule biosynthesis, macromolecule metabolism, metabolism, physiological process, primary metabolism, protein biosynthesis, protein metabolism, | binding, structural constituent of ribosome, structural molecule activity, | Null |
| 8337 | HISTONE 2, H2AA | cell, chromatin, chromosome, intracellular, intracellular membrane-bound organelle, intracellular non-membrane-bound organelle, intracellular organelle, membrane-bound organelle, non-membrane-bound organelle, nucleosome, nucleus, organelle, protein complex, | DNA metabolism, DNA packaging, biopolymer metabolism, cell organization and biogenesis, cellular metabolism, cellular physiological process, cellular process, chromatin assembly, chromatin assembly or disassembly, chromosome organization and biogenesis, chromosome organization and biogenesis (sensu Eukaryota), establishment and/or maintenance of chromatin architecture, macromolecule metabolism, metabolism, nucleobase, nucleoside, nucleotide and nucleic acid metabolism, nucleosome assembly, organelle organization and biogenesis, physiological process, primary metabolism, protein complex assembly, protein metabolism, | DNA binding, binding, nucleic acid binding, | Null |
| 10611 | PDZ AND LIM DOMAIN 5 | cell, cell fraction, cytoplasm, cytosol, intracellular, membrane fraction, | development, heart development, morphogenesis, organ development, organ morphogenesis, | actin binding, actinin binding, binding, cation binding, cytoskeletal protein binding, enzyme binding, ion binding, kinase binding, metal ion binding, protein binding, protein kinase C binding, protein kinase binding, receptor signaling complex scaffold activity, signal transducer activity, transition metal ion binding, zinc ion binding, | domain:LIM zinc-binding 1, domain:LIM zinc-binding 2, domain:LIM zinc-binding 3, domain:PDZ, |
| 596 | B-CELL CLL/LYMPHOMA 2 | cell, cytoplasm, endoplasmic reticulum, envelope, integral to membrane, intracellular, intracellular membrane-bound organelle, intracellular organelle, intrinsic to membrane, membrane, membrane-bound organelle, mitochondrial envelope, mitochondrial membrane, mitochondrial outer membrane, mitochondrion, nucleus, organelle, organelle envelope, organelle membrane, organelle outer membrane, outer membrane, | anti-apoptosis, apoptosis, apoptotic mitochondrial changes, apoptotic program, cell cycle, cell death, cell proliferation, cellular physiological process, cellular process, death, defense response, humoral immune response, immune response, negative regulation of apoptosis, negative regulation of biological process, negative regulation of cell proliferation, negative regulation of cellular physiological process, negative regulation of cellular process, negative regulation of physiological process, negative regulation of programmed cell death, organismal physiological process, physiological process, programmed cell death, regulation of apoptosis, regulation of biological process, regulation of cell cycle, regulation of cell proliferation, regulation of cellular physiological process, regulation of cellular process, regulation of physiological process, regulation of programmed cell death, regulation of progression through cell cycle, release of cytochrome c from mitochondria, response to biotic stimulus, respons | binding, identical protein binding, protein binding, | helix, mutagenesis site, site:Cleavage (by caspase-3), splice variant, transmembrane region, |
| 6627 | SMALL NUCLEAR RIBONUCLEOPROTEIN POLYPEPTIDE A' | cell, intracellular, intracellular membrane-bound organelle, intracellular organelle, major (U2-dependent) spliceosome, membrane-bound organelle, nucleus, organelle, protein complex, ribonucleoprotein complex, small nuclear ribonucleoprotein complex, snRNP U2, spliceosome complex, | RNA metabolism, RNA processing, RNA splicing, biopolymer metabolism, cellular metabolism, cellular physiological process, cellular process, macromolecule metabolism, metabolism, nucleobase, nucleoside, nucleotide and nucleic acid metabolism, physiological process, primary metabolism, | RNA binding, binding, nucleic acid binding, | helix, repeat:LRR 1, repeat:LRR 2, repeat:LRR 3, |
| 858 | CAVEOLIN 2 | caveola, caveolar membrane, cell, cytoplasm, integral to membrane, integral to plasma membrane, intracellular, intrinsic to membrane, intrinsic to plasma membrane, lipid raft, membrane, perinuclear region, plasma membrane, | Null | binding, identical protein binding, protein binding, protein dimerization activity, protein homodimerization activity, | modified residue, transmembrane region, |
| 4692 | NECDIN HOMOLOG (MOUSE) | cell, intracellular, intracellular membrane-bound organelle, intracellular organelle, membrane-bound organelle, nucleus, organelle, | cell cycle, cell growth, cell organization and biogenesis, cell proliferation, cellular metabolism, cellular morphogenesis, cellular physiological process, cellular process, development, growth, metabolism, morphogenesis, negative regulation of biological process, negative regulation of cell proliferation, negative regulation of cellular physiological process, negative regulation of cellular process, negative regulation of physiological process, nervous system development, nucleobase, nucleoside, nucleotide and nucleic acid metabolism, physiological process, primary metabolism, regulation of biological process, regulation of cell cycle, regulation of cell growth, regulation of cell proliferation, regulation of cell size, regulation of cellular metabolism, regulation of cellular physiological process, regulation of cellular process, regulation of growth, regulation of metabolism, regulation of nucleobase, nucleoside, nucleotide and nucleic acid metabolism, regulation of physiological process, regulation of pro | DNA binding, binding, nucleic acid binding, | domain:MAGE, |
| 9643 | MORTALITY FACTOR 4 LIKE 2 | cell, intracellular, intracellular membrane-bound organelle, intracellular non-membrane-bound organelle, intracellular organelle, membrane-bound organelle, membrane-enclosed lumen, non-membrane-bound organelle, nuclear lumen, nucleolus, nucleus, organelle, organelle lumen, | DNA metabolism, DNA packaging, biopolymer metabolism, cell growth, cell organization and biogenesis, cellular metabolism, cellular morphogenesis, cellular physiological process, cellular process, chromatin modification, chromosome organization and biogenesis, chromosome organization and biogenesis (sensu Eukaryota), development, establishment and/or maintenance of chromatin architecture, growth, macromolecule metabolism, metabolism, morphogenesis, nucleobase, nucleoside, nucleotide and nucleic acid metabolism, organelle organization and biogenesis, physiological process, primary metabolism, regulation of biological process, regulation of cell growth, regulation of cell size, regulation of cellular metabolism, regulation of cellular physiological process, regulation of cellular process, regulation of growth, regulation of metabolism, regulation of nucleobase, nucleoside, nucleotide and nucleic acid metabolism, regulation of physiological process, regulation of transcription, regulation of transcription, DNA-de | molecular function unknown, | mutagenesis site, |
| 2037 | ERYTHROCYTE MEMBRANE PROTEIN BAND 4.1-LIKE 2 | actin cytoskeleton, cell, cell cortex, cortical actin cytoskeleton, cortical cytoskeleton, cytoplasm, cytoskeleton, intracellular, intracellular non-membrane-bound organelle, intracellular organelle, membrane, non-membrane-bound organelle, organelle, plasma membrane, spectrin, | actin cytoskeleton organization and biogenesis, actin filament-based process, cell organization and biogenesis, cellular physiological process, cellular process, cortical actin cytoskeleton organization and biogenesis, cortical cytoskeleton organization and biogenesis, cytoskeleton organization and biogenesis, organelle organization and biogenesis, physiological process, | actin binding, binding, cytoskeletal protein binding, protein binding, structural constituent of cytoskeleton, structural molecule activity, | domain:FERM, |
| 9749 | PHOSPHATASE AND ACTIN REGULATOR 2 | Null | Null | actin binding, binding, cytoskeletal protein binding, enzyme inhibitor activity, enzyme regulator activity, phosphatase inhibitor activity, phosphatase regulator activity, protein binding, protein phosphatase inhibitor activity, protein phosphatase regulator activity, | repeat:RPEL 1, repeat:RPEL 2, repeat:RPEL 3, repeat:RPEL 4, splice variant, |
| 5502 | PROTEIN PHOSPHATASE 1, REGULATORY (INHIBITOR) SUBUNIT 1A | Null | biopolymer metabolism, carbohydrate metabolism, cell communication, cellular carbohydrate metabolism, cellular macromolecule metabolism, cellular metabolism, cellular physiological process, cellular polysaccharide metabolism, cellular process, generation of precursor metabolites and energy, glucan metabolism, glycogen metabolism, macromolecule metabolism, metabolism, physiological process, polysaccharide metabolism, primary metabolism, signal transduction, | enzyme inhibitor activity, enzyme regulator activity, phosphatase inhibitor activity, phosphatase regulator activity, protein phosphatase inhibitor activity, protein phosphatase regulator activity, | modified residue, mutagenesis site, |
| 9295 | SPLICING FACTOR, ARGININE/SERINE-RICH 11 | cell, intracellular, intracellular membrane-bound organelle, intracellular organelle, membrane-bound organelle, nucleus, organelle, | RNA metabolism, RNA processing, RNA splicing, RNA splicing, via transesterification reactions, RNA splicing, via transesterification reactions with bulged adenosine as nucleophile, biopolymer metabolism, cellular metabolism, cellular physiological process, cellular process, mRNA metabolism, mRNA processing, macromolecule metabolism, metabolism, nuclear mRNA splicing, via spliceosome, nucleobase, nucleoside, nucleotide and nucleic acid metabolism, physiological process, primary metabolism, | RNA binding, binding, nucleic acid binding, nucleotide binding, | domain:RRM, repeat:1, repeat:10, repeat:2, repeat:3, repeat:4, repeat:5, repeat:6, repeat:7, repeat:8, repeat:9, |
| 5178 | PATERNALLY EXPRESSED 3 | cell, intracellular, intracellular membrane-bound organelle, intracellular organelle, membrane-bound organelle, nucleus, organelle, | cellular metabolism, cellular physiological process, cellular process, metabolism, nucleobase, nucleoside, nucleotide and nucleic acid metabolism, physiological process, primary metabolism, regulation of biological process, regulation of cellular metabolism, regulation of cellular physiological process, regulation of cellular process, regulation of metabolism, regulation of nucleobase, nucleoside, nucleotide and nucleic acid metabolism, regulation of physiological process, regulation of transcription, regulation of transcription, DNA-dependent, transcription, transcription, DNA-dependent, | DNA binding, binding, cation binding, ion binding, metal ion binding, nucleic acid binding, transcription factor activity, transcription regulator activity, transition metal ion binding, zinc ion binding, | Null |
| 8082 | SARCOSPAN (KRAS ONCOGENE-ASSOCIATED GENE) | basal lamina, basement membrane, cell, cytoplasm, cytoskeleton, dystrophin-associated glycoprotein complex, extracellular matrix, extracellular matrix (sensu Metazoa), extracellular region, integral to membrane, integral to plasma membrane, intracellular, intracellular non-membrane-bound organelle, intracellular organelle, intrinsic to membrane, intrinsic to plasma membrane, membrane, non-membrane-bound organelle, organelle, plasma membrane, protein complex, | cell adhesion, cellular process, muscle contraction, organismal physiological process, physiological process, | Null | sequence conflict, splice variant, transmembrane region, |
| 56987 | BOBBY SOX HOMOLOG (DROSOPHILA) | Null | cellular metabolism, cellular physiological process, cellular process, metabolism, nucleobase, nucleoside, nucleotide and nucleic acid metabolism, physiological process, primary metabolism, regulation of biological process, regulation of cellular metabolism, regulation of cellular physiological process, regulation of cellular process, regulation of metabolism, regulation of nucleobase, nucleoside, nucleotide and nucleic acid metabolism, regulation of physiological process, regulation of transcription, regulation of transcription, DNA-dependent, transcription, transcription, DNA-dependent, | DNA binding, binding, nucleic acid binding, | Null |
| 351 | AMYLOID BETA (A4) PRECURSOR PROTEIN (PEPTIDASE NEXIN-II, ALZHEIMER DISEASE) | cell, cell surface, coated pit, extracellular region, integral to membrane, integral to plasma membrane, intrinsic to membrane, intrinsic to plasma membrane, membrane, plasma membrane, | Notch signaling pathway, apoptosis, cation homeostasis, cell adhesion, cell communication, cell death, cell homeostasis, cell ion homeostasis, cell surface receptor linked signal transduction, cellular metabolism, cellular physiological process, cellular process, copper ion homeostasis, death, di-, tri-valent inorganic cation homeostasis, electron transport, endocytosis, establishment of localization, generation of precursor metabolites and energy, homeostasis, ion homeostasis, localization, metabolism, metal ion homeostasis, neuromuscular physiological process, neurophysiological process, organismal movement, organismal physiological process, physiological process, programmed cell death, signal transduction, transition metal ion homeostasis, transport, vesicle-mediated transport, | amyloid protein, binding, carbohydrate binding, cation binding, copper ion binding, endopeptidase inhibitor activity, enzyme inhibitor activity, enzyme regulator activity, glycosaminoglycan binding, heme binding, heparin binding, ion binding, iron ion binding, metal ion binding, obsolete molecular function, pattern binding, polysaccharide binding, protease inhibitor activity, protein binding, serine-type endopeptidase inhibitor activity, tetrapyrrole binding, transition metal ion binding, zinc ion binding, | disulfide bond, domain:BPTI/Kunitz inhibitor, glycosylation site:N-linked (GlcNAc...), glycosylation site:O-linked (Xyl...) (chondroitin sulfate); in L-APP isoforms, metal ion-binding site:Copper, metal ion-binding site:Copper or zinc, mutagenesis site, peptide:P3(40), peptide:P3(42), signal peptide, site:Cleavage (by alpha-secretase), site:Cleavage (by beta-secretase), site:Cleavage (by caspase-6; in the AD variant with 670-N-L-671), site:Cleavage (by caspases-6, -8 or -9), site:Cleavage (by gamma-secretase; site 1), site:Cleavage (by gamma-secretase; site 2), site:Cleavage (by gamma-secretase; site 3), site:Implicated in free radical propagation, site:Reactive bond, site:Required for Cu(2+) reduction, site:Susceptible to oxidation, splice variant, transmembrane region, |
| 4929 | NUCLEAR RECEPTOR SUBFAMILY 4, GROUP A, MEMBER 2 | cell, intracellular, intracellular membrane-bound organelle, intracellular organelle, membrane-bound organelle, nucleus, organelle, | antimicrobial humoral response, antimicrobial humoral response (sensu Vertebrata), cell communication, cellular metabolism, cellular physiological process, cellular process, defense response, humoral defense mechanism (sensu Vertebrata), humoral immune response, immune response, metabolism, nucleobase, nucleoside, nucleotide and nucleic acid metabolism, organismal physiological process, physiological process, primary metabolism, regulation of biological process, regulation of cellular metabolism, regulation of cellular physiological process, regulation of cellular process, regulation of metabolism, regulation of nucleobase, nucleoside, nucleotide and nucleic acid metabolism, regulation of physiological process, regulation of transcription, regulation of transcription, DNA-dependent, response to biotic stimulus, response to other organism, response to pest, pathogen or parasite, response to stimulus, response to stress, signal transduction, transcription, transcription, DNA-dependent, | DNA binding, binding, cation binding, ion binding, ligand-dependent nuclear receptor activity, metal ion binding, nucleic acid binding, receptor activity, sequence-specific DNA binding, signal transducer activity, steroid hormone receptor activity, transcription factor activity, transcription regulator activity, transition metal ion binding, zinc ion binding, | DNA-binding region:Nuclear receptor, zinc finger region:NR C4-type, |
| 4026 | LIM DOMAIN CONTAINING PREFERRED TRANSLOCATION PARTNER IN LIPOMA | cell, cellular component unknown, intracellular, intracellular membrane-bound organelle, intracellular organelle, membrane-bound organelle, nucleus, organelle, | biological process unknown, cell adhesion, cellular process, | binding, cation binding, ion binding, metal ion binding, molecular function unknown, protein binding, transition metal ion binding, zinc ion binding, | domain:LIM zinc-binding 1, domain:LIM zinc-binding 2, domain:LIM zinc-binding 3, mutagenesis site, site:Breakpoint for translocation to form HMGA2-LPP, site:Breakpoint for translocation to form HMGA2-LPP and MLL-LPP, |
| 10276 | NEUROEPITHELIAL CELL TRANSFORMING GENE 1 | cell, cellular component unknown, intracellular, intracellular membrane-bound organelle, intracellular organelle, membrane-bound organelle, nucleus, organelle, | cell communication, cell growth, cell organization and biogenesis, cellular morphogenesis, cellular physiological process, cellular process, development, growth, morphogenesis, physiological process, regulation of biological process, regulation of cell growth, regulation of cell size, regulation of cellular physiological process, regulation of cellular process, regulation of growth, regulation of physiological process, signal transduction, | GTPase regulator activity, enzyme regulator activity, guanyl-nucleotide exchange factor activity, small GTPase regulator activity, | domain:DH, domain:PH, splice variant, |
| 7881 | POTASSIUM VOLTAGE-GATED CHANNEL, SHAKER-RELATED SUBFAMILY, BETA MEMBER 1 | cell, cytoplasm, integral to membrane, intracellular, intrinsic to membrane, membrane, | cation transport, cellular physiological process, cellular process, establishment of localization, ion transport, localization, metal ion transport, monovalent inorganic cation transport, physiological process, potassium ion transport, transport, | alkali metal ion binding, alpha-type channel activity, auxiliary transport protein activity, binding, catalytic activity, cation binding, cation transporter activity, channel or pore class transporter activity, ion binding, ion channel activity, ion transporter activity, metal ion binding, oxidoreductase activity, potassium ion binding, transporter activity, voltage-gated ion channel activity, | sequence conflict, splice variant, |
| 22878 | KIAA1012 | Golgi apparatus, Golgi cis-face, Golgi stack, cell, cytoplasm, intracellular, intracellular membrane-bound organelle, intracellular organelle, membrane-bound organelle, organelle, | ER to Golgi vesicle-mediated transport, Golgi vesicle transport, cell organization and biogenesis, cellular localization, cellular physiological process, cellular process, establishment of cellular localization, establishment of localization, intracellular transport, localization, physiological process, secretion, secretory pathway, transport, vesicle-mediated transport, | binding, intracellular transporter activity, transporter activity, | sequence conflict, splice variant, |
| 2908 | NUCLEAR RECEPTOR SUBFAMILY 3, GROUP C, MEMBER 1 (GLUCOCORTICOID RECEPTOR) | cell, cytoplasm, intracellular, intracellular membrane-bound organelle, intracellular organelle, membrane-bound organelle, membrane-enclosed lumen, mitochondrial lumen, mitochondrial matrix, mitochondrion, nucleus, organelle, organelle lumen, | cell communication, cellular metabolism, cellular physiological process, cellular process, defense response, development, immune response, inflammatory response, metabolism, nucleobase, nucleoside, nucleotide and nucleic acid metabolism, organismal physiological process, physiological process, primary metabolism, regulation of biological process, regulation of cellular metabolism, regulation of cellular physiological process, regulation of cellular process, regulation of metabolism, regulation of nucleobase, nucleoside, nucleotide and nucleic acid metabolism, regulation of physiological process, regulation of transcription, regulation of transcription, DNA-dependent, response to biotic stimulus, response to external stimulus, response to other organism, response to pest, pathogen or parasite, response to stimulus, response to stress, response to wounding, sex determination, signal transduction, transcription, transcription from RNA polymerase II promoter, transcription, DNA-dependent, | DNA binding, binding, cation binding, glucocorticoid receptor activity, ion binding, ligand-dependent nuclear receptor activity, lipid binding, metal ion binding, nucleic acid binding, protein binding, receptor activity, sequence-specific DNA binding, signal transducer activity, steroid binding, steroid hormone receptor activity, transcription factor activity, transcription regulator activity, transition metal ion binding, zinc ion binding, | DNA-binding region:Nuclear receptor, cross-link:Glycyl lysine isopeptide (Lys-Gly) (interchain with G-Cter in SUMO), mutagenesis site, splice variant, zinc finger region:NR C4-type, |
| 5928 | RETINOBLASTOMA BINDING PROTEIN 4 | cell, intracellular, intracellular membrane-bound organelle, intracellular organelle, membrane-bound organelle, nucleus, organelle, | DNA metabolism, DNA repair, DNA replication, biopolymer metabolism, cell cycle, cell proliferation, cellular metabolism, cellular physiological process, cellular process, macromolecule metabolism, metabolism, negative regulation of biological process, negative regulation of cell proliferation, negative regulation of cellular physiological process, negative regulation of cellular process, negative regulation of physiological process, nucleobase, nucleoside, nucleotide and nucleic acid metabolism, physiological process, primary metabolism, regulation of biological process, regulation of cell proliferation, regulation of cellular metabolism, regulation of cellular physiological process, regulation of cellular process, regulation of metabolism, regulation of nucleobase, nucleoside, nucleotide and nucleic acid metabolism, regulation of physiological process, regulation of transcription, regulation of transcription, DNA-dependent, response to DNA damage stimulus, response to endogenous stimulus, response to stimulu | binding, protein binding, | modified residue, repeat:WD 1, repeat:WD 2, repeat:WD 3, repeat:WD 4, repeat:WD 5, repeat:WD 6, |
| 285190 | RAN BINDING PROTEIN 2 | cell, endomembrane system, envelope, integral to membrane, intracellular, intracellular membrane-bound organelle, intracellular organelle, intrinsic to membrane, membrane, membrane-bound organelle, nuclear envelope, nuclear pore, nucleus, organelle, organelle envelope, pore complex, protein complex, | cell organization and biogenesis, cellular localization, cellular macromolecule metabolism, cellular metabolism, cellular physiological process, cellular process, cellular protein metabolism, establishment of cellular localization, establishment of localization, establishment of protein localization, intracellular protein transport, intracellular transport, localization, macromolecule metabolism, metabolism, nuclear import, nuclear transport, nucleocytoplasmic transport, physiological process, primary metabolism, protein folding, protein import, protein import into nucleus, protein localization, protein metabolism, protein targeting, protein transport, transport, | GTPase binding, Ran GTPase binding, binding, catalytic activity, cation binding, cis-trans isomerase activity, enzyme binding, ion binding, isomerase activity, metal ion binding, peptidyl-prolyl cis-trans isomerase activity, protein binding, small GTPase binding, transition metal ion binding, zinc ion binding, | domain:PPIase cyclophilin-type, domain:RanBD1 1, domain:RanBD1 2, domain:RanBD1 3, domain:RanBD1 4, repeat:TPR, zinc finger region:RanBP2-type 1, zinc finger region:RanBP2-type 2, zinc finger region:RanBP2-type 3, zinc finger region:RanBP2-type 4, zinc finger region:RanBP2-type 5, zinc finger region:RanBP2-type 6, zinc finger region:RanBP2-type 7, zinc finger region:RanBP2-type 8, |
| 5903 | RAN BINDING PROTEIN 2 | cell, endomembrane system, envelope, integral to membrane, intracellular, intracellular membrane-bound organelle, intracellular organelle, intrinsic to membrane, membrane, membrane-bound organelle, nuclear envelope, nuclear pore, nucleus, organelle, organelle envelope, pore complex, protein complex, | cell organization and biogenesis, cellular localization, cellular macromolecule metabolism, cellular metabolism, cellular physiological process, cellular process, cellular protein metabolism, establishment of cellular localization, establishment of localization, establishment of protein localization, intracellular protein transport, intracellular transport, localization, macromolecule metabolism, metabolism, nuclear import, nuclear transport, nucleocytoplasmic transport, physiological process, primary metabolism, protein folding, protein import, protein import into nucleus, protein localization, protein metabolism, protein targeting, protein transport, transport, | GTPase binding, Ran GTPase binding, binding, catalytic activity, cation binding, cis-trans isomerase activity, enzyme binding, ion binding, isomerase activity, metal ion binding, peptidyl-prolyl cis-trans isomerase activity, protein binding, small GTPase binding, transition metal ion binding, zinc ion binding, | domain:PPIase cyclophilin-type, domain:RanBD1 1, domain:RanBD1 2, domain:RanBD1 3, domain:RanBD1 4, repeat:TPR, zinc finger region:RanBP2-type 1, zinc finger region:RanBP2-type 2, zinc finger region:RanBP2-type 3, zinc finger region:RanBP2-type 4, zinc finger region:RanBP2-type 5, zinc finger region:RanBP2-type 6, zinc finger region:RanBP2-type 7, zinc finger region:RanBP2-type 8, |
| 7536 | SPLICING FACTOR 1 | cell, cytoplasm, intracellular, intracellular membrane-bound organelle, intracellular non-membrane-bound organelle, intracellular organelle, membrane-bound organelle, non-membrane-bound organelle, nucleus, organelle, protein complex, ribonucleoprotein complex, ribosome, spliceosome complex, | RNA metabolism, RNA processing, RNA splicing, RNA splicing, via transesterification reactions, RNA splicing, via transesterification reactions with bulged adenosine as nucleophile, biological process unknown, biopolymer metabolism, cellular metabolism, cellular physiological process, cellular process, mRNA metabolism, mRNA processing, macromolecule metabolism, metabolism, nuclear mRNA splicing, via spliceosome, nucleobase, nucleoside, nucleotide and nucleic acid metabolism, physiological process, primary metabolism, protein complex assembly, protein metabolism, regulation of biological process, regulation of cellular metabolism, regulation of cellular physiological process, regulation of cellular process, regulation of metabolism, regulation of nucleobase, nucleoside, nucleotide and nucleic acid metabolism, regulation of physiological process, regulation of transcription, regulation of transcription, DNA-dependent, spliceosome assembly, transcription, transcription, DNA-dependent, | RNA binding, RNA polymerase II transcription factor activity, binding, cation binding, ion binding, metal ion binding, nucleic acid binding, protein binding, transcription cofactor activity, transcription corepressor activity, transcription factor binding, transcription regulator activity, transition metal ion binding, zinc ion binding, | domain:KH, mutagenesis site, splice variant, zinc finger region:CCHC-type, |
| 4781 | NUCLEAR FACTOR I/B | cell, intracellular, intracellular membrane-bound organelle, intracellular organelle, membrane-bound organelle, nucleus, organelle, | DNA metabolism, DNA replication, biopolymer metabolism, cellular metabolism, cellular physiological process, cellular process, macromolecule metabolism, metabolism, nucleobase, nucleoside, nucleotide and nucleic acid metabolism, physiological process, primary metabolism, regulation of biological process, regulation of cellular metabolism, regulation of cellular physiological process, regulation of cellular process, regulation of metabolism, regulation of nucleobase, nucleoside, nucleotide and nucleic acid metabolism, regulation of physiological process, regulation of transcription, regulation of transcription, DNA-dependent, transcription, transcription, DNA-dependent, | DNA binding, binding, nucleic acid binding, transcription factor activity, transcription regulator activity, | DNA-binding region:CTF/NF-I, splice variant, |
| 477 | ATPASE, NA+/K+ TRANSPORTING, ALPHA 2 (+) POLYPEPTIDE | cell, integral to membrane, integral to plasma membrane, intrinsic to membrane, intrinsic to plasma membrane, membrane, plasma membrane, protein complex, sodium:potassium-exchanging ATPase complex, | cation homeostasis, cation transport, cell homeostasis, cell ion homeostasis, cell motility, cellular physiological process, cellular process, establishment of localization, homeostasis, hydrogen ion homeostasis, hydrogen transport, ion homeostasis, ion transport, localization, localization of cell, locomotion, metabolism, metal ion transport, monovalent inorganic cation homeostasis, monovalent inorganic cation transport, physiological process, potassium ion transport, proton transport, sodium ion transport, sperm motility, transport, | ATP binding, ATPase activity, ATPase activity, coupled, ATPase activity, coupled to movement of substances, ATPase activity, coupled to transmembrane movement of ions, ATPase activity, coupled to transmembrane movement of ions, phosphorylative mechanism, ATPase activity, coupled to transmembrane movement of substances, P-P-bond-hydrolysis-driven transporter activity, adenyl nucleotide binding, alkali metal ion binding, binding, carrier activity, catalytic activity, cation binding, cation transporter activity, hydrolase activity, hydrolase activity, acting on acid anhydrides, hydrolase activity, acting on acid anhydrides, catalyzing transmembrane movement of substances, hydrolase activity, acting on acid anhydrides, in phosphorus-containing anhydrides, ion binding, ion transporter activity, magnesium ion binding, metal ion binding, monovalent inorganic cation transporter activity, nucleoside-triphosphatase activity, nucleotide binding, potassium ion binding, primary active transporter activity, purine nucleoti | active site:4-aspartylphosphate intermediate, metal ion-binding site:Magnesium, transmembrane region, |
| 10512 | SEMA DOMAIN, IMMUNOGLOBULIN DOMAIN (IG), SHORT BASIC DOMAIN, SECRETED, (SEMAPHORIN) 3C | Null | cell communication, cell surface receptor linked signal transduction, cellular process, defense response, development, enzyme linked receptor protein signaling pathway, immune response, organismal physiological process, physiological process, response to abiotic stimulus, response to biotic stimulus, response to chemical stimulus, response to drug, response to stimulus, signal transduction, transmembrane receptor protein tyrosine kinase signaling pathway, | Null | disulfide bond, domain:Ig-like C2-type, domain:Sema, glycosylation site:N-linked (GlcNAc...), signal peptide, |
| 3400 | INHIBITOR OF DNA BINDING 4, DOMINANT NEGATIVE HELIX-LOOP-HELIX PROTEIN | cell, intracellular, intracellular membrane-bound organelle, intracellular organelle, membrane-bound organelle, nucleus, organelle, | cellular metabolism, cellular physiological process, cellular process, metabolism, nucleobase, nucleoside, nucleotide and nucleic acid metabolism, physiological process, primary metabolism, regulation of biological process, regulation of cellular metabolism, regulation of cellular physiological process, regulation of cellular process, regulation of metabolism, regulation of nucleobase, nucleoside, nucleotide and nucleic acid metabolism, regulation of physiological process, regulation of transcription, regulation of transcription from RNA polymerase II promoter, regulation of transcription, DNA-dependent, transcription, transcription from RNA polymerase II promoter, transcription, DNA-dependent, | binding, protein binding, transcription cofactor activity, transcription corepressor activity, transcription factor binding, transcription regulator activity, | domain:Helix-loop-helix motif, |
| 285282 | RAB, MEMBER OF RAS ONCOGENE FAMILY-LIKE 3 | Null | cell communication, cellular metabolism, cellular physiological process, cellular process, intracellular signaling cascade, metabolism, nucleobase, nucleoside, nucleotide and nucleic acid metabolism, physiological process, primary metabolism, regulation of biological process, regulation of cellular metabolism, regulation of cellular physiological process, regulation of cellular process, regulation of metabolism, regulation of nucleobase, nucleoside, nucleotide and nucleic acid metabolism, regulation of physiological process, regulation of transcription, regulation of transcription, DNA-dependent, signal transduction, small GTPase mediated signal transduction, transcription, transcription, DNA-dependent, | ATP binding, DNA binding, GTP binding, adenyl nucleotide binding, binding, guanyl nucleotide binding, nucleic acid binding, nucleotide binding, purine nucleotide binding, | Null |
| 253943 | YTH DOMAIN FAMILY, MEMBER 3 | Null | Null | Null | Null |
| 23215 | BAT2 DOMAIN CONTAINING 1 | Null | Null | Null | Null |
| 3991 | LIPASE, HORMONE-SENSITIVE | Null | alcohol metabolism, biopolymer metabolism, biopolymer modification, carboxylic acid metabolism, catabolism, cellular lipid metabolism, cellular macromolecule metabolism, cellular metabolism, cellular physiological process, cellular process, cellular protein metabolism, cholesterol metabolism, fatty acid metabolism, generation of precursor metabolites and energy, lipid catabolism, lipid metabolism, macromolecule metabolism, metabolism, organic acid metabolism, phosphate metabolism, phosphorus metabolism, phosphorylation, physiological process, primary metabolism, protein amino acid phosphorylation, protein metabolism, protein modification, steroid metabolism, sterol metabolism, | carboxylic ester hydrolase activity, catalytic activity, hydrolase activity, hydrolase activity, acting on ester bonds, lipase activity, | modified residue, splice variant, |
| 5997 | REGULATOR OF G-PROTEIN SIGNALLING 2, 24KDA | Null | G-protein coupled receptor protein signaling pathway, cell communication, cell cycle, cell surface receptor linked signal transduction, cellular physiological process, cellular process, enzyme linked receptor protein signaling pathway, negative regulation of biological process, negative regulation of cellular process, negative regulation of signal transduction, physiological process, regulation of G-protein coupled receptor protein signaling pathway, regulation of biological process, regulation of cellular process, regulation of signal transduction, signal transduction, transmembrane receptor protein tyrosine kinase signaling pathway, | GTPase activator activity, GTPase regulator activity, binding, calmodulin binding, enzyme activator activity, enzyme regulator activity, protein binding, signal transducer activity, | domain:RGS, |
| 10079 | ATPASE, CLASS II, TYPE 9A | cell, integral to membrane, intrinsic to membrane, membrane, | cation transport, cellular physiological process, cellular process, establishment of localization, ion transport, localization, metabolism, physiological process, transport, | ATP binding, ATPase activity, ATPase activity, coupled, ATPase activity, coupled to movement of substances, ATPase activity, coupled to transmembrane movement of ions, ATPase activity, coupled to transmembrane movement of ions, phosphorylative mechanism, ATPase activity, coupled to transmembrane movement of substances, P-P-bond-hydrolysis-driven transporter activity, adenyl nucleotide binding, aminophospholipid transporter activity, binding, carrier activity, catalytic activity, cation transporter activity, hydrolase activity, hydrolase activity, acting on acid anhydrides, hydrolase activity, acting on acid anhydrides, catalyzing transmembrane movement of substances, hydrolase activity, acting on acid anhydrides, in phosphorus-containing anhydrides, ion binding, ion transporter activity, lipid transporter activity, magnesium ion binding, metal ion binding, nucleoside-triphosphatase activity, nucleotide binding, phospholipid transporter activity, phospholipid-translocating ATPase activity, primary active trans | active site:4-aspartylphosphate intermediate, metal ion-binding site:Magnesium, splice variant, transmembrane region, |
| 5955 | RETICULOCALBIN 2, EF-HAND CALCIUM BINDING DOMAIN | cell, cytoplasm, endoplasmic reticulum, intracellular, intracellular membrane-bound organelle, intracellular organelle, membrane-bound organelle, organelle, | Null | binding, calcium ion binding, cation binding, ion binding, metal ion binding, protein binding, | calcium-binding region:1, calcium-binding region:2, calcium-binding region:3; possibly ancestral, calcium-binding region:4, calcium-binding region:5, calcium-binding region:6, domain:EF-hand 1, domain:EF-hand 2, domain:EF-hand 3, domain:EF-hand 4, domain:EF-hand 5, domain:EF-hand 6, signal peptide, |
| 201562 | PROTEIN TYROSINE PHOSPHATASE-LIKE (PROLINE INSTEAD OF CATALYTIC ARGININE), MEMBER B | Null | Null | binding, protein binding, | Null |
| 91746 | YTH DOMAIN CONTAINING 1 | cell, intracellular, intracellular membrane-bound organelle, intracellular organelle, membrane-bound organelle, nucleus, organelle, | RNA metabolism, RNA processing, RNA splicing, RNA splicing, via transesterification reactions, RNA splicing, via transesterification reactions with bulged adenosine as nucleophile, biopolymer metabolism, cellular metabolism, cellular physiological process, cellular process, mRNA metabolism, mRNA processing, macromolecule metabolism, metabolism, nuclear mRNA splicing, via spliceosome, nucleobase, nucleoside, nucleotide and nucleic acid metabolism, physiological process, primary metabolism, | Null | domain:YTH, splice variant, |
| 8019 | BROMODOMAIN CONTAINING 3 | cell, intracellular, intracellular membrane-bound organelle, intracellular organelle, membrane-bound organelle, nucleus, organelle, | biological process unknown, | molecular function unknown, | domain:Bromo 1, domain:Bromo 2, splice variant, |
| 94239 | H2A HISTONE FAMILY, MEMBER V | cell, chromatin, chromosome, intracellular, intracellular membrane-bound organelle, intracellular non-membrane-bound organelle, intracellular organelle, membrane-bound organelle, non-membrane-bound organelle, nucleosome, nucleus, organelle, protein complex, | DNA metabolism, DNA packaging, biopolymer metabolism, cell organization and biogenesis, cellular metabolism, cellular physiological process, cellular process, chromatin assembly, chromatin assembly or disassembly, chromosome organization and biogenesis, chromosome organization and biogenesis (sensu Eukaryota), establishment and/or maintenance of chromatin architecture, macromolecule metabolism, metabolism, nucleobase, nucleoside, nucleotide and nucleic acid metabolism, nucleosome assembly, organelle organization and biogenesis, physiological process, primary metabolism, protein complex assembly, protein metabolism, | DNA binding, binding, nucleic acid binding, | Null |
| 114876 | OXYSTEROL-BINDING PROTEIN-RELATED PROTEIN 1 | cell, intracellular, | alcohol metabolism, cellular lipid metabolism, cellular metabolism, cellular physiological process, cellular process, cholesterol metabolism, establishment of localization, lipid metabolism, lipid transport, localization, metabolism, physiological process, primary metabolism, steroid metabolism, sterol metabolism, transport, vesicle-mediated transport, | binding, lipid binding, phospholipid binding, | zinc finger region:C2H2-type 1, zinc finger region:C2H2-type 2, zinc finger region:C2H2-type 3, |
| 5649 | REELIN | extracellular matrix, extracellular matrix (sensu Metazoa), extracellular region, extracellular space, | cell adhesion, cellular process, development, | binding, catalytic activity, endopeptidase activity, hydrolase activity, peptidase activity, protein binding, serine-type endopeptidase activity, serine-type peptidase activity, | disulfide bond, domain:EGF-like 1, domain:EGF-like 2, domain:EGF-like 3, domain:EGF-like 4, domain:EGF-like 5, domain:EGF-like 6, domain:EGF-like 7, domain:EGF-like 8, domain:Reelin, glycosylation site:N-linked (GlcNAc...), repeat:BNR 1, repeat:BNR 10, repeat:BNR 11, repeat:BNR 12, repeat:BNR 13, repeat:BNR 14, repeat:BNR 15, repeat:BNR 2, repeat:BNR 3, repeat:BNR 4, repeat:BNR 5, repeat:BNR 6, repeat:BNR 7, repeat:BNR 8, repeat:BNR 9, signal peptide, splice variant, |
| 6638 | SMALL NUCLEAR RIBONUCLEOPROTEIN POLYPEPTIDE N | cell, cellular component unknown, intracellular, intracellular membrane-bound organelle, intracellular non-membrane-bound organelle, intracellular organelle, membrane-bound organelle, membrane-enclosed lumen, non-membrane-bound organelle, nuclear lumen, nucleolus, nucleus, organelle, organelle lumen, protein complex, ribonucleoprotein complex, small nucleolar ribonucleoprotein complex, | RNA metabolism, RNA processing, biological process unknown, biopolymer metabolism, cellular metabolism, cellular physiological process, cellular process, mRNA metabolism, mRNA processing, macromolecule metabolism, metabolism, nucleobase, nucleoside, nucleotide and nucleic acid metabolism, physiological process, primary metabolism, | RNA binding, binding, molecular function unknown, nucleic acid binding, | Null |
| 8926 | SMALL NUCLEAR RIBONUCLEOPROTEIN POLYPEPTIDE N | cell, cellular component unknown, intracellular, intracellular membrane-bound organelle, intracellular non-membrane-bound organelle, intracellular organelle, membrane-bound organelle, membrane-enclosed lumen, non-membrane-bound organelle, nuclear lumen, nucleolus, nucleus, organelle, organelle lumen, protein complex, ribonucleoprotein complex, small nucleolar ribonucleoprotein complex, | RNA metabolism, RNA processing, biological process unknown, biopolymer metabolism, cellular metabolism, cellular physiological process, cellular process, mRNA metabolism, mRNA processing, macromolecule metabolism, metabolism, nucleobase, nucleoside, nucleotide and nucleic acid metabolism, physiological process, primary metabolism, | RNA binding, binding, molecular function unknown, nucleic acid binding, | Null |
| 3646 | EUKARYOTIC TRANSLATION INITIATION FACTOR 3, SUBUNIT 6 48KDA | Null | biosynthesis, cellular biosynthesis, cellular macromolecule metabolism, cellular metabolism, cellular physiological process, cellular process, cellular protein metabolism, macromolecule biosynthesis, macromolecule metabolism, metabolism, physiological process, primary metabolism, protein biosynthesis, protein metabolism, | binding, nucleic acid binding, protein binding, translation factor activity, nucleic acid binding, translation initiation factor activity, translation regulator activity, | domain:PCI, |
| 129642 | O-ACYLTRANSFERASE (MEMBRANE BOUND) DOMAIN CONTAINING 2 | Null | Null | Null | Null |
| 1466 | CYSTEINE AND GLYCINE-RICH PROTEIN 2 | cell, intracellular, intracellular membrane-bound organelle, intracellular organelle, membrane-bound organelle, nucleus, organelle, | cell differentiation, cell growth, cell organization and biogenesis, cell proliferation, cellular morphogenesis, cellular physiological process, cellular process, development, growth, morphogenesis, muscle development, organ development, organ morphogenesis, physiological process, regulation of cell size, | binding, cation binding, ion binding, metal ion binding, molecular function unknown, transition metal ion binding, zinc ion binding, | domain:LIM zinc-binding 1, domain:LIM zinc-binding 2, |
| 659 | BONE MORPHOGENETIC PROTEIN RECEPTOR, TYPE II (SERINE/THREONINE KINASE) | cell, integral to membrane, integral to plasma membrane, intrinsic to membrane, intrinsic to plasma membrane, membrane, plasma membrane, | biopolymer metabolism, biopolymer modification, cell communication, cell surface receptor linked signal transduction, cellular macromolecule metabolism, cellular metabolism, cellular physiological process, cellular process, cellular protein metabolism, development, enzyme linked receptor protein signaling pathway, macromolecule metabolism, metabolism, organ development, phosphate metabolism, phosphorus metabolism, phosphorylation, physiological process, primary metabolism, protein amino acid phosphorylation, protein metabolism, protein modification, signal transduction, skeletal development, transmembrane receptor protein serine/threonine kinase signaling pathway, | ATP binding, adenyl nucleotide binding, binding, cAMP-dependent protein kinase activity, catalytic activity, cation binding, cyclic nucleotide-dependent protein kinase activity, ion binding, kinase activity, magnesium ion binding, manganese ion binding, metal ion binding, nucleotide binding, phosphotransferase activity, alcohol group as acceptor, protein binding, protein kinase CK2 activity, protein kinase activity, protein serine/threonine kinase activity, purine nucleotide binding, receptor activity, signal transducer activity, transferase activity, transferase activity, transferring phosphorus-containing groups, transforming growth factor beta receptor activity, transition metal ion binding, transmembrane receptor activity, | active site:Proton acceptor, binding site:ATP, domain:Protein kinase, glycosylation site:N-linked (GlcNAc...), nucleotide phosphate-binding region:ATP, signal peptide, transmembrane region, |
| 2982 | GUANYLATE CYCLASE 1, SOLUBLE, ALPHA 3 | guanylate cyclase complex, soluble, protein complex, unlocalized protein complex, | biosynthesis, cGMP biosynthesis, cGMP metabolism, cell communication, cellular biosynthesis, cellular metabolism, cellular physiological process, cellular process, circulation, cyclic nucleotide biosynthesis, cyclic nucleotide metabolism, intracellular signaling cascade, metabolism, nitric oxide mediated signal transduction, nucleobase, nucleoside, nucleotide and nucleic acid metabolism, nucleotide biosynthesis, nucleotide metabolism, organismal physiological process, physiological process, primary metabolism, signal transduction, | catalytic activity, cyclase activity, guanylate cyclase activity, lyase activity, obsolete molecular function, receptor activity, receptor guanylate cyclase activity, signal transducer activity, | domain:Guanylate cyclase, |
| 4548 | 5-METHYLTETRAHYDROFOLATE-HOMOCYSTEINE METHYLTRANSFERASE | cell, intracellular, | amine biosynthesis, amine metabolism, amino acid and derivative metabolism, amino acid biosynthesis, amino acid metabolism, aromatic compound metabolism, aspartate family amino acid biosynthesis, aspartate family amino acid metabolism, biosynthesis, carboxylic acid metabolism, cellular biosynthesis, cellular metabolism, cellular physiological process, cellular process, central nervous system development, coenzyme biosynthesis, coenzyme metabolism, cofactor biosynthesis, cofactor metabolism, development, folic acid and derivative biosynthesis, folic acid and derivative metabolism, heterocycle metabolism, metabolism, methionine biosynthesis, methionine metabolism, nervous system development, nitrogen compound biosynthesis, nitrogen compound metabolism, organic acid metabolism, physiological process, primary metabolism, sulfur amino acid biosynthesis, sulfur amino acid metabolism, sulfur compound biosynthesis, sulfur metabolism, system development, | 5-methyltetrahydrofolate-dependent methyltransferase activity, S-adenosylmethionine-dependent methyltransferase activity, S-methyltransferase activity, binding, catalytic activity, cation binding, cobalamin binding, cobalt ion binding, dihydropteroate synthase activity, homocysteine S-methyltransferase activity, ion binding, metal ion binding, methionine synthase activity, methyltransferase activity, transferase activity, transferase activity, transferring alkyl or aryl (other than methyl) groups, transferase activity, transferring one-carbon groups, transition metal ion binding, vitamin binding, | domain:AdoMet activation, domain:B12-binding, domain:Hcy-binding, domain:Pterin-binding, metal ion-binding site:Cobalt (cobalamin axial ligand), |
| 5789 | PROTEIN TYROSINE PHOSPHATASE, RECEPTOR TYPE, D | cell, integral to membrane, integral to plasma membrane, intrinsic to membrane, intrinsic to plasma membrane, membrane, plasma membrane, | biopolymer metabolism, biopolymer modification, cell adhesion, cell communication, cell surface receptor linked signal transduction, cellular macromolecule metabolism, cellular metabolism, cellular physiological process, cellular process, cellular protein metabolism, dephosphorylation, enzyme linked receptor protein signaling pathway, macromolecule metabolism, metabolism, phosphate metabolism, phosphorus metabolism, physiological process, primary metabolism, protein amino acid dephosphorylation, protein metabolism, protein modification, signal transduction, transmembrane receptor protein tyrosine phosphatase signaling pathway, | binding, catalytic activity, hydrolase activity, hydrolase activity, acting on ester bonds, phosphoprotein phosphatase activity, phosphoric ester hydrolase activity, phosphoric monoester hydrolase activity, prenylated protein tyrosine phosphatase activity, protein binding, protein tyrosine phosphatase activity, receptor activity, signal transducer activity, transmembrane receptor activity, transmembrane receptor protein phosphatase activity, | active site:Phosphocysteine intermediate, disulfide bond, domain:Fibronectin type-III 1, domain:Fibronectin type-III 2, domain:Fibronectin type-III 3, domain:Fibronectin type-III 4, domain:Fibronectin type-III 5, domain:Fibronectin type-III 6, domain:Fibronectin type-III 7, domain:Fibronectin type-III 8, domain:Ig-like C2-type 1, domain:Ig-like C2-type 2, domain:Ig-like C2-type 3, domain:Tyrosine-protein phosphatase 1, domain:Tyrosine-protein phosphatase 2, glycosylation site:N-linked (GlcNAc...), mutagenesis site, signal peptide, site:Cleavage, splice variant, transmembrane region, |
| 5802 | PROTEIN TYROSINE PHOSPHATASE, RECEPTOR TYPE, D | cell, integral to membrane, integral to plasma membrane, intrinsic to membrane, intrinsic to plasma membrane, membrane, plasma membrane, | biopolymer metabolism, biopolymer modification, cell adhesion, cell communication, cell surface receptor linked signal transduction, cellular macromolecule metabolism, cellular metabolism, cellular physiological process, cellular process, cellular protein metabolism, dephosphorylation, enzyme linked receptor protein signaling pathway, macromolecule metabolism, metabolism, phosphate metabolism, phosphorus metabolism, physiological process, primary metabolism, protein amino acid dephosphorylation, protein metabolism, protein modification, signal transduction, transmembrane receptor protein tyrosine phosphatase signaling pathway, | binding, catalytic activity, hydrolase activity, hydrolase activity, acting on ester bonds, phosphoprotein phosphatase activity, phosphoric ester hydrolase activity, phosphoric monoester hydrolase activity, prenylated protein tyrosine phosphatase activity, protein binding, protein tyrosine phosphatase activity, receptor activity, signal transducer activity, transmembrane receptor activity, transmembrane receptor protein phosphatase activity, | active site:Phosphocysteine intermediate, disulfide bond, domain:Fibronectin type-III 1, domain:Fibronectin type-III 2, domain:Fibronectin type-III 3, domain:Fibronectin type-III 4, domain:Fibronectin type-III 5, domain:Fibronectin type-III 6, domain:Fibronectin type-III 7, domain:Fibronectin type-III 8, domain:Ig-like C2-type 1, domain:Ig-like C2-type 2, domain:Ig-like C2-type 3, domain:Tyrosine-protein phosphatase 1, domain:Tyrosine-protein phosphatase 2, glycosylation site:N-linked (GlcNAc...), mutagenesis site, signal peptide, site:Cleavage, splice variant, transmembrane region, |
| 10771 | ZINC FINGER, MYND DOMAIN CONTAINING 11 | cell, intracellular, intracellular membrane-bound organelle, intracellular organelle, membrane-bound organelle, nucleus, organelle, | cell cycle, cell proliferation, cellular metabolism, cellular physiological process, cellular process, metabolism, negative regulation of biological process, negative regulation of cellular metabolism, negative regulation of cellular physiological process, negative regulation of cellular process, negative regulation of metabolism, negative regulation of nucleobase, nucleoside, nucleotide and nucleic acid metabolism, negative regulation of physiological process, negative regulation of progression through cell cycle, negative regulation of transcription, negative regulation of transcription from RNA polymerase II promoter, negative regulation of transcription, DNA-dependent, nucleobase, nucleoside, nucleotide and nucleic acid metabolism, physiological process, primary metabolism, regulation of biological process, regulation of cell cycle, regulation of cellular metabolism, regulation of cellular physiological process, regulation of cellular process, regulation of metabolism, regulation of nucleobase, nucleoside | DNA binding, binding, cation binding, ion binding, metal ion binding, nucleic acid binding, protein binding, transition metal ion binding, zinc ion binding, | domain:Bromo, domain:PWWP, zinc finger region:MYND-type, zinc finger region:PHD-type, |
| 667 | DYSTONIN | basal part of cell, basal plasma membrane, basement membrane, basolateral plasma membrane, cell, cell junction, cell-matrix junction, cytoplasm, cytoplasmic membrane-bound vesicle, cytoplasmic vesicle, cytoskeleton, extracellular matrix, extracellular matrix (sensu Metazoa), extracellular region, extracellular space, hemidesmosome, intercellular junction, intracellular, intracellular membrane-bound organelle, intracellular non-membrane-bound organelle, intracellular organelle, membrane, membrane-bound organelle, membrane-bound vesicle, non-membrane-bound organelle, organelle, plasma membrane, vesicle, | actin cytoskeleton organization and biogenesis, actin filament-based process, cell adhesion, cell communication, cell cycle, cell cycle arrest, cell organization and biogenesis, cell surface receptor linked signal transduction, cellular physiological process, cellular process, cytoskeleton organization and biogenesis, integrin-mediated signaling pathway, intermediate filament cytoskeleton organization and biogenesis, intermediate filament-based process, negative regulation of biological process, negative regulation of cellular physiological process, negative regulation of cellular process, negative regulation of physiological process, negative regulation of progression through cell cycle, organelle organization and biogenesis, physiological process, regulation of biological process, regulation of cell cycle, regulation of cellular physiological process, regulation of cellular process, regulation of physiological process, regulation of progression through cell cycle, signal transduction, | actin binding, actin filament binding, binding, calcium ion binding, cation binding, cytoskeletal protein binding, integrin binding, ion binding, metal ion binding, protein C-terminus binding, protein binding, receptor binding, signal transducer activity, structural constituent of cytoskeleton, structural molecule activity, | calcium-binding region:1, calcium-binding region:2, domain:Actin-binding, domain:CH 1, domain:CH 2, domain:EF-hand 1, domain:EF-hand 2, domain:SH3, glycosylation site:N-linked (GlcNAc...), repeat:Plectin 1, repeat:Plectin 10, repeat:Plectin 11, repeat:Plectin 2, repeat:Plectin 3, repeat:Plectin 4, repeat:Plectin 5, repeat:Plectin 6, repeat:Plectin 7, repeat:Plectin 8, repeat:Plectin 9, repeat:Spectrin 1, repeat:Spectrin 10, repeat:Spectrin 11, repeat:Spectrin 12, repeat:Spectrin 13, repeat:Spectrin 14, repeat:Spectrin 15, repeat:Spectrin 16, repeat:Spectrin 17, repeat:Spectrin 18, repeat:Spectrin 19, repeat:Spectrin 2, repeat:Spectrin 20, repeat:Spectrin 21, repeat:Spectrin 22, repeat:Spectrin 23, repeat:Spectrin 24, repeat:Spectrin 25, repeat:Spectrin 26, repeat:Spectrin 27, repeat:Spectrin 28, repeat:Spectrin 29, repeat:Spectrin 3, repeat:Spectrin 4, repeat:Spectrin 5, repeat:Spectrin 6, repeat:Spectrin 7, repeat:Spectrin 8, repeat:Spectrin 9, splice variant, |
| 9694 | KIAA0103 | cell, cytoplasm, endoplasmic reticulum, intracellular, intracellular membrane-bound organelle, intracellular organelle, membrane-bound organelle, nucleus, organelle, | Null | binding, | repeat:TPR 1, repeat:TPR 2, repeat:TPR 3, |
| 8780 | RIO KINASE 3 (YEAST) | Null | biopolymer metabolism, biopolymer modification, cellular macromolecule metabolism, cellular metabolism, cellular physiological process, cellular process, cellular protein metabolism, chromosome segregation, macromolecule metabolism, metabolism, phosphate metabolism, phosphorus metabolism, phosphorylation, physiological process, primary metabolism, protein amino acid phosphorylation, protein metabolism, protein modification, | ATP binding, adenyl nucleotide binding, binding, catalytic activity, kinase activity, molecular function unknown, nucleotide binding, phosphotransferase activity, alcohol group as acceptor, protein kinase activity, protein serine/threonine kinase activity, purine nucleotide binding, transferase activity, transferase activity, transferring phosphorus-containing groups, | active site:Proton acceptor, binding site:ATP, splice variant, |
| 5434 | POLYMERASE (RNA) II (DNA DIRECTED) POLYPEPTIDE E, 25KDA | DNA-directed RNA polymerase II, core complex, DNA-directed RNA polymerase II, holoenzyme, RNA polymerase complex, cell, intracellular, intracellular membrane-bound organelle, intracellular organelle, membrane-bound organelle, membrane-enclosed lumen, nuclear lumen, nucleoplasm, nucleus, organelle, organelle lumen, protein complex, | cellular metabolism, cellular physiological process, cellular process, metabolism, nucleobase, nucleoside, nucleotide and nucleic acid metabolism, physiological process, primary metabolism, transcription, transcription from RNA polymerase II promoter, transcription, DNA-dependent, | DNA binding, DNA-directed RNA polymerase I activity, DNA-directed RNA polymerase II activity, DNA-directed RNA polymerase III activity, DNA-directed RNA polymerase activity, binding, catalytic activity, nucleic acid binding, nucleotidyltransferase activity, obsolete molecular function, protein binding, transferase activity, transferase activity, transferring phosphorus-containing groups, | Null |
| 2186 | FETAL ALZHEIMER ANTIGEN | cell, cytoplasm, intracellular, intracellular membrane-bound organelle, intracellular organelle, membrane-bound organelle, nucleus, organelle, | cellular metabolism, cellular physiological process, cellular process, development, metabolism, negative regulation of biological process, negative regulation of cellular metabolism, negative regulation of cellular physiological process, negative regulation of cellular process, negative regulation of metabolism, negative regulation of nucleobase, nucleoside, nucleotide and nucleic acid metabolism, negative regulation of physiological process, negative regulation of transcription, negative regulation of transcription from RNA polymerase II promoter, negative regulation of transcription, DNA-dependent, nervous system development, nucleobase, nucleoside, nucleotide and nucleic acid metabolism, physiological process, primary metabolism, regulation of biological process, regulation of cellular metabolism, regulation of cellular physiological process, regulation of cellular process, regulation of metabolism, regulation of nucleobase, nucleoside, nucleotide and nucleic acid metabolism, regulation of physiological pr | binding, cation binding, ion binding, metal ion binding, protein binding, transcription factor binding, transcription regulator activity, transition metal ion binding, zinc ion binding, | domain:DDT, zinc finger region:PHD-type, |
| 56969 | SPECTRIN, BETA, NON-ERYTHROCYTIC 1 | actin cytoskeleton, cell, cell cortex, cortical actin cytoskeleton, cortical cytoskeleton, cytoplasm, cytoskeleton, intracellular, intracellular non-membrane-bound organelle, intracellular organelle, membrane, non-membrane-bound organelle, organelle, spectrin, | actin cytoskeleton organization and biogenesis, actin filament capping, actin filament depolymerization, actin filament-based process, actin polymerization and/or depolymerization, barbed-end actin filament capping, cell organization and biogenesis, cellular macromolecule metabolism, cellular metabolism, cellular physiological process, cellular process, cellular protein metabolism, cytoskeleton organization and biogenesis, macromolecule metabolism, metabolism, negative regulation of actin filament depolymerization, negative regulation of biological process, negative regulation of cell organization and biogenesis, negative regulation of cellular physiological process, negative regulation of cellular process, negative regulation of metabolism, negative regulation of physiological process, negative regulation of protein metabolism, organelle organization and biogenesis, physiological process, primary metabolism, protein depolymerization, protein metabolism, regulation of actin filament depolymerization, regulati | actin binding, binding, calmodulin binding, cytoskeletal protein binding, protein binding, structural constituent of cytoskeleton, structural molecule activity, | domain:Actin-binding, domain:CH 1, domain:CH 2, domain:PH, repeat:Spectrin 1, repeat:Spectrin 10, repeat:Spectrin 11, repeat:Spectrin 12, repeat:Spectrin 13, repeat:Spectrin 14, repeat:Spectrin 15, repeat:Spectrin 16, repeat:Spectrin 17, repeat:Spectrin 2, repeat:Spectrin 3, repeat:Spectrin 4, repeat:Spectrin 5, repeat:Spectrin 6, repeat:Spectrin 7, repeat:Spectrin 8, repeat:Spectrin 9, splice variant, |
| 6711 | SPECTRIN, BETA, NON-ERYTHROCYTIC 1 | actin cytoskeleton, cell, cell cortex, cortical actin cytoskeleton, cortical cytoskeleton, cytoplasm, cytoskeleton, intracellular, intracellular non-membrane-bound organelle, intracellular organelle, membrane, non-membrane-bound organelle, organelle, spectrin, | actin cytoskeleton organization and biogenesis, actin filament capping, actin filament depolymerization, actin filament-based process, actin polymerization and/or depolymerization, barbed-end actin filament capping, cell organization and biogenesis, cellular macromolecule metabolism, cellular metabolism, cellular physiological process, cellular process, cellular protein metabolism, cytoskeleton organization and biogenesis, macromolecule metabolism, metabolism, negative regulation of actin filament depolymerization, negative regulation of biological process, negative regulation of cell organization and biogenesis, negative regulation of cellular physiological process, negative regulation of cellular process, negative regulation of metabolism, negative regulation of physiological process, negative regulation of protein metabolism, organelle organization and biogenesis, physiological process, primary metabolism, protein depolymerization, protein metabolism, regulation of actin filament depolymerization, regulati | actin binding, binding, calmodulin binding, cytoskeletal protein binding, protein binding, structural constituent of cytoskeleton, structural molecule activity, | domain:Actin-binding, domain:CH 1, domain:CH 2, domain:PH, repeat:Spectrin 1, repeat:Spectrin 10, repeat:Spectrin 11, repeat:Spectrin 12, repeat:Spectrin 13, repeat:Spectrin 14, repeat:Spectrin 15, repeat:Spectrin 16, repeat:Spectrin 17, repeat:Spectrin 2, repeat:Spectrin 3, repeat:Spectrin 4, repeat:Spectrin 5, repeat:Spectrin 6, repeat:Spectrin 7, repeat:Spectrin 8, repeat:Spectrin 9, splice variant, |
| 4925 | NUCLEOBINDIN 2 | ER-Golgi intermediate compartment, cell, cytoplasm, cytosol, extracellular region, extracellular space, intracellular, membrane, plasma membrane, | Null | DNA binding, binding, calcium ion binding, cation binding, ion binding, metal ion binding, nucleic acid binding, | calcium-binding region:1, calcium-binding region:2, domain:EF-hand 1, domain:EF-hand 2, signal peptide, |
| 23008 | KIAA0265 PROTEIN | Null | Null | Null | Null |
| 4659 | PROTEIN PHOSPHATASE 1, REGULATORY (INHIBITOR) SUBUNIT 12A | actin cytoskeleton, cell, cytoskeleton, intracellular, intracellular non-membrane-bound organelle, intracellular organelle, non-membrane-bound organelle, organelle, | muscle contraction, organismal physiological process, physiological process, regulation of biological process, regulation of muscle contraction, regulation of organismal physiological process, regulation of physiological process, | signal transducer activity, | modified residue, repeat:ANK 1, repeat:ANK 2, repeat:ANK 3, repeat:ANK 4, repeat:ANK 5, repeat:ANK 6, splice variant, |
| 6285 | S100 CALCIUM BINDING PROTEIN, BETA (NEURAL) | cell, cytoplasm, extracellular region, intracellular, intracellular membrane-bound organelle, intracellular organelle, membrane-bound organelle, nucleus, organelle, | apoptosis, astrocyte activation, axonogenesis, behavior, biosynthesis, calcium ion homeostasis, cation homeostasis, cell activation, cell communication, cell death, cell development, cell differentiation, cell homeostasis, cell ion homeostasis, cell organization and biogenesis, cell proliferation, cell-cell signaling, cellular biosynthesis, cellular macromolecule metabolism, cellular metabolism, cellular morphogenesis, cellular morphogenesis during differentiation, cellular physiological process, cellular process, cellular protein metabolism, central nervous system development, complement activation, cytokine biosynthesis, cytokine metabolism, cytokine production, death, defense response, development, di-, tri-valent inorganic cation homeostasis, generation of precursor metabolites and energy, homeostasis, humoral immune response, hyperphosphorylation, immune cell activation, immune response, induction of apoptosis, induction of programmed cell death, ion homeostasis, learning and/or memory, macromolecule bio | S100 alpha binding, S100 beta binding, binding, calcium ion binding, cation binding, cytoskeletal protein binding, enzyme inhibitor activity, enzyme regulator activity, identical protein binding, ion binding, kinase inhibitor activity, kinase regulator activity, metal ion binding, protein binding, protein dimerization activity, protein homodimerization activity, tau protein binding, transition metal ion binding, zinc ion binding, | calcium-binding region:1; low affinity, calcium-binding region:2; high affinity, domain:EF-hand 1, domain:EF-hand 2, |
| 7103 | TETRASPANIN 8 | cell, cytoplasm, integral to membrane, integral to plasma membrane, intracellular, intracellular membrane-bound organelle, intracellular organelle, intrinsic to membrane, intrinsic to plasma membrane, lysosome, lytic vacuole, membrane, membrane-bound organelle, organelle, plasma membrane, vacuole, | biopolymer glycosylation, biopolymer metabolism, biopolymer modification, biosynthesis, cellular biosynthesis, cellular macromolecule metabolism, cellular metabolism, cellular physiological process, cellular process, cellular protein metabolism, glycoprotein biosynthesis, glycoprotein metabolism, macromolecule biosynthesis, macromolecule metabolism, metabolism, physiological process, primary metabolism, protein amino acid glycosylation, protein biosynthesis, protein metabolism, protein modification, | signal transducer activity, | glycosylation site:N-linked (GlcNAc...), transmembrane region, |
| 10659 | CUG TRIPLET REPEAT, RNA BINDING PROTEIN 2 | Null | RNA metabolism, RNA processing, biopolymer metabolism, cellular metabolism, cellular physiological process, cellular process, circulation, macromolecule metabolism, metabolism, nucleobase, nucleoside, nucleotide and nucleic acid metabolism, organismal physiological process, physiological process, primary metabolism, regulation of biological process, regulation of heart contraction, regulation of organismal physiological process, regulation of physiological process, | RNA binding, binding, nucleic acid binding, nucleotide binding, | Null |
| 59 | ACTIN, ALPHA 2, SMOOTH MUSCLE, AORTA | actin cytoskeleton, actin filament, cell, cytoskeleton, intracellular, intracellular non-membrane-bound organelle, intracellular organelle, non-membrane-bound organelle, organelle, | muscle contraction, organismal physiological process, physiological process, | ATP binding, adenyl nucleotide binding, binding, nucleotide binding, protein binding, purine nucleotide binding, structural constituent of cytoskeleton, structural molecule activity, | modified residue, propeptide:Removed in mature form, |
| 72 | ACTIN, ALPHA 2, SMOOTH MUSCLE, AORTA | actin cytoskeleton, actin filament, cell, cytoskeleton, intracellular, intracellular non-membrane-bound organelle, intracellular organelle, non-membrane-bound organelle, organelle, | muscle contraction, organismal physiological process, physiological process, | ATP binding, adenyl nucleotide binding, binding, nucleotide binding, protein binding, purine nucleotide binding, structural constituent of cytoskeleton, structural molecule activity, | modified residue, propeptide:Removed in mature form, |
| 1805 | DERMATOPONTIN | extracellular matrix, extracellular matrix (sensu Metazoa), extracellular region, | cell adhesion, cellular process, | binding, protein binding, | disulfide bond, repeat:1-1, repeat:1-2, repeat:2-1, repeat:2-2, repeat:3-3, signal peptide, |
| 2167 | FATTY ACID BINDING PROTEIN 4, ADIPOCYTE | cell, cell fraction, cytoplasm, intracellular, soluble fraction, | cellular physiological process, cellular process, establishment of localization, lipid binding, localization, obsolete biological process, physiological process, transport, | binding, fatty acid binding, lipid binding, | Null |
| 146057 | KIAA0847 PROTEIN | cell, cytoskeleton, intermediate filament, intermediate filament cytoskeleton, intracellular, intracellular non-membrane-bound organelle, intracellular organelle, non-membrane-bound organelle, organelle, | biopolymer metabolism, biopolymer modification, cellular macromolecule metabolism, cellular metabolism, cellular physiological process, cellular process, cellular protein metabolism, macromolecule metabolism, metabolism, phosphate metabolism, phosphorus metabolism, phosphorylation, physiological process, primary metabolism, protein amino acid phosphorylation, protein metabolism, protein modification, | ATP binding, adenyl nucleotide binding, binding, catalytic activity, kinase activity, nucleotide binding, phosphotransferase activity, alcohol group as acceptor, protein kinase activity, purine nucleotide binding, structural molecule activity, transferase activity, transferase activity, transferring phosphorus-containing groups, | Null |
| 427 | N-ACYLSPHINGOSINE AMIDOHYDROLASE (ACID CERAMIDASE) 1 | cell, cytoplasm, intracellular, intracellular membrane-bound organelle, intracellular organelle, lysosome, lytic vacuole, membrane-bound organelle, organelle, vacuole, | carboxylic acid metabolism, cellular lipid metabolism, cellular metabolism, cellular physiological process, cellular process, ceramide metabolism, fatty acid metabolism, lipid metabolism, membrane lipid metabolism, metabolism, organic acid metabolism, physiological process, primary metabolism, sphingoid metabolism, sphingolipid metabolism, | catalytic activity, ceramidase activity, hydrolase activity, hydrolase activity, acting on carbon-nitrogen (but not peptide) bonds, hydrolase activity, acting on carbon-nitrogen (but not peptide) bonds, in linear amides, transferase activity, transferase activity, transferring acyl groups, transferase activity, transferring acyl groups, acyl groups converted into alkyl on transfer, | glycosylation site:N-linked (GlcNAc...), signal peptide, |
| 29896 | TRANSFORMER-2 ALPHA | cell, intracellular, intracellular membrane-bound organelle, intracellular organelle, membrane-bound organelle, nucleus, organelle, | RNA metabolism, RNA processing, RNA splicing, RNA splicing, via transesterification reactions, RNA splicing, via transesterification reactions with bulged adenosine as nucleophile, biopolymer metabolism, cellular metabolism, cellular physiological process, cellular process, mRNA metabolism, mRNA processing, macromolecule metabolism, metabolism, nuclear mRNA splicing, via spliceosome, nucleobase, nucleoside, nucleotide and nucleic acid metabolism, physiological process, primary metabolism, | RNA binding, binding, nucleic acid binding, nucleotide binding, | domain:RRM, splice variant, |
| 5495 | PROTEIN PHOSPHATASE 1B (FORMERLY 2C), MAGNESIUM-DEPENDENT, BETA ISOFORM | protein complex, protein serine/threonine phosphatase complex, unlocalized protein complex, | biopolymer metabolism, biopolymer modification, cellular macromolecule metabolism, cellular metabolism, cellular physiological process, cellular process, cellular protein metabolism, dephosphorylation, macromolecule metabolism, metabolism, phosphate metabolism, phosphorus metabolism, physiological process, primary metabolism, protein amino acid dephosphorylation, protein metabolism, protein modification, | CTD phosphatase activity, binding, calcium-dependent protein serine/threonine phosphatase activity, catalytic activity, cation binding, hydrolase activity, hydrolase activity, acting on ester bonds, ion binding, magnesium ion binding, magnesium-dependent protein serine/threonine phosphatase activity, manganese ion binding, metal ion binding, myosin phosphatase activity, phosphoprotein phosphatase activity, phosphoric ester hydrolase activity, phosphoric monoester hydrolase activity, protein phosphatase type 1 activity, protein phosphatase type 2A activity, protein phosphatase type 2B activity, protein phosphatase type 2C activity, protein serine/threonine phosphatase activity, transition metal ion binding, | metal ion-binding site:Manganese 1, metal ion-binding site:Manganese 1 (via carbonyl oxygen), metal ion-binding site:Manganese 2, splice variant, |
| 9852 | EPM2A (LAFORIN) INTERACTING PROTEIN 1 | cell, cytoplasm, endoplasmic reticulum, intracellular, intracellular membrane-bound organelle, intracellular organelle, membrane-bound organelle, organelle, | Null | Null | splice variant, |
| 4915 | NEUROTROPHIC TYROSINE KINASE, RECEPTOR, TYPE 2 | cell, integral to membrane, integral to plasma membrane, intrinsic to membrane, intrinsic to plasma membrane, membrane, plasma membrane, | biopolymer metabolism, biopolymer modification, cell communication, cell differentiation, cell surface receptor linked signal transduction, cellular macromolecule metabolism, cellular metabolism, cellular physiological process, cellular process, cellular protein metabolism, development, enzyme linked receptor protein signaling pathway, macromolecule metabolism, metabolism, nervous system development, phosphate metabolism, phosphorus metabolism, phosphorylation, physiological process, primary metabolism, protein amino acid phosphorylation, protein metabolism, protein modification, signal transduction, system development, transmembrane receptor protein tyrosine kinase signaling pathway, | ATP binding, adenyl nucleotide binding, binding, catalytic activity, growth factor binding, kinase activity, neurotrophin binding, nucleotide binding, phosphotransferase activity, alcohol group as acceptor, protein binding, protein kinase activity, protein-tyrosine kinase activity, purine nucleotide binding, receptor activity, signal transducer activity, transferase activity, transferase activity, transferring phosphorus-containing groups, transmembrane receptor activity, | active site:Proton acceptor, binding site:ATP, disulfide bond, domain:Ig-like C2-type 1, domain:Ig-like C2-type 2, domain:Protein kinase, glycosylation site:N-linked (GlcNAc...), nucleotide phosphate-binding region:ATP, repeat:LRR 1, repeat:LRR 2, signal peptide, site:Interaction with PLC-gamma-1, site:Interaction with SHC1, splice variant, transmembrane region, |

**Additional Table S3**

**Table S3.** List of interacting elements for the visualization of the network in Figure 4a. Interactions were retrieved from the Biomolecular Interaction Network Database (BIND), the Biological General Repository for Interaction Datasets (BioGRID) and literature. Gene A is the first element of the interaction and Gene B the second one while interactions type are divided in protein-protein interactions (pp), interactions with ions (interactsWith), interaction between proteins and DNA (pd), literature inferred (DefaultEdge) and DirectedEdge sustain a direct interaction.

| **Gene A** | **Interaction type** | **Gene B** |
| --- | --- | --- |
| LIPE | (DefaultEdge) | AR |
| AR | (pp) | GSN |
| S100A11 | (pp) | S100A11 |
| HNF4-alpha | (pd) | RAB1B |
| TLE1 | (pp) | TLE1 |
| SHC1 | (pp) | PPP2R5A |
| S100A9 | (pp) | S100A8 |
| PPP1CB | (pp) | PPP1R12A |
| DCN | (pp) | COL14A1 |
| EIF3S10 | (pp) | EIF3S3 |
| Ca2+ | (interactsWith) | S100A9 |
| SRC | (pp) | SRC |
| BNIP3 | (DefaultEdge) | BNIP3 |
| BCL2 | (pp) | NR4A1 |
| TNF | (DirectedEdge) | ICOSLG |
| Histone_2a_Peptide | (pp) | HLA-G |
| TSG101 | (pp) | NR3C1 |
| SRC | (pp) | MPZL1 |
| PTP4A2 | (pp) | PTP4A1 |
| Smad8 | (pp) | GRN |
| PPP3CA | (pp) | FKBP8 |
| SF1 | (pp) | RBPMS |
| SAP18 | (pp) | EIF3S6 |
| HNF4-alpha | (pd) | PPP1R15B |
| HCK | (pp) | HCK |
| CTF1 | (DirectedEdge) | SHC1 |
| CHI3L1 | (pp) | CHI3L1 |
| NDN | (pp) | RPS11 |
| HNF6 | (pd) | ACVR1 |
| HSF1 | (pp) | STAT1 |
| AHNAK | (pp) | S100B |
| BCL2 | (pp) | PPP3CA |
| HNF4-alpha | (pd) | STAT1 |
| PTEN | (pp) | ESR1 |
| STAT1 | (pp) | PLAUR |
| NDN | (pp) | NUCB2 |
| vinculin | (pp) | B2M |
| HNF1-alpha | (pd) | LY6E |
| paxillin | (pp) | MVP |
| PBX1 | (pp) | NR3C1 |
| ZBTB16 | (pp) | ESR1 |
| Smad4 | (pp) | HYPA |
| BSG | (DirectedEdge) | APP |
| Source | (interaction) | Target |
| TRG20 | (pp) | TRG20 |
| NR3C1 | (pp) | TSG101 |
| CCL5 | (pp) | SDC4 |
| HEM | (interactsWith) | HMOX1 |
| NR3C1 | (pp) | TXN |
| HSF1 | (pp) | TAF7 |
| NFIB | (pp) | NFIC |
| HYPA | (pp) | ZAP3 |
| Tat | (pp) | B2M |
| vinculin | (pp) | SERPINH1 |
| S100A11 | (pp) | S100B |
| IL5 | (DirectedEdge) | SHC1 |
| talin | (pp) | FMNL1 |
| S100A8 | (pp) | S100A8 |
| PIAS1 | (pp) | STAT1 |
| SARA | (pp) | Smad2 |
| TGF-beta-RI | (pp) | STAT1 |
| Smad2 | (pp) | SMURF1 |
| ANXA6 | (pp) | S100B |
| PLAUR | (pp) | PLAUR |
| Vif | (pp) | HCK |
| APP | (pp) | APP |
| CDK6 | (pp) | PPM1B |
| MOAP1 | (pp) | BCL2 |
| CCL3 | (pp) | CCL4 |
| SMAD3 | (pp) | NR3C1 |
| DNAJB1 | (pp) | HSPA1A |
| FYN | (pp) | NTRK2 |
| p621 | (pp) | HEY-L |
| DNAJB1 | (DirectedEdge) | HMOX1 |
| SF1 | (pp) | BCL2 |
| BAG1 | (pp) | AR |
| CDK6 | (pp) | CDK6 |
| VEGF | (pp) | VEGF |
| Smad8 | (pp) | MGAT1 |
| NR2F6 | (pp) | NR3C1 |
| APP | (pp) | FBLN1 |
| AR | (pp) | NCOA3 |
| TP53 | (pp) | TP53 |
| Tat | (pp) | APOE |
| RARA | (pp) | RARA |
| NR3C1 | (pp) | NR3C1 |
| PGM1 | (pp) | S100B |
| RGS2 | (pp) | COPB |
| NR3C1 | (pp) | SELENBP1 |
| COPB | (pp) | RGS2 |
| ESR1 | (pp) | NCOA3 |
| VAV1 | (pp) | S100B |
| ZBTB16 | (pp) | ZBTB16 |
| HDAC9 | (pp) | ZBTB16 |
| STAT1 | (DirectedEdge) | KLF2 |
| Smad4 | (pp) | p621 |
| IL10 | (pp) | IL10 |
| BCL2 | (pp) | ITPR1 |
| ZBTB16 | (pp) | NR3C1 |
| paxillin | (pp) | SERPINH1 |
| NCOA3 | (pp) | AR |
| HNF6 | (pd) | HLA-G |
| RGPD5 | (pp) | TNPO1 |
| APP | (pp) | CAV1 |
| HNRPU | (pp) | NR3C1 |
| NAG-(1-4)NAG-(1-4)NAG-(1-4)NAG | (interactsWith) | CHI3L1 |
| CD36 | (DefaultEdge) | CAV1 |
| HSF1 | (pp) | HSPA1A |
| TP53BP2 | (pp) | TP53 |
| Tat | (pp) | IL10 |
| HNF4-alpha | (pd) | S100A9 |
| TNF | (pp) | TNFRSF1A |
| ESR1 | (pp) | BAG1 |
| HNF4-alpha | (pd) | NAG |
| NCOA3 | (pp) | NR4A1 |
| RUSC2 | (pp) | GOLGA2 |
| S100B | (pp) | S100B |
| VEGF | (pp) | GPC1 |
| Unknown | (interactsWith) | SERPINH1 |
| Unknown | (interactsWith) | TP53BP2 |
| PTPRS | (pp) | PTPRD |
| HYPA | (pp) | HYPA |
| NR3C1 | (pp) | BAG1 |
| Tat | (pp) | HLA-G |
| AR | (pp) | TGFB1I1 |
| FTL | (pp) | FTL |
| AKR1C1 | (pp) | AKR1C1 |
| MX1 | (pp) | MX1 |
| ACVR1 | (pp) | STAT1 |
| LRP1 | (pp) | SERPINE1 |
| AR | (pp) | CAV1 |
| Smad4 | (pp) | Smad2 |
| Tat | (pp) | GRN |
| SMAD4 | (pp) | STAT1 |
| DNAJA1 | (DirectedEdge) | DNAJB1 |
| RNPS1 | (pp) | SRRM2 |
| NAG-(1-4)NAG-(1-4)NAG | (interactsWith) | CHI3L1 |
| CAV1 | (pp) | AR |
| SFRS1 | (pp) | SFRS11 |
| Unknown | (interactsWith) | vinculin |
| Annexin_I | (pp) | S100A11 |
| SMURF1 | (pp) | STAT1 |
| FYN | (pp) | FYN |
| 1LYB_I | (pp) | CTSD |
| SRC | (pp) | STAT5A |
| FCGRT | (pp) | FCGRT |
| Quercetin | (interactsWith) | HCK |
| IL5 | (pp) | IL5 |
| TNF | (DirectedEdge) | FTL |
| ANXA11 | (DirectedEdge) | Annexin_I |
| Unknown | (interactsWith) | KRT14 |
| BMPR2 | (DefaultEdge) | CAV2 |
| BCL6 | (pp) | JUND |
| DNAJB1 | (pp) | HSF1 |
| CSNK2A2 | (pp) | FGF2 |
| ERBB2IP | (pp) | SMAD3 |
| SNRPN | (pp) | FBL |
| BNIP3 | (pp) | BCL2 |
| 1BF5_C | (pd) | STAT1 |
| HNF4-alpha | (pd) | AF15Q14 |
| APP | (pp) | APOE |
| STAT1 | (pp) | STAT2 |
| RIOK3 | (pp) | FBL |
| IL7 | (DirectedEdge) | SHC1 |
| BNIP3L | (pp) | BNIP3L |
| Unknown | (interactsWith) | Vif |
| DES | (pp) | S100B |
| NAG-(4-1)NAG-(4-1)MAN-(6-1)MAN | (interactsWith) | CTSD |
| PTEN | (pp) | AR |
| EXT2 | (pp) | EXT2 |
| POU2F1 | (pp) | NR3C1 |
| MORF4L2 | (pp) | HDAC9 |
| IFNG | (DirectedEdge) | MX1 |
| IL10RA | (pp) | IL10 |
| B2M | (pp) | FCGRT |
| HNF4-alpha | (pd) | ATM |
| Unknown | (interactsWith) | GSTM1 |
| Tat | (pp) | PPP1R8 |
| JUND | (pp) | FOSL2 |
| Interleukin-10-Like_Protein | (pp) | IL10RA |
| TNF | (pp) | TNF |
| NR2F6 | (pp) | ESR1 |
| HNF4-alpha | (pd) | PPP1R10 |
| EIF3S10 | (pp) | EIF3S4 |
| NSD1 | (pp) | RARA |
| ERBB2IP | (pp) | DST |
| IL8 | (pp) | CCL4 |
| TNFRSF1A | (pp) | TRAF1 |
| TNFRSF1A | (pp) | TNF |
| TSG101 | (pp) | TSG101 |
| AR | (pp) | NR3C1 |
| Unknown | (interactsWith) | talin |
| BNIP3 | (pp) | BNIP3L |
| CCL4 | (pp) | CCL4 |
| HNF4-alpha | (pd) | ABCC3 |
| TNPO1 | (pp) | HIST2H2BE |
| AR | (pp) | TSG101 |
| HSF1 | (pp) | DNAJB1 |
| PPM1B | (pp) | GSN |
| APP | (pp) | GSN |
| PTEN | (pp) | CAV1 |
| HIST2H2AA | (pp) | UBE2H |
| EIF3S10 | (pp) | EIF3S6 |
| HNF1-alpha | (pd) | C2 |
| FKBP8 | (pp) | PPP3CA |
| HSPA1A | (pp) | DNAJB1 |
| Smad2 | (pp) | AP1B1 |
| S100B | (pp) | PGM1 |
| CCL3 | (pp) | CCL3 |
| NAG-(1-4)NAG-(1-4)NAG-(1-4)NAG-(1-4)NAG-(1-4)NAG | (interactsWith) | CHI3L1 |
| STAT1 | (pp) | TNFRSF1A |
| S100A9 | (pp) | S100A9 |
| LIPE | (pp) | FABP4 |
| PDGFB | (pp) | PDGFB |
| CREB1 | (pp) | NR3C1 |
| NAG-(1-4)NAG-(1-4)NAG-(1-4)NAG-(1-4)NAG | (interactsWith) | CHI3L1 |
| SMAD3 | (pp) | SMAD3 |
| SMARCD1 | (pp) | NR3C1 |
| SHC1 | (pp) | ITGB4 |
| Unknown | (interactsWith) | ER |
| CAV1 | (pp) | CAV2 |
| TNPO1 | (pp) | RANBP2 |
| KCNAB1 | (pp) | DLG1 |
| SRC | (DirectedEdge) | PTPNS1 |
| STAT1 | (pp) | K-alpha |
| POU2F1 | (pp) | POU2F1 |
| S100B | (pp) | ANXA6 |
| SPEN | (pp) | RARA |
| HNF4-alpha | (pd) | CHI3L1 |
| NR3C1 | (pp) | TGFB1I1 |
| heme | (interactsWith) | HMOX1 |
| ELMO1 | (pp) | HCK |
| NAG-(4-1)NAG | (interactsWith) | PLAUR |
| Tat | (pp) | APP |
| 5-Residue_Peptide | (pp) | STAT1 |
| CTSD | (pp) | IGF2R |
| SELENBP1 | (pp) | AR |
| SMAD3 | (pp) | ZBTB16 |
| NR3C1 | (pp) | SMARCD1 |
| APOE | (DirectedEdge) | RARA |
| 1LYB_J | (pp) | CTSD |
| BCL2L11 | (pp) | BCL2 |
| ATM | (pp) | AP1B1 |
| AR | (pp) | BAG1 |
| Smad8 | (pp) | Smad2 |
| IL6 | (DirectedEdge) | SHC1 |
| FBLN1 | (pp) | FBLN1 |
| HNF4-alpha | (pd) | PPP1R3B |
| AR | (pp) | AR |
| SHBG | (pp) | SHBG |
| APP | (pp) | HADH2 |
| IL10 | (pp) | IL10RA |
| BNIP3L | (pp) | BCL2 |
| RNPS1 | (pp) | CSNK2A2 |
| XPOT | (pp) | RANBP2 |
| IL8 | (pp) | IL8 |
| NR4A1 | (pp) | BCL2 |
| S100A8 | (DirectedEdge) | S100A11 |
| VEGFB | (pp) | VEGFB |
| N-Acetyl-D-Glucosamine | (interactsWith) | CTSD |
| SHBG | (pp) | AP1B1 |
| IL2 | (pp) | SHC1 |
| XPOT | (DefaultEdge) | XPOT |
| STAT1 | (pp) | PIAS1 |
| AP1B1 | (pp) | ARF6 |
| NAG | (interactsWith) | CHI3L1 |
| BNIP3 | (pp) | BNIP3 |
| GDB | (interactsWith) | GSTM1 |
| HNF6 | (pd) | C2 |
| CSNK1D | (pp) | PER1 |
| ERBB2IP | (pp) | ERBB2IP |
| FGF2 | (pp) | SDC4 |
| SRC | (pp) | STAT1 |
| Smad4 | (pp) | GPNMB |
| APP | (pp) | APPBP2 |
| AR | (pp) | SMAD3 |
| TSC22D1 | (pp) | MTR |
| TNF | (DirectedEdge) | CCL5 |
| LRP1 | (pp) | PDGFB |
| SREBF2 | (pp) | SREBF2 |
| TLE1 | (pp) | MORF4L2 |
| RELN | (pp) | RELN |
| HNF4-alpha | (pd) | GOLGA2 |
| STAT5A | (pp) | SRC |
| 1BF5_B | (pd) | STAT1 |
| HYPA | (pp) | MMP11 |
| APPBP2 | (pp) | APPBP2 |
| AMP-PNP | (interactsWith) | HCK |
| PPM1B | (pp) | CDK6 |
| HNF4-alpha | (pd) | CLPTM1 |
| TYK2 | (pp) | STAT1 |
| SMAD5 | (DefaultEdge) | ID4 |
| TGF-beta-RI | (pp) | CSNK1D |
| CSF1 | (pp) | TNF |
| HMOX1 | (pp) | HMOX1 |
| IL10RA | (pp) | Viral_Interleukin-10_Homolog |
| LRP1 | (pp) | APOE |
| GPC1 | (DirectedEdge) | CHI3L1 |
| Unknown | (interactsWith) | AF15Q14 |
| AR | (pp) | ESR1 |
| NDN | (pp) | NDN |
| SHC1 | (pp) | SRC |
| POU2F1 | (pp) | ESR1 |
| IFNG | (DirectedEdge) | SHC1 |
| NR3C1 | (pp) | ZBTB16 |
| TRG20 | (pp) | POLR2E |
| RARA | (pp) | NR1H3 |
| HYPA | (pp) | PIAS1 |
| FKBP8 | (pp) | BCL2 |
| BMPR2 | (DefaultEdge) | CAV1 |
| S100B | (pp) | AGER |
| SNURF | (pp) | AR |
| DST | (pp) | COL17A1 |
| Tat | (pp) | TP53 |
| Smad4 | (pp) | PIAS1 |
| Unknown | (interactsWith) | FMNL1 |
| HNF4-alpha | (pd) | STAM |
| IL4 | (DirectedEdge) | SHC1 |
| BAG1 | (pp) | BCL2 |
| S100A8 | (pp) | S100A9 |
| SERPINE1 | (pp) | SERPINE1 |
| CTSD | (pp) | CTSD |
| RUSC2 | (pp) | RAB1B |
| NAG | (interactsWith) | PLAUR |
| CCL5 | (pp) | CCL5 |
| SMAD3 | (DefaultEdge) | SPTBN1 |
| DSCR1 | (DirectedEdge) | APP |
| SHBG | (pp) | CTSD |
| ISGF3G | (pp) | STAT1 |
| IL10RA | (pp) | Interleukin-10 |
| HNF4-alpha | (pd) | ACVR1 |
| HNRPU | (pp) | NDN |
| EIF3S3 | (pp) | EIF3S4 |
| Smad2 | (pp) | Smad4 |
| BAG1 | (pp) | NR3C1 |
| ER | (pd) | AP1B1 |
| COL17A1 | (pp) | COL17A1 |
| NDN | (pp) | TMEM33 |
| HNF4-alpha | (pd) | C2 |
| SMAD3 | (pp) | AR |
| ATM | (pp) | ATM |
| MOAP1 | (pp) | MOAP1 |
| NAG-(1-4)NAG | (interactsWith) | CHI3L1 |
| TNFRSF1A | (pp) | TRPC4AP |
| GSN | (pp) | AR |
| APOE | (pp) | PLTP |
| EIF3S4 | (pp) | EIF3S10 |
| Tat | (pp) | STAT1 |
| TGF-beta-RI | (pp) | RGS19 |
| NR3C1 | (pp) | CREB1 |
| SMARCD1 | (pp) | ESR1 |
| PPP1CB | (pp) | TMEM33 |
| HLA-G | (pp) | HLA-G |
| OC1 | (interactsWith) | S100A8 |
| VEGFB | (pp) | VEGF |
| FYN | (pp) | VAV1 |
| TNFRSF1A | (pp) | TNFRSF1A |
| NR3C1 | (pp) | SMAD3 |
| RBPMS | (pp) | RBPMS |
| IFNG | (pp) | IFNG |
| HSPA1A | (pp) | HSF1 |
| SHC1 | (pp) | LRP1 |
| UBE2H | (pp) | HIST2H2AA |
| TGFB1I1 | (pp) | NR3C1 |
| LRP1 | (pp) | SHC1 |
| PITPNA | (pp) | MORF4L2 |
| PER1 | (pp) | PER2 |
| EIF3S4 | (pp) | EIF3S3 |
| DAPK1 | (pp) | TNFRSF1A |
| GSH | (interactsWith) | GSTM1 |
| NAG-(4-1)NAG | (interactsWith) | CHI3L1 |
| SNURF | (pp) | ESR1 |
| FABP4 | (pp) | EXT2 |
| NCOA3 | (pp) | ESR1 |
| Trehalose | (interactsWith) | HMOX1 |
| RGS2 | (pp) | EIF3S6 |
| GSTM1 | (pp) | GSTM1 |
| B2M | (pp) | HLA-G |
| ESR1 | (pp) | CAV1 |
| Ca2+ | (interactsWith) | HCK |
| JUND | (DirectedEdge) | IL6 |
| STAT1 | (pp) | STAT1 |
| JARID1A | (pp) | NR3C1 |
| TYK2 | (pp) | TYK2 |
| Tat | (pp) | SMAD4 |
| SPRY1 | (DirectedEdge) | SHC1 |
| NR3C1 | (pp) | NCOA3 |
| JUND | (DirectedEdge) | IL8 |
| p621 | (pp) | AP1B1 |
| TNFRSF1A | (pp) | STAT1 |
| HNF6 | (pd) | AF15Q14 |
| AR | (pp) | POU2F1 |
| HNF4-alpha | (pd) | SREBF2 |
| NR2F6 | (pp) | NR2F6 |
