# Supplementary material for: Reconstruction and functional analysis of altered molecular pathways in human atherosclerotic arteries
Source: BMC Genomics. 2009 Jan 9;10:13. doi: 10.1186/1471-2164-10-13 (PMC2654039; doi:10.1186/1471-2164-10-13)
Supplement: Additional file 2 — Additional Figures. Figures included in this file include the functional classification of genes of the extra-cellular matrix, the functional classification of the atherogenes connected in the network of Figure 4a and the representation of the JAK/STAT pathway. [file 1471-2164-10-13-S2.doc]

**Additional Figures**

**Additional Figures Legends**

**Figure S1.** Functional classification of genes of the extra-cellular matrix. The view shows the relationships among highly related genes and their annotations. The two annotations (extracellular matrix – sensu Metazoa and extracellular matrix) present calculated p-values of 7.0E-3 and 2.8E-4, respectively. P-values were calculated by Fisher Exact test using DAVID tool. Collagen XXI is not represented because it is classified as signal peptide.

**Figure S2.** Graphic presentation of functional classification of the atherogenes connected in the network of Figure 4a, according to the molecular function categorization of Gene Ontology (GO). **a).** GO classes of the up-regulated genes; **b).** GO classes of the down-regulated genes. Colored bars represent p-value for each node calculated by BINGO Cytoscape plug-in.

**Figure S3.** Representation of the JAK/STAT pathway; modified from KEGG database.

**Additional Figure S1**

**
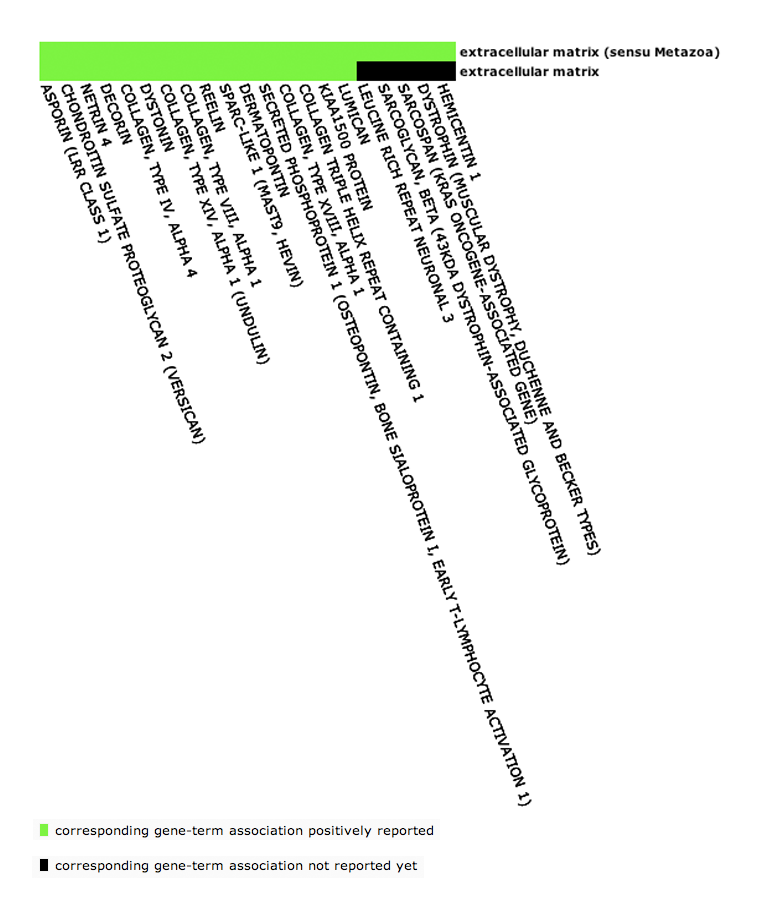
**

**Additional Figure S2**

**
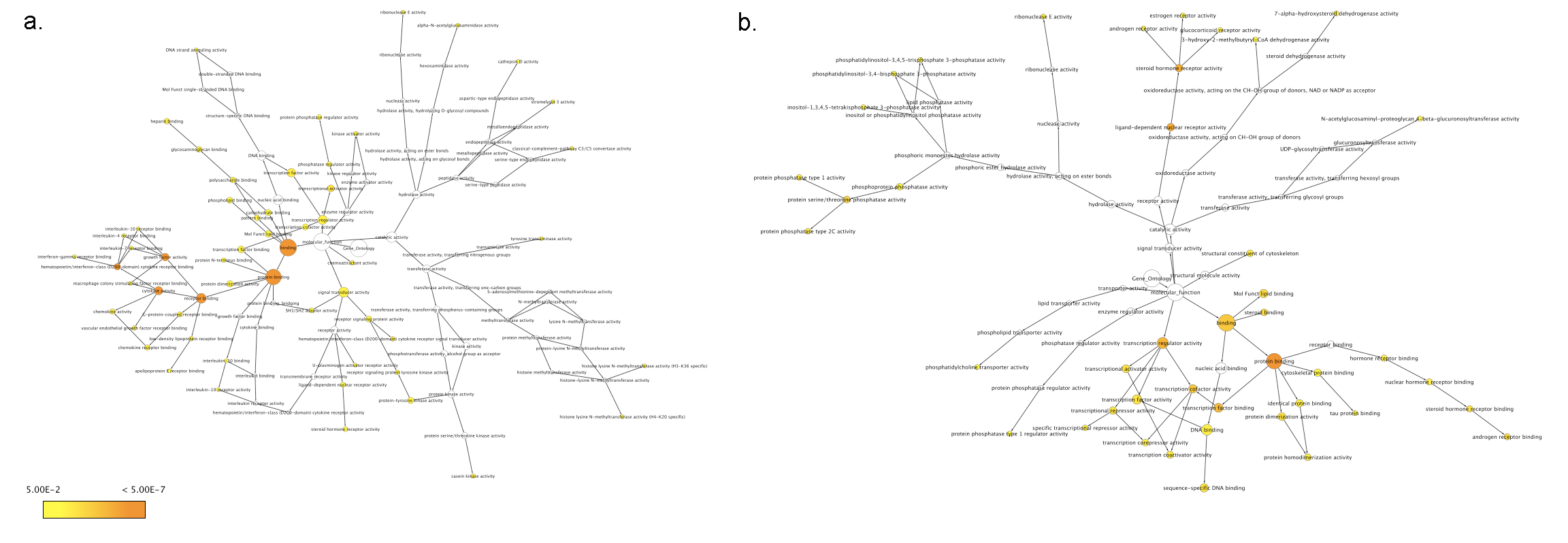
**

**Additional Figure S3**

**
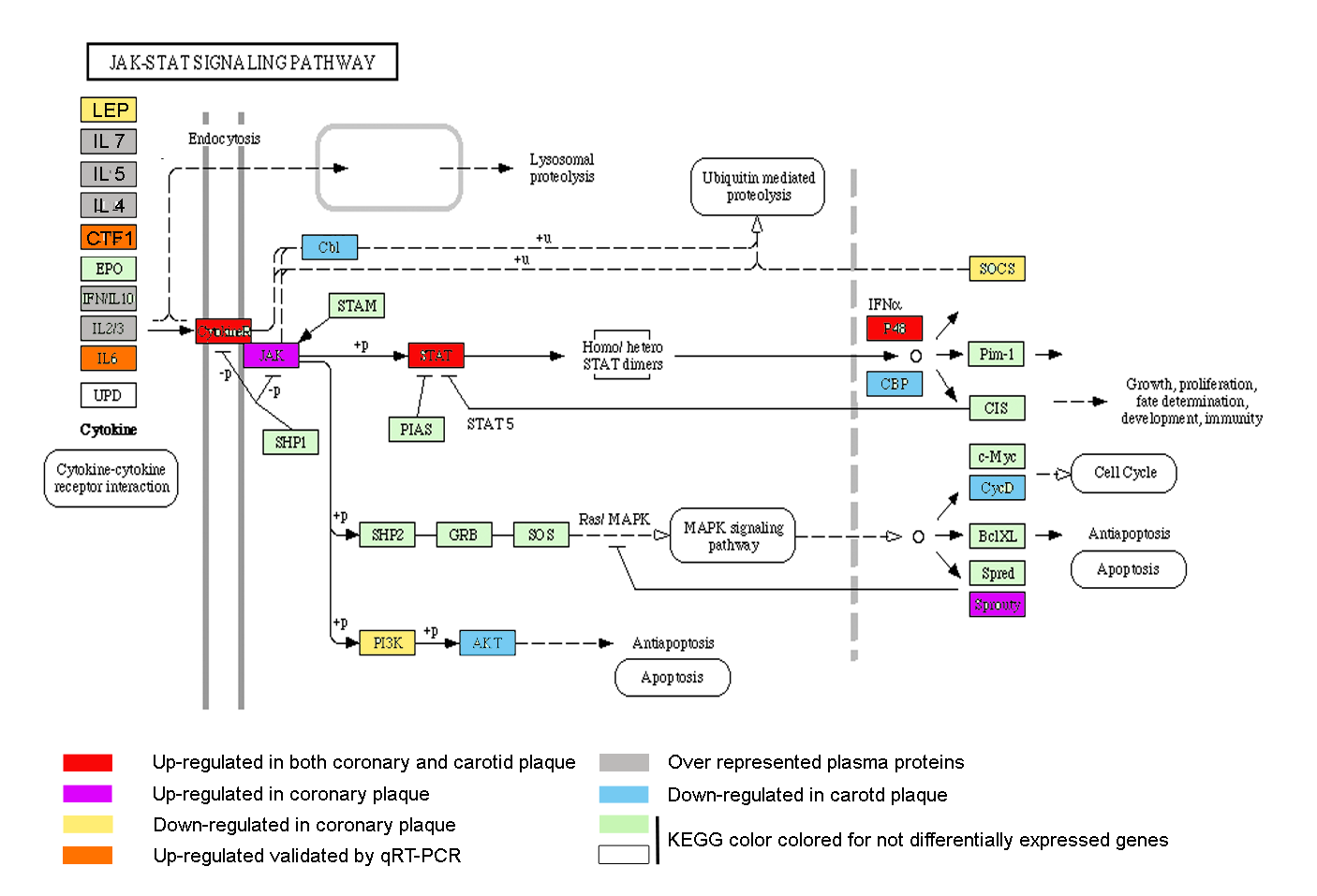
**
