# Supplementary material for: Reconstruction and functional analysis of altered molecular pathways in human atherosclerotic arteries
Source: BMC Genomics. 2009 Jan 9;10:13. doi: 10.1186/1471-2164-10-13 (PMC2654039; doi:10.1186/1471-2164-10-13)
Supplement: Additional file 3 — Expanded Methods. The data provided represent the expanded methods of the paper. [file 1471-2164-10-13-S3.doc]

**Expanded Methods**

**Coronary RNA extraction and gene expression experiments**

Visible plaques, adjacent to the segment utilized for histological analysis, were dissected from the entire coronary fragment and used separately for total RNA extraction with TRIzol (Invitrogen). 10 non-atherosclerotic coronaries were used as controls, and fragments with similar dimension and location, were used to extract RNA. RNA was quantified by UV adsorption in a NanoDrop ND-1000 spectrophotometer (Thermo Fisher Scientific) and analyzed for quality by capillary electrophoresis in an Agilent 2100 Bioanalyzer. Only RNA preparations showing no signs of even partial degradation were processed further. A RNA pool obtained by mixing equal aliquots of each control coronary RNA was utilized as a common reference sample. Control RNA pool and plaque RNAs (1 μg) were linearly amplified with MessageAmp™ aRNA amplification kit (Ambion) following manufacturer instructions, labeled with Cy3 and Cy5 dye (Amersham) and competitively hybridized to oligonucleotide microarrays platforms (GEO database ID: GPL6647) printed in-house by the CRIBI Microarray service (http://microcribi.cribi.unipd.it). For each plaque sample we performed two microarray experiments at least. Hybridizations were carried out in an automatic hybridization station (ArrayBooster, Advalytix) at 48°C for 30 hours, using hybridization solution containing 25% formamide. After stringent washings, fluorescence left bound to microarrays was read with the ScanArray LITE confocal laser scanner (PerkinElmer). Each microarray slide was subjected to three consecutive scans at low, medium and high settings of laser and photomultiplier. We therefore produced with this protocol six 16-bit tif images per microarray slide, grouped in low, intermediate and high intensity scans. Images were quantified with ScanArray Express (PerkinElmer) software using the fixed circle method. Data were normalized using the total and lowess methods, implemented in the MIDAW tool[1] and submitted to GEO database (GSE11138). Data filtering was made considering the values of “empty” control spots in the microarrays as outlined in GEO table (Table-description-for-experiments-filtering file). Data not in line with the above criteria were excluded from analysis. For each group of images obtained with different laser power, the genes with expression values in all experiments were analyzed by SAM program[2] implemented in the TMEV software[3].

The filtering process produced 6256, 6402 and 6323 features respectively from high, medium and low laser and photomultipler settings. Starting matrix presents 21888 features, as described in GEO platform GPL6647. SAM output analysis of a) high, b) medium and c) low scanning settings was charachterized respectively by 776 featuress (32.73% up-regulated; 67.27 down-regulated), 835 features (30.42% up-regulated; 69.58% down-regulated) and 865 features (29.48% up-regulated; 70.52% down-regulated). These lists of features have been uploaded as Supplementary files in GEO database as described above.

Finally, the lists of differentially expressed genes obtained from the analysis of the three groups microarray images and presenting false discovery rate of 0, were integrated[4].

All microarray experiments are fully described in the corresponding submission of GEO database in which there are reported supplementary information including: *i)* Patients and controls descriptions; *ii)* Six files (*Scan-high-down-regulated-SAM*, *Scan-high-up-regulated-SAM*, *Scan-low-down-regulated-SAM*, *Scan-low-up-regulated-SAM*, *Scan-medium-down-regulated-SAM* and *Scan-medium-up-regulated-SAM*) that contain the lists of differentially expressed genes obtained by the analysis of the microarray images performed with diverse scanning parameters; *iii)* A file (*Table-description-for-experiments-filtering*) which outlines the parameters used for microarray data filtering; *iv)* Two files (*Union-down-regulated-atero-SAM* and *Union-up-regulated-atero-SAM*) describing the integration[4] of the lists of differentially expressed genes.

**Meta-analysis**

Integrated gene expression data from coronaries were compared with those derived from the analysis of carotid plaque. Data obtained with the Affymetrix GeneChip Human Genome U95Av2 (http://www.affymetrix.com/products_services/arrays/specific/hgu95.affx) on gene expression of carotid plaque were retrieved from Array Express databases (E-MEXP-268) and compared with normal coronary profiles (GSE3526, GSE7307) performed with the Affymetrix U133 plus 2.0 platform (http://www.affymetrix.com/products_services/arrays/specific/hgu133plus.affx). Each microarray data batch was normalized separately in d-chip software using the invariant probe set normalization method. Common genes between the two platforms were used to construct a metafile based on the “common probe set file” supplied by Affymetrix. This file comprises 10747 features and can be downloaded at http://www.biostat.harvard.edu/complab/dchip/combine%20 chip.htm. All experiments were normalized a second time with the invariant probe set method to balance differences between microarray platforms and used to detect differentially expressed genes. Parameters used to detect differentially expressed genes were: 1) fold change higher than 1.6; 2) lower expression data cutoff (E – B > 100 and B – E > 100 where E and B is the mean of the gene expression values in the plaque and in the controls respectively); and 3) t-test between samples (p <= 0.05 for t-test). List of differentially expressed genes resulted from this analysis was submitted as a supplementary file to GEO database (GSE11138) and named *GSE3526-GSE7307-asym-symp-Meta-analysis*. Affymetrix identificative names of differentially expressed genes were converted in Entrez Gene IDs by DAVID tool and compared with differentially expressed genes identified in the coronary plaque. This analysis allowed the compilation of lists of Entrez Gene entries for genes that resulted a) differentially expressed in both carotid and coronary plaques (“atherogenes”) and b) specifically differentially expressed in each of e two analyzed arteries. These lists were also submitted to GEO database with independent files that are: 1) *commmon-down-Meta-analysis* (list of genes down-regulated in both carotid and coronary vessels); 2) *commmon-up-Meta-analysis* (list of genes up-regulated in both carotid and coronary vessels); 3) *Specific-Carotid-Down-Meta-analysis*, *Specific-Carotid-Up-Meta-analysis*, *Specific-LAD-Down-Meta-analysis*, *Specific-LAD-up-Meta-analysis* (lists of genes specifically down- or up-regulated in either carotid or coronary vessels.

**Network construction and analysis**

The list of coronary (a) and carotid plaques (b) differentially expressed genes or of proteins (c) whose concentration was significantly changed in atherosclerotic patients respect to controls, was used to retrieve interactions between them and other elements. We used two interaction databases: the Biomolecular Interaction Network Database (BIND)[5] and the Biological General Repository for Interaction Datasets (BioGRID)[6] and implemented the results with literature evidences. BIND and BioGRID are freely accessible databases of records documenting protein interaction, molecular complexes and pathways. The sum of all such interactions defines the global regulatory network of the cell and for this reason is usefull to understand processes involved in a particular pathological condition.

Genes without interaction were filtered out and networks containing up-regulated genes were matched with one containing down-regulated genes. Interaction network is described in the additional Table S3 and is drawn in Figure 4A.

Molecular functions for genes involved in this network were identified using BiNGO[7] Cytoscape plug-in results are plotted in the additional Figure S2. BiNGO is a Java-based tool to determine which Gene Ontology (GO) categories are statistically overrepresented in a set of genes or a subgraph of a biological network. BiNGO maps the predominant functional themes of a given gene set on the GO hierarchy, and outputs this mapping as a Cytoscape graph. In our computations we used a hypergeometric test and the Benjamini and Hochberg False Discovery rate (FDR) correction with 0.05 significance level.

Identified network was used to determine high interconnected nodes with MCODE[8] Cytoscape plug-in. MCODE software helps to find cluster of interconnected nodes to identify proteins complex or parts of same pathway or co-regulated pathways. MCODE is a relatively fast method of clustering, although it does not currently provide statistical scores for the resulting clusters, but it can be used as discovery tool when analyzing a network.

**References for Expanded Methods**

1. Romualdi C, Vitulo N, Del Favero M, Lanfranchi G: **MIDAW: a web tool for statistical analysis of microarray data**. *Nucleic Acids Res* 2005, **33**(Web Server issue):W644-649.

2. Tusher VG, Tibshirani R, Chu G: **Significance analysis of microarrays applied to the ionizing radiation response**. *Proc Natl Acad Sci U S A* 2001, **98**(9):5116-5121.

3. Saeed AI, Sharov V, White J, Li J, Liang W, Bhagabati N, Braisted J, Klapa M, Currier T, Thiagarajan M *et al*: **TM4: a free, open-source system for microarray data management and analysis**. *Biotechniques* 2003, **34**(2):374-378.

4. Skibbe DS, Wang X, Zhao X, Borsuk LA, Nettleton D, Schnable PS: **Scanning microarrays at multiple intensities enhances discovery of differentially expressed genes**. *Bioinformatics* 2006, **22**(15):1863-1870.

5. Gilbert D: **Biomolecular interaction network database**. *Brief Bioinform* 2005, **6**(2):194-198.

6. Breitkreutz BJ, Stark C, Reguly T, Boucher L, Breitkreutz A, Livstone M, Oughtred R, Lackner DH, Bahler J, Wood V *et al*: **The BioGRID Interaction Database: 2008 update**. *Nucleic Acids Res* 2008, **36**(Database issue):D637-640.

7. Maere S, Heymans K, Kuiper M: **BiNGO: a Cytoscape plugin to assess overrepresentation of gene ontology categories in biological networks**. *Bioinformatics* 2005, **21**(16):3448-3449.

8. Bader GD, Hogue CW: **An automated method for finding molecular complexes in large protein interaction networks**. *BMC Bioinformatics* 2003, **4**:2.
